# An open-source eye-tracking system adaptable to diverse terrestrial vertebrates reveals state-dependent visual acquisition in lizards

**DOI:** 10.1371/journal.pbio.3004066

**Published:** 2026-11-03

**Authors:** Nimrod Leberstein, Tali Remenick, Milan Becker, Mark Shein-Idelson

**Affiliations:** 1 School of Neurobiology, Biochemistry, and Biophysics, Tel Aviv University, Tel Aviv, Israel; 2 Sagol School of Neuroscience, Tel Aviv University, Tel Aviv, Israel; University of Sussex, UNITED KINGDOM OF GREAT BRITAIN AND NORTHERN IRELAND

## Abstract

Gaze shifts are fundamental to visual exploration and processing, yet empirical knowledge is largely confined to a small number of model species, limiting our understanding of general principles of visual acquisition across animals. Here, we introduce a versatile, low-cost, and open-source framework design for eye-tracking in small, freely moving animals. Leveraging photogrammetry, 3D modeling, and 3D-printing, we produce headmounts adaptable to diverse head-eye geometries, as we demonstrate for lizards, turtles, and mice. Detailed recordings in Pogona vitticeps, a laterally eyed ectotherm with a high-acuity retina, reveal a rich oculomotor repertoire with kinematics closely resembling the saccadic main sequence in primates. We found both movement-coupled large binocular saccades, which exhibited a strong axial bias as well as small, isotropic, and monocular saccades suggesting a capacity for visual exploration of local details. Visual acquisition strategies were strongly tied to behavioral states with periods of active sampling (3–5 Hz saccades) separated by periods of ocular stillness lasting many seconds and suggesting a visual acquisition strategy distinct from those commonly described in endothermic model species. Furthermore, active states were correlated with pupil constriction in contrast to mammals and similarly to birds. Our results demonstrate the utility of our experimental approach for understanding visual acquisition across terrestrial vertebrates. Such a multi-perspective view on natural visual acquisition across species will be essential for developing a generalized understanding of vision, its ecological functions, and its evolutionary trajectory.

## Abstract 2

Visual acquisition relies on coordinated gaze shifts, yet our understanding of eye movement strategies remains limited to a few model species. This study develops head mounts adaptable to the head-eye geometries of lizards, mice and turtles, revealing diverse state dependent eye movements and visual sampling behaviors.

## Introduction

Shifting gaze is fundamental to visual exploration and processing [1,2]. Visually guided gaze shifts selectively acquire information from specific regions of the visual environment. In foveated animals [3], this process is particularly precise and informative, offering a unique window into the dynamic prioritization of goals across contexts [4,5]. In most studies, gaze information is acquired under head-restrained conditions [6–10]. This setting facilitates precise gaze measurements in controlled conditions, yielding significant insights into visual processing [11–17]. However, under natural conditions, visual exploration involves a dynamic coordination between eye, head, and body movements, which is disrupted under head fixation [18–20]. This discrepancy limits our understanding of visual acquisition strategies.

Over the last two decades, new head-mounted systems enabled eye-tracking in head-free conditions for humans and primates [21]. These technologies provided valuable insights into the integration of eye movements with broader behavioral patterns [22], such as describing a tight predictive coupling between gaze and gait in humans [23], or showing that hippocampal activity is highly tuned to 3D head orientations and eye movements in macaques [24]. However, these systems are tailored to large heads and not readily compatible for use in smaller model species. Recently, innovative miniature video- and magnetic-based systems were developed, substantially expanding our access to eye measurements in freely moving small animals [19,25–34]. The first video implementation allowed oculography in freely moving rats through bespoke camera and support modules [25], whereas later mouse-oriented systems further miniaturized the hardware and, in some cases, provided open-source solutions, improving accessibility [26,27,30]. These were complemented by miniaturized magnet-sensor trackers that brought magnetic eye-tracking into freely moving small animals without field-bound search coils [28,29]. Still, these valuable systems were typically tailored for a particular species, and rely on nonflexible anchor positions, often tied to chronic electrophysiology or magnetic implants [25–28,30–32,35,36]. Consequently, these did not offer an accessible route for repositioning the optical modules in species with markedly different eye-head geometries, such as broad-skulled, highly lateral-eyed lizards, or head-retracting turtles [37]. This has constrained comparative analyses of visual acquisition across taxa, which is crucial for gaining an evolutionary understanding [38,39].

A particularly important stage in the evolution of visual acquisition is the transition to terrestriality. This transition was accompanied by a major expansion in the potential visual range. Correspondingly, vertebrates evolved larger eyes with higher acuity [40,41]. Following the emergence of amniotes, vertebrates evolved a distinct neck and the atlas-axis bone complex which allowed flexible head movements dissociated from body movement, increased visual sampling range, and improved stabilization during self-movement [42]. By contrast, the available evidence from amphibians indicates minimal eye movements, and increased reliance on full body turns for visual orienting [43]. However, the evolution of neural machinery for sampling complex terrestrial visual environments remains uncharted [38,39,44]. Reptiles, the sister class to mammals within amniotes provide a critical comparative perspective on this question [45,46].

Multiple reptile species exhibit complex visual behaviors and possess steep-walled foveae and visual streaks associated with high spatial acuity [47,48]. A limited number of studies in lizards examined visual behaviors. These focused on optokinetic reflexes and prey orientation, without recording eye movement or performing visual sampling analyses [49–51]. The only quantitative eye-movement datasets in nonavian reptiles are from chameleons. Magnetic eye-tracking revealed a high oculomotor range, a monocular eye coordination regime that changes with behavioral context, and a linear relationship between saccade amplitude and speed during prey capture [52,53]. Thus, the spatial and temporal characteristics of visual sampling in nonavian reptiles remain largely unknown [52,54].

To bridge this gap, we developed a flexible system for measuring eye movements in freely moving small animals. Inspired by the use of open-source components for miniaturized video acquisition of the eyes [26], we developed an optical system and a personalization approach which allows positioning the optic modules for diverse head-eye geometries in a variety of animals. Using photogrammetry, we generated 3D-printed headmounts that can be adapted to specific species and subjects, ensuring a precise and stable fit while maintaining minimal interference with natural behavior. We first demonstrate the system’s versatility by acquiring eye video in the lizard *Pogona vitticeps*, the turtle *Trachemys scripta elegans*, and *Mus musculus*. Next, focusing on *P. vitticeps*, a foveated visually orienting, laterally eyed agamid lizard [47,55], we address key questions in visual acquisition. We ask whether agamid lizards exhibit visually guided ocular saccades. We examine whether visual acquisition strategies in *P. vitticeps* differ from patterns described in endotherms across long time scales. We probe the relation between pupil diameter and behavioral states. Studying these questions revealed a complex pattern of visual gaze shifts and lateralized behaviors, as well as state-dependent changes in visual acquisition and pupil diameter in *P. vitticeps*.

## Results

### System design, fitting, and adaptability

To monitor eye movements in freely behaving small animals, we developed a flexible, open-source, low-cost, eye-tracking approach that is applicable across species (Materials and methods, Table 1). A major challenge in implementing eye-tracking systems across individuals and species is posed by the diversity in head-eye geometries. To address this, we designed personalized headmounts that are 3D printed based on a 3D model of the animal’s head and eyes as scanned using photogrammetry (Fig 1A and 1B). Importantly, this scan followed a surgical implantation of a single hexagonal screw anchor point for connecting the headmount (Figs 1 and S7; Materials and methods). The model is used to define the desired camera-eye geometry in virtual space (Autodesk Fusion) and produce a single-piece headmount to connect with the anchor, support cameras, and the infrared (IR) mirrors reflecting the eyes (Fig 1B; Materials and methods). The relevant features for virtually fitting the camera position to each animal, are the geometrical relationship between the scanned implanted anchor (that is connected to the skull) and the eyes, together with the head geometry required to verify an unobstructed optical path between the eye, mirror, and camera. Estimation of the skull-eye relationship is therefore not required. The headmount is 3D printed as a single piece (without the mirror holders which are printed separately with interlocking connectors), resulting in a known and reproducible eye-camera geometry. When a photogrammetry scan is impractical, or when the experiment requires a shorter turnaround time by bypassing the virtual fitting and printing process of a single-piece headmount, the components can instead be pre-printed as individual interlocking modules which form a modular solution kit, such that the fitting procedure can be performed directly on the animal (Fig 1D; Materials and methods). To achieve sharp eye images at short working distances without background contamination, the cameras were fitted with close-focus lenses and IR-pass filters, with two IR LEDs providing stable illumination of each eye (Fig 1C; Materials and methods). This setup allowed video acquisition at 640x480 pixels and 60 frames/s with stable illumination of the eye and synchronization verification via programmed LED power-off windows (Materials and methods). The complete headmount with LEDs, wires, and cameras (2.13g each) weighed between 3.5–10 g depending on animal size. Before recording, headmounts were connected to the animal and counterbalanced by a pulley system holding the headmount and connecting cables. To assess the stability of the system, we quantified camera jitter (spatial cross-correlation peak shift between the first frame and other frames calculated on a stationary image segment) during free behavior (which included locomotion). The typical measured jitter was small (overall span 0–200 µm at the imaged eye plane with a median of 42 µm (~1 pixel) for rigid lizard headmounts) (Figs 1E and S9) and could be corrected by subtracting the jitter amplitude.

**Table 1:** Table of system components and software.

Reagent or resource	Source	Identifier
**Software and algorithms**		
Python 3.10	Python Software Foundation	https://www.python.org
MATLAB 2025a	MathWorks	https://www.mathworks.com
Blender 4.4	Blender Foundation	https://www.blender.org
Custom code for eye camera acquisition and analysis	This paper	https://github.com/EvolutionaryNeuralCodingLab/PETS
Autodesk Fusion (computer-aided design)	Autodesk	https://www.autodesk.com/products/fusion-360/overview
DeepLabCut (pose estimation)	Mathis and colleagues	https://github.com/DeepLabCut/DeepLabCut
Meshroom (Photogrammetry)	Alice Vision	https://alicevision.org/#meshroom
**System components**		
3D printer (Form 2)	Formlabs	https://formlabs.com/3d-printers/form-2/
3D printer resin GreyV4.1	Formlabs	https://formlabs.com/global/products/grey-resin-v4-1/
Arena cameras (FLIR Firefly FFY-U3-16S2M-DL)	Teledyne FLIR	https://www.flir.com/products/firefly-dl
Electrophysiology recording system (Open Ephys Acquisition Board)	Open Ephys	https://open-ephys.org
Head-mounted accelerometer (embedded in Intan RHD2132 headstage)	Intan	https://intantech.com/RHD_headstages.html?tabSelect=RHD32ch
Raspberry Pi 4 Model B	Raspberry Pi	https://www.raspberrypi.com/products/raspberry-pi-4-model-b/
Eye cameras (5MP OV5647 Miniature Camera Module with Long Flex Cable for Raspberry Pi (NoIR))	Arducam	https://www.arducam.com/1-4-5-noir-mpspy-camera-module-sensor-flex-cable-raspberry-pi.html
Manual focus lenses for eye cameras used for lizards and turtles (3.7 mm, M7 thread, f/2.55)	QILens, AliExpress	https://www.aliexpress.com/item/32944505429.html?spm=a2g0o.detail.1000013.3.2e642956MO1ZOF&gps-id=pcDetailBottomMoreThisSeller&scm=1007.13339.274681.0&scm_id=1007.13339.274681.0&scm-url=1007.13339.274681.0&pvid=84441b1c-37b9-4c65-914c-92ae5767dbb2&_t=gps-id%3ApcDetailBottomMoreThisSeller%2Cscm-url%3A1007.13339.274681.0%2Cpvid%3A84441b1c-37b9-4c65-914c-92ae5767dbb2%2Ctpp_buckets%3A668%232846%238111%231996&pdp_ext_f=%7B%22sku_id%22%3A%2212000026973414186%22%2C%22sceneId%22%3A%223339%22%7D&pdp_npi=2%40dis%21ILS%21%2125.63%21%21%21%21%21%40211b5e2416533887292982240e45f3%2112000026973414186%21rec&gatewayAdapt=glo2isr
Manual focus lenses for eye cameras used for mice (4.5 mm, M7 thread, f/2.8)	QILens, AliExpress	https://he.aliexpress.com/item/32988576525.html?spm=a2g0o.productlist.main.2.7703paM0paM05l&algo_pvid=7ab6577c-953b-448b-9bc8-0d0aeee60aa3&algo_exp_id=7ab6577c-953b-448b-9bc8-0d0aeee60aa3-1&pdp_ext_f=%7B%22order%22%3A%227%22%2C%22eval%22%3A%221%22%2C%22fromPage%22%3A%22search%22%7D&pdp_npi=6%40dis%21ILS%2163.66%2163.66%21%21%2120.59%2120.59%21%402103810f17890301036207102e0fc5%2112000048219348224%21sea%21IL%210%21ABX%211%210%21n_tag%3A-29910%3Bd%3A49a60c2%3Bm03_new_user%3A-29895&curPageLogUid=ta2Lcm3mRtx1&utparam-url=scene%3Asearch%7Cquery_from%3A%7Cx_object_id%3A32988576525%7C_p_origin_prod%3A
IR mirror (8 mm × 8 mm × 0.7 mm 650nm IR-Cut Blocking Filter)	Quanmin, Amazon	https://www.amazon.com/Quanmin-8mm%C3%978mm%C3%970-7mm-Blocking-Multi-coating-Low-Pass/dp/B07C6QZDGP?ref_=ast_sto_dp&th=1
930 nm, 100 × 100 × 1 mm PMMA IR-pass filter (cut to 1 mm diameter disks and fitted on camera lenses)	Nantong Foric Optical Glass Factory Store, AliExpress	https://www.aliexpress.us/item/3256802298233543.html?spm=a2g0o.productlist.0.0.43a83539dgtnKS&algo_pvid=a3908809-fe36-4c5d-a3e9-04b178ed420c&algo_exp_id=a3908809-fe36-4c5d-a3e9-04b178ed420c-3&pdp_ext_f=%7B%22sku_id%22%3A%2212000020821017837%22%7D&pdp_npi=2%40dis%21ILS%2139.65%2139.65%21%21%21%21%21%402100bddd16605595435317828eaf5c%2112000020821017837%21sea&curPageLogUid=AlpqBRrmjbQH&gatewayAdapt=glo2usa4itemAdapt#nav-store
M7 Lens holder height 6.0 mm	QILens, AliExpress	https://he.aliexpress.com/item/1005003151503038.html?mp=1&gatewayAdapt=glo2isr
Programmable LED driver (Cyclops)	Open Ephys	https://open-ephys.org/cyclops
High Speed Infrared Emitting Diodes, 940 nm vsmb2943rgx01	Vishay Semiconductors	https://www.vishay.com/en/product/83486/
Brass hexagonal standoff, M3, 6 + 6 mm F/M (lizard and turtle cranial anchor)	Uxcell store, Amazon	https://www.amazon.com/uxcell-Standoff-Spacer-Screws-Pillar/dp/B07KWVD986?language=en_US&currency=ILS
Nylon hexagonal standoff, M2, 12 mm F/F (mouse cranial anchor)	XT Hardware Store, AliExpress	https://he.aliexpress.com/item/1005007582260863.html?src=google&src=google&albch=shopping&acnt=742-864-1166&isdl=y&slnk=&plac=&mtctp=&albbt=Google_7_shopping&aff_platform=google&aff_short_key=UneMJZVf&gclsrc=aw.ds&albagn=888888&ds_e_adid=&ds_e_matchtype=&ds_e_device=c&ds_e_network=x&ds_e_product_group_id=&ds_e_product_id=he1005007582260863&ds_e_product_merchant_id=5379387784&ds_e_product_country=IL&ds_e_product_language=iw&ds_e_product_channel=online&ds_e_product_store_id=&ds_url_v=2&albcp=22664428499&albag=&isSmbAutoCall=false&needSmbHouyi=false&gad_source=1&gad_campaignid=22668076981&gclid=CjwKCAjwqonVBhA4EiwA9wYJ3Vkjrw4slf4VISkUzJ3SpbP7zOuJvyAyR0l5zoeqYZ_HzzGM7-vxRRoC0dkQAvD_BwE&gatewayAdapt=glo2isr#nav-store
UV adhesive (Fusion-Flo Light Cured Nano Hybrid Flowable Composite)	Prevest DenPro	https://www.prevestdenpro.com/product/fusion-flo/
Dental Flux (Gel de Acido grabador azul)	DentaFlux	https://www.dentaflux.com/producto/acido-fosforico-37-miniacid-4x12ml-azul/
UV adhesive (Evetric Bond)	Ivoclar	https://www.ivoclar.com/hr_hr/products/adhesives/evetric-bond
UV adhesive (Transbond XT)	3M Unitek	https://www.3mindia.in/3M/en_IN/p/d/b00007525/
Coral-Fix Dental Cement	Ron Dental Depo	https://rons.co.il/product/coral-fix/
Photogrammetry acquisition camera (Sony RX100-ii)	Sony	https://www.amazon.com/Sony-Premium-Compact-Multi-Interface-DSCRX100M2/dp/B00DM8R866

**Fig 1:**
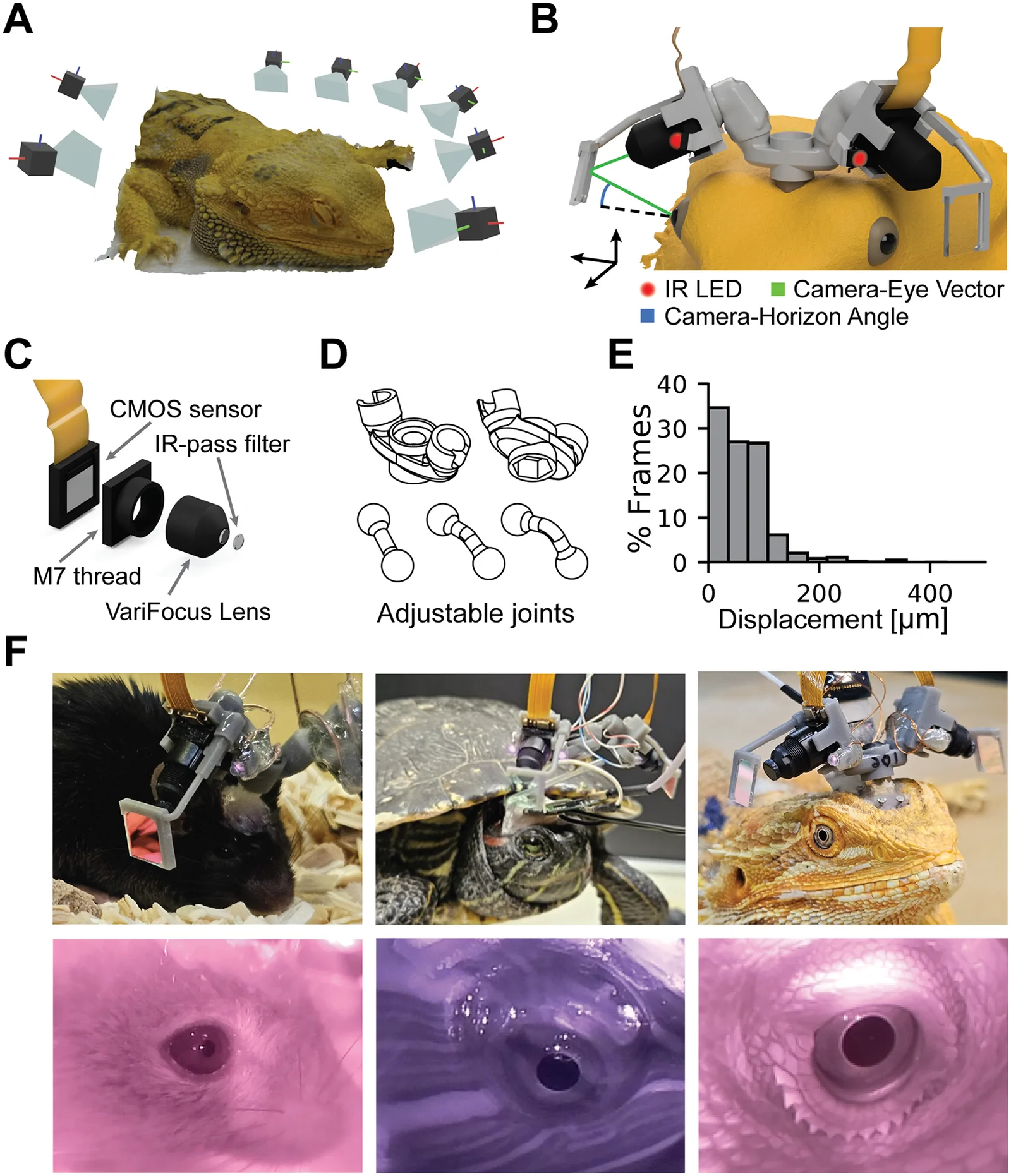
A model-based approach for 3D printing personalized headmounts for eye-tracking in small freely behaving animals. **(A)** 3D animal model acquired using photogrammetry by combining multiple camera viewing angles (a subset shown for illustration). **(B)** A lizard’s head (3D rendered) fitted with a full single-print camera headmount (gray). A virtual fitting procedure is used to calculate the camera (black) and mirror position such that a light beam (green line) from the camera center reaches the eye center. The inclination angle is determined by an animal-centric horizon defined as a line connecting the two eye centers (dashed black). Inclination angles fluctuate between 20 and 30 degrees for different animals. After 3D printing the headmount, two IR LEDs (red) are glued to each camera holder to provide illumination at 940 nm, and an IR reflector is glued to an alignment groove on the mirror holder. **(C)** The lens assembly of purchased minicameras is removed and replaced by a long M7 thread and 3.7 mm manual focus lens to enable variable working distance. The lens is fitted with an IR-pass filter to remove image contamination by visible light. **(D)** Diagrams for a modular solution kit. Different ball-joint arms allow a flexible and adjustable fit for variable head geometries without the need for 3D scanning. **(E)** Histogram of the camera movements, quantified as percentage of overall frames in each bin, indicating low camera jitter (over 90% of frames under 200 µm). The data underlying this graph can be found in S1 Data. **(F)** Examples of personalized headmounts for a mouse (*Mus musculus*), turtle (*Trachemys scripta elegans*), and lizard (*Pogona vitticeps*) with corresponding raw camera images acquired from the eye.

To demonstrate the system’s versatility, we used it to successfully record eye movements in three distinct model animals: bearded dragons (*Pogona vitticeps*), red-eared slider turtles (*Trachemys scripta elegans*), and mice (*Mus musculus*). These species were chosen for their anatomical and behavioral diversity (Fig 1F): *P. vitticeps* lizards are typified by wide heads and lateralized eyes; turtles posed a unique challenge due to their ability to retract and extend their heads into their shells; and mice are small and abundant in research. To enable measurement in turtles, we incorporated an extender between the anchor point and the headmount connector, allowing full range of head movement by keeping the headmount outside the shell when the head is retracted [56]. The longer working distance between the eyes and cameras was compensated by the manual focus of the modified lenses. To accommodate the smaller size and weight of mice, we further miniaturized the headmount to 3.5 g and replaced the brass hexagonal anchor with a lightweight plastic version. To enlarge the area of the smaller mouse eye on the sensor, we used longer-focal-length lenses (4.5 mm) with a shorter minimum working distance, producing a narrower field of view and a larger eye image within the camera frame. We define successful recording as the ability to acquire long (20–60 min) eye-tracking videos during unrestrained behavior, with low camera jitter and a large eye image relative to the camera frame (representative eye data traces are shown in S8A Fig). At 640 x 480 pixels resolution, the eye ROI occupied 61.8% of the frame diagonal in lizards, 40.1% in mice, and 39.1% in turtles (mean manually annotated ROI diagonals: 495, 321, and 313 pixels, respectively). Recordings were obtained repeatedly over weeks to months from individual animals, typically once every 2–4 days, with viable recording periods of up to 6 months in *P. vitticeps*, 2 months in *T. scripta elegans*, and 4 weeks in mice. We compare system technical specifications against previously published video oculography works in S1 Table.

### Eye movements repertoire in freely behaving *P. vitticeps*

The intricate musculature and neural circuitry controlling vertebrate eye movements enable diverse oculomotor behaviors that reflect visual acquisition strategies [2]. Yet, limited measurements in freely moving conditions and across species hinder the derivation of general principles [7]. To study the oculomotor behavior in *P. vitticeps*—a visually orienting agamid lizard with highly lateralized eyes—we acquired bilateral eye videos from freely exploring animals (*n* = 5) placed in a behavioral arena (S1 Video) [57,58]. Eye videos were used to reconstruct egocentric gaze vectors (eye-in-head angles) from pupil annotations using a weak perspective model (Fig 2A) [25,33,35,36,59,60], validated using a 3D Blender-based [61] simulation of the eye-camera system (Materials and methods; S1A Fig). Because head posture relative to the environment was not reconstructed, our analyses quantify relative eye-in-head positions rather than absolute gaze direction in world coordinates. We therefore used the 3D simulation to quantify the relative eye-in-head estimation errors (|Δ|) in φ (mean = 0.84 ± 0.48°, range 0.42–1.67°, std = 0.95 ± 0.42°) and θ (mean = 1.19 ± 0.48°, range 0.65–1.93°, std = 1.18 ± 0.51°) (Materials and methods; S1B Fig).

**Fig 2:**
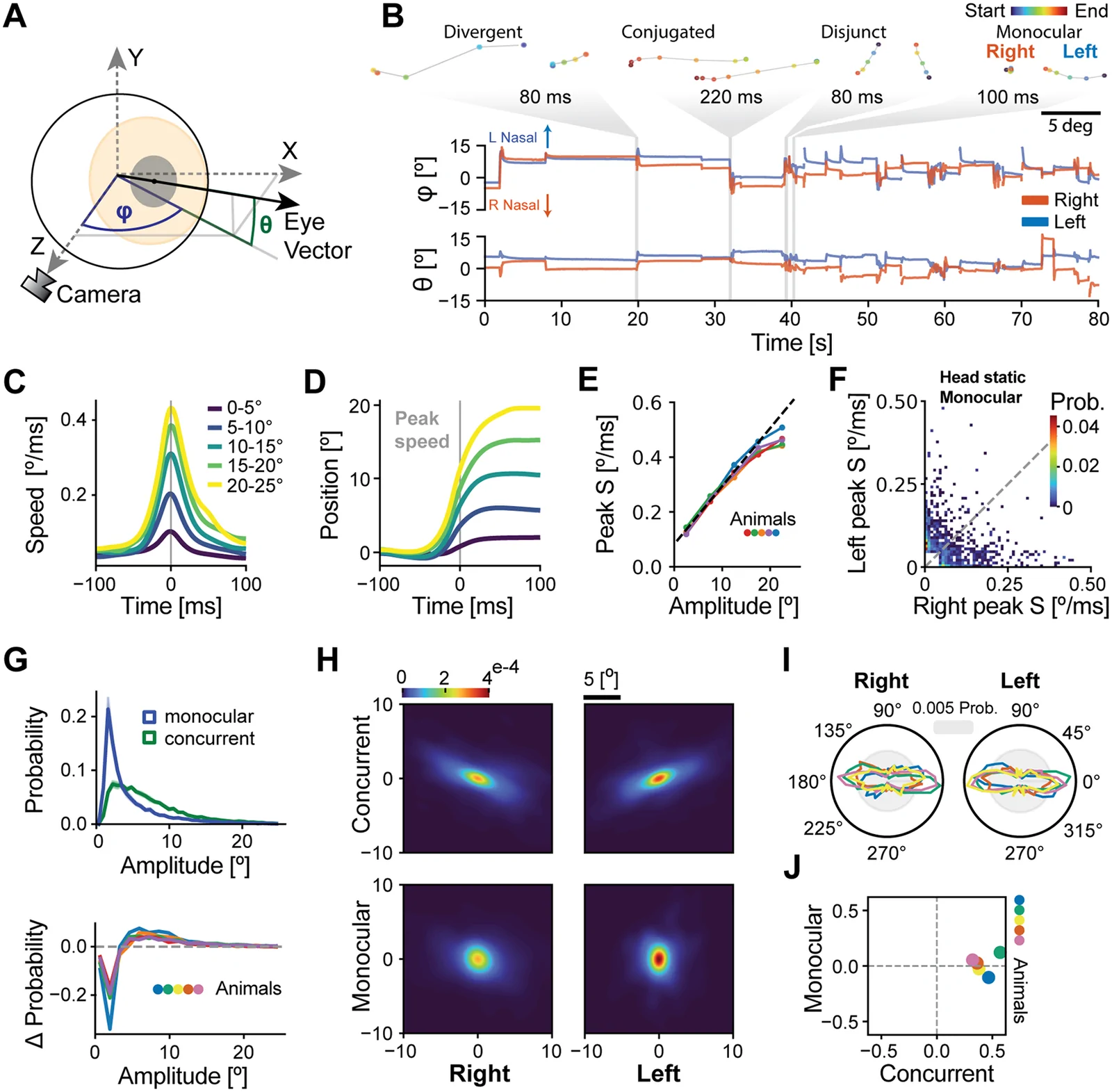
Main saccadic sequences with isotropic monocular and directionally biased concurrent saccades in *P. vitticeps.* **(A)** Schematic of calculating viewing angle in camera coordinates. 2D data points from eye videos were converted to 3D directions defined as azimuth (φ) and elevation (θ) angles (Materials and methods). **(B)** Simultaneous eye angle tracking for both eyes ((φ,θ) = (0,0) is the eye’s median position). (Top) The spatial trajectory of four example saccades of different types. left to right: divergent, conjugated, disjunct, and monocular dynamics. **(C)** Mean saccade angular speed profiles for one animal (*n* = 6,954 saccades). Events were binned by net amplitude (5° bins, color coded), aligned to t = 0 ms (peak speed), and smoothed with a 10 ms gaussian kernel. **(D)** Mean angular-displacement traces (from (C)) along each saccade’s movement axis, aligned as in (C). **(E)** Relationship between saccade amplitude and peak angular speed. Each colored line represents one animal (*n* = 5; 34,876 saccades). Linear fit y = 0.021 [ms⁻¹] × amplitude [°] + 0.083 [°/ms] (R^2^ = 0.44, r = 0.66, *p* < 5e−10). **(F)** Inter-ocular peak-speed coupling for monocular, head stationary saccades. Two-dimensional probability histogram of right- vs. left-eye peak angular speed [°/ms] for monocular saccades during head-stationary epochs pooled across animals (equal-animal weighting). **(G)** (Top) Independently normalized probability distributions of saccade amplitudes for concurrent (green) and monocular (blue) events. Mean (±S.E.M shading) animal-wise probability distributions (0-25°). (Bottom), Per-animal difference curves (concurrent - monocular). **(H).** Heatmaps of post-saccadic eye-endpoint densities for concurrent and monocular saccades (single animal), expressed relative to the pre-saccadic eye position (0,0) and plotted in degrees on the unrotated camera axes. **(I)** Polar distributions of saccade directions per eye and animal, rotated to align the eye’s dominant axis of motion to the 0/180 degrees axis (light gray circle = 0.005 density). **(J)** Axial bias [A.U.] of saccade directions comparing the axial bias of concurrent (x-axis) and monocular (y-axis) saccades (animals color-coded). For each dataset: bias = (P(main-axis) − P(perpendicular)) / (P(main-axis) + P(perpendicular)). The data underlying the graphs can be found in S1 Data.

Our recordings revealed that freely behaving *P. vitticeps* express a diverse oculomotor repertoire, with movements varying in speed, conjugacy, and temporal density over the course of each session (Fig 2B). Across animals, gaze vectors covered an average range of 35.0° ± 7.3° along the main movement axis (defined as the orientation of maximal bidirectional saccadic density) and 26.1° ± 6.4° along the perpendicular axis (S2 Fig). These spans were measured relative to each eye’s resting orientation, which varied modestly between recordings (σ_main-axis_ = 4.8°, σ_perpendicular_ = 2.9°). Within this workspace, gaze redirections often occurred as brief, discrete high-speed events, as next analyzed.

Saccades are rapid eye movements that abruptly shift gaze, minimizing transit time while maximizing accuracy [62,63]. To characterize the kinematics of saccadic eye movements in *P. vitticeps*, we identified saccades using an angular speed threshold of 0.8°/frame (~50°/s) applied to gaze-angle traces. This threshold was well above the empirically measured eye position tracking noise during verified no-eye movement epochs (SNR = 7.08; S10 Fig; Materials and methods). We detected multiple binocular configurations, including conjugate, divergent, convergent, disjunct (unequal inter-ocular dynamics), and monocular events (Fig 2B). Saccades exhibited stereotyped speed profiles characterized by rapid acceleration to a single peak, followed by smooth deceleration back to baseline (Fig 2C). The corresponding displacement showed a stereotyped sigmoid-shaped transition from the pre-movement baseline to a new gaze position, with most of the change occurring over a brief interval, and with a shallower tail as the movement approached its endpoint (Fig 2D). Across animals, peak angular speed scaled systematically with saccade amplitude, forming a linear relationship across most of the measured range, with a tendency toward saturation at higher amplitudes (>20°) (Fig 2E). The relationship was similar for monocular and concurrent saccades (S13 Fig), and to the one we observed in mice (S8B–S8D Fig).

In lateral-eyed rodents such as mice, eye movements during free behavior are tightly coupled with head-led gaze shifts, supporting faster transitions and providing image stabilization [36]. In such an acquisition scheme, the two eyes rotate in temporal coordination, with no monocular saccades. To quantify inter-ocular coordination in *P. vitticeps*, we distinguished between concurrent saccades (binocularly synchronous, but not necessarily directionally conjugated), in which both eyes crossed the speed threshold within a narrow temporal window (<34 ms), and monocular saccades, detected in only one eye. We found monocular (highly asymmetric) saccades in all animals and across all recording sessions (Figs 2F, S3, and S2 Video). Only 3.7% of monocular saccades were followed by a directionally aligned contralateral saccade within 80 ms, indicating that the vast majority were not subsequently corrected by the other eye. We next compared the independently normalized amplitude distributions of monocular and concurrent saccades. While small-amplitude saccades were more frequent than large-amplitude events in both distributions, monocular saccades were typically smaller than concurrent events (Fig 2G). Thus, monocular saccades are fast and local while concurrent saccades are larger, possibly reflecting gaze shifts during locomotion or broader visual sampling rather than local detail extraction.

To support the interpretation that some saccades reflect active gaze redirection rather than purely vestibulo-ocular stabilization, we asked whether they are observed when the head is still. To do so, we recorded head kinematics using a head-mounted accelerometer and identified movement and no-movement epochs (Materials and methods). When head movements were coupled to saccades, they tended to precede saccade onset (median: 78.1 ms). Both head-leading (59.4%) and saccade-leading (36.3%) sequences were common (with only 4.3% of events of simultaneous |dt| ≤ 17 ms saccades and head movement onset). While most saccades occurred during periods of head movement, a considerable fraction were detected during head-stationary periods (33.2 ± 9.4%, mean ± SEM; *n* = 15,613 events, 4 animals). Furthermore, monocular saccades constituted almost half of all head stationary events (48.9 ± 8.8%), whereas head-led saccadic events were less frequently monocular (21.5 ± 5.5%). Correspondingly, there was a small but significant correlation between saccade amplitude and movement (Monte–Carlo shuffle (*n* = 10,000): Δ = 0.64° (*n* = 4), p = 1 × 10⁻⁴). Post-saccadic back-and-forth sequences were also observed following 21.3% of large (≥7°) saccades during head movement (639/2997 events; S12 Fig). Thus, while most gaze shifts occur during movement and play a role in gaze stabilization, a considerable fraction of eye-led monocular (and binocular) gaze shifts occur during stillness, demonstrating that these movements are not exclusively coupled to head movement or gaze stabilization.

During free exploration, lizards frequently traversed the arena and produced repeated gaze shifts as they moved. Because directionality biases in eye movements often mirror ecological demands on visual sampling [2], we asked whether saccades exhibit structured directional organization. To test this, we expressed each event as a displacement vector in eye-centered coordinates and aligned all saccades to a common origin. We found that for concurrent saccades, relative endpoints clustered strongly along a single dominant axis, revealing a pronounced axial organization of gaze displacements. We note that these axes are tilted because cameras are aligned to the head-to-tail axis, while animals usually hold their heads at an inclined angle to this axis. We therefore rotated distributions to align to the main movement axis, defined as the axis of the maximal number of saccade events for each eye. This revealed that a bias towards a specific axis existed for concurrent saccades across animals (Fig 2I and 2J). In contrast, monocular saccades exhibited a broader and more isotropic distribution of endpoints (Fig 2H and 2J). In summary, we find a continuum of saccade profiles that shift between two separate modes of visual acquisition. During animal movement we find large, concurrent eye movements with a clear directional bias which can partially be explained by gaze stabilization. During stillness, when gaze stabilization is not active, we find smaller, isotropic eye movements that shift focus to small sub regions in the visual environment.

### Behavioral state affects ocular temporal dynamics and pupil dilation in *P. vitticeps*

In endotherms, spontaneous eye movements during wakefulness occur at a relatively stable rate of ~ 3–4 Hz [2,62]. As ectotherms, reptiles may employ a different temporal visual acquisition strategy shaped by their metabolic constraints and energy conservation demands [64]. To investigate the temporal dynamics of visual acquisition, we monitored eye and head movements for extended time periods (2–3 hours). We observed that eye movements shifted between a state with a limited number of eye movements (Fig 3A) and a state with a high frequency of eye movements (Fig 3B). To examine if these visual acquisition states coincided with animal movements, we segmented the behavioral recordings into Active states (during locomotion and head movements) and Quiet epochs (periods of stillness) using concurrent accelerometer measurements (Materials and methods). Active states constituted 10.2% of the total analyzed recording time. These epochs were accompanied by frequent eye movements (3–5 Hz; Fig 3B and 3C). In contrast, during Quiet states saccade frequency dropped under 1 Hz (Fig 3A and 3C). This effect was significant (Monte–Carlo shuffle (*n* = 10,000): Δrate = 2.66 Hz, *p* = 1 × 10⁻^4^). Notably, prolonged quiescent periods, devoid of any detectable eye movements, lasting up to several tens of seconds, were common (Fig 3A and 3F), suggesting discrete shifts between exploratory and stationary visual states. Separating inter-saccade interval (ISI) distributions by behavioral state confirmed that these long intervals were associated predominantly with Quiet periods: while the short-ISI distributions were broadly similar between states, Quiet periods showed a substantially greater prevalence of multi-second inter-saccadic intervals (S11A Fig). While these long intervals were widely distributed, an increased probability of 1–3 s intervals was observed across animals, suggesting a typical time scale during Quiet states (Fig 3F). Thus, *P. vitticeps* exhibits a transition between active and quiet behavioral modes reflected in both movement and visual acquisition.

**Fig 3:**
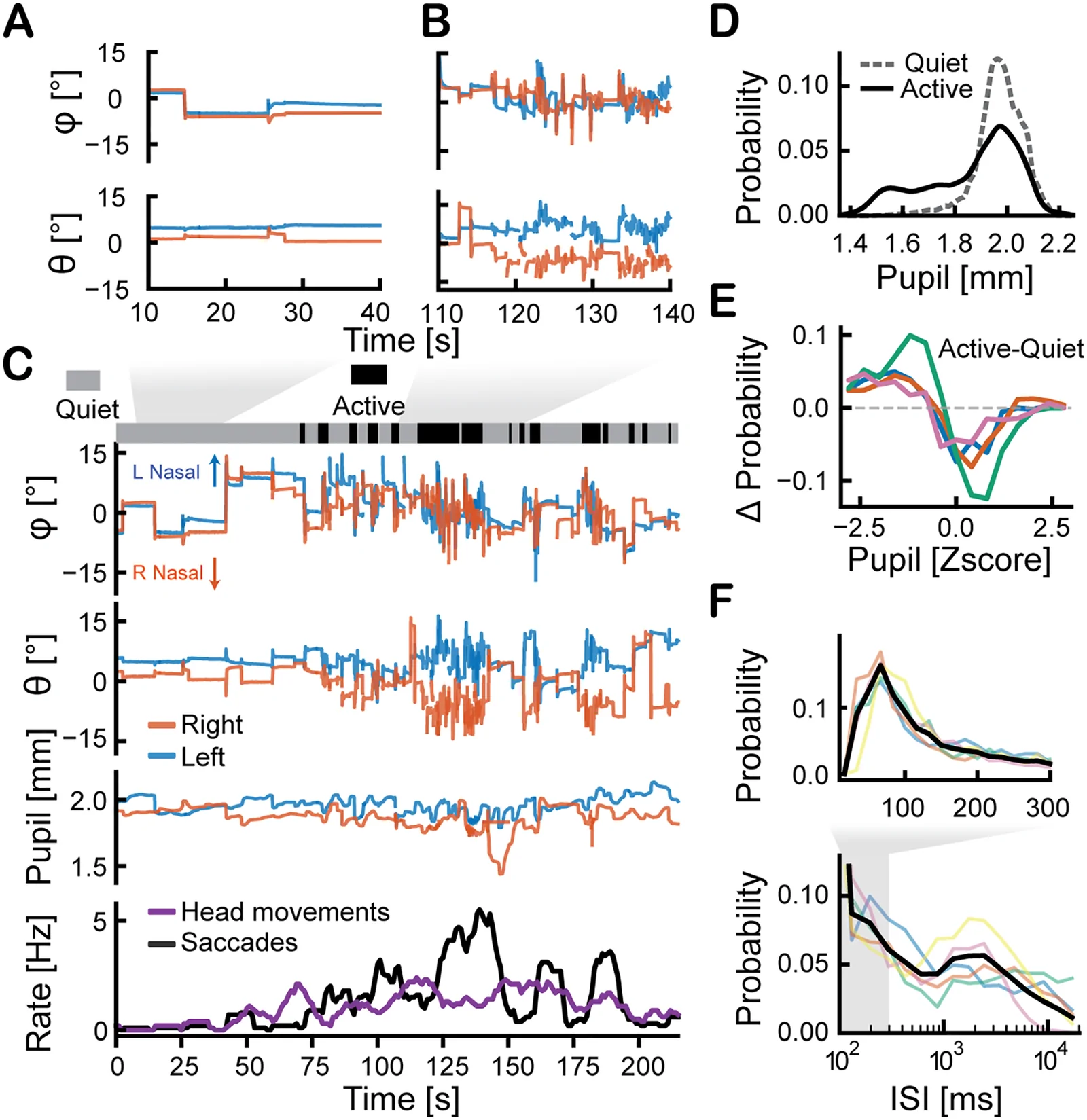
Visual acquisition profiles change with behavioral state and pupil diameter. **(A)** Eye angle dynamics for 30 s during a behaviorally quiet epoch. Angular traces were median-centered to each eye’s resting position. **(B)** As in (A) but for an active period. Note frequent saccades. **(C)** Eye angles, pupil size, and rate of saccade and head movement events acquired during free behavior. The top bar indicates behavioral classification into active (dark) and quiet (light) states with typical epochs enlarged in (A) and (B). Head movements (purple) and binocular saccade rates (black) were computed in sliding 10 s windows (1-s step). **(D)** Pupil diameter probability distribution for active (solid) and quiet (dashed) behavioral states for one animal (both eyes). **(E)** The difference between z-scored (mean/std) pupil-diameter distributions in active and quiet epochs. Positive deflections indicate pupil sizes that occur more frequently during Active periods. **(F)** Inter-saccade interval (ISI) distributions for combined Active and Quiet states (all data). ISI probability histograms shown on linear (top) and logarithmic (bottom) scales, with overlapping domains between 200 and 400 ms. Each trace represents one animal with the average over animals in black. The data underlying the graphs can be found in S1 Data.

The changes in behavioral state prompted us to examine the dynamics of pupil diameter. In mammals, pupil diameter is often binocularly coupled, and scales positively with arousal, providing an external index for cognitive effort and attention [65–68]. To discern inter-ocular pupil behavior in *P. vitticeps*, we computed left-vs-right pupil correlation values (Materials and methods). Pupils showed clear positive correlations during 66.3 ± 7.9% of the time (Pearson r > 0.2; Figs 3C and S4). Whereas pupils were uncorrelated for 23.4 ± 3.5% of the time (−0.2 ≤ Pearson r ≤ 0.2) and anti-correlated for 10.3 ± 4.9% (Pearson r < −0.2). Despite this divergence and given the transitions in behavioral states (Fig 3A – 3C), we wanted to understand if overall pupil diameter adheres to some relationship with arousal. To test state-dependent modulation, we compared pupil diameter distributions across behavioral states over long recording sessions (9.2 hours over 5 animals; Figs 3D and 3E). The long recording periods and uniformly illuminated arena were instrumental at averaging out illumination-specific effects. We found a significant difference (Monte–Carlo shuffle, *n* = 20,000 *p* = 5.0e−05) between states: pupils were smaller during Active epochs and dilated during Quiet epochs (Fig 3D). This difference was consistent over animals (Fig 3E). Interestingly, it is inverse in directionality to the one found in mammals.

## Discussion

We developed a versatile and technically accessible approach for eye-tracking in freely moving small animals. By combining 3D photogrammetry with personalized 3D-printed headmounts, we established a framework that is rapidly adaptable across species, as demonstrated across three species with distinct cranial morphologies (Fig 1). Focusing on *P. vitticeps*, we found a rich oculomotor repertoire comprising gaze stabilizing saccades during movement epochs with axial biases, and exploratory monocular and binocular saccades during stationary epochs (Fig 2). Finally, we found that saccades dynamically changed throughout long recordings, exposing a bi-phasic visual acquisition strategy that was tied to behavioral state and pupil diameter fluctuations (Fig 3). The implications of these results on our understanding of visual acquisition strategies across species are discussed below.

Visual strategies across species depend on the degree of head motility, the position of eyes, and the morphology of retinas, and are reflected in saccade kinematics and oculomotor movement statistics [69]. We found that eye movement spans are anisotropic with a clear bias to larger movements in one orientation (Fig 2H–2J). Comparable directional anisotropies have been reported in other amniotes, including primates, rodents, chameleons and birds [29,53,70]. Such anisotropy likely reflects a combination of biomechanical constraints of the oculomotor plant [71,72] and the nonuniform distribution of behaviorally relevant visual targets across the sampled environments [73], or the effect of the locomotor and postural movement range of the animal. Stabilization of head roll and pitch during locomotion, while yaw rotations occur during orienting turns, may favor an evolutionary overrepresentation of horizontal eye movements [74,75]. Another property with striking similarity to visually oriented amniotes is saccade kinematics [2,62]. Individual eye movements in *P. vitticeps* showed the characteristic rapid acceleration and deceleration phases that define vertebrate saccades [38,76] (Fig 2C and 2D), and their peak speed scaled linearly with amplitude (Fig 2E). We observed the same regime in our mouse recordings (S8B–S8D Fig). This relationship largely resembles the ‘main sequence’ previously described in primates, cats, and mice [77–80], with a similar rate slope of ~20 [1/s] in both primates [79] and *P. vitticeps* (Fig 2E). Interestingly, the main sequence curve differed at high saccade amplitudes for which primates exhibit strong saturation in contrast to only modest saturation in *P. vitticeps*. This could result from factors such as eye geometry. However, the similarity in the saturation value for both primates and *P. vitticeps* (~15°) suggests that this discrepancy results from the larger oculomotor span (90° [2,81] in humans versus 35° in *P. vitticeps*, S5 Fig). Indeed, the absence of individual eye movements larger than 25° in *P. vitticeps* likely reflects the animal’s reliance on head movement contributions during broader gaze shifts, confining saccades to the low-amplitude regime where peak speed is not yet saturated [63]. In that sense, the nonsaturated profiles in *P. vitticeps*, and the reliance on head movements (~40% of gaze shifts were executed when the head was stationary, whereas the remaining ~60% occurred during coordinated eye-head saccades) are advantageous relative to the saturated profiles in humans. This may reflect a limited ability to move large heads fast enough. Alternatively, the estimates of the natural span in humans (and the consequent speed saturation with amplitude) may be biased by the head-restraint conditions used in many experiments [77,79,81]. This is consistent with the lack of large ocular excursions during naturalistic, head-free behavior [79,82,83].

Across taxa, saccades can serve different functional roles, ranging from compensatory quick phases that accompany head movements to voluntary shifts that reposition the eye toward behaviorally relevant targets [2]. In many lateral-eyed mammals such as rodents and ungulates, saccades are reported primarily as quick phases supporting head-led gaze shifts and vestibular correction [36,38]. Such animals rely predominantly on head rotations to redirect gaze with minor oculomotor span (~20° for mice [36]). This behavior fits the lack of a clear localized fovea or the existence of a visual streak [84–86]. Similarly, we observed nystagmus-like post-saccadic back-and-forth movements in a subset of large saccades occurring during head movements, consistent with a compensatory role in gaze stabilization during head movement (S12 Fig). By contrast, *P. vitticeps* frequently generated saccades in the absence of head movements, consistent with eye-led reorienting similarly to primates. These events likely reflect visually guided ocular shifts, and are supported by evidence for a fovea in agamid lizards [47]. We note, however, that foveated animals can perform visually guided behaviors also using head movements. Avian reptiles can quickly direct gaze using their heads, with some species exhibiting only minimal eye movements (~3° for barn owls [87,88] and ~5° in chickadees [89]) while relying on head movements for both saccades and image stabilization during locomotion [3,90,91]. In contrast, while using head movements similar to those of head-orienting birds, the propensity for eye-only saccades in *P. vitticeps* is reminiscent of some acquisition strategies in foveated primates. In primates, the existence of directed, time-stamped saccades has been widely leveraged to study attention and decision-making [5,92]. The presence of eye-led gaze shifts in *P. vitticeps* suggests a tractable route for extending visual attention studies to reptiles, thus providing a comparative perspective that can shed light on the evolution of attention and corresponding neural processing [93].

In laterally eyed species, monocular saccades can be advantageous because portions of the visual field are accessible to only one eye, making unilateral shifts a direct and efficient means of sampling the most relevant features of each hemifield [48,94]. However, this potential is not uniformly expressed across taxa: many lateral-eyed mammals and birds exhibit tightly yoked ocular rotations [29,36]. Our analysis of mouse eye movements agrees with this pattern, with saccades exhibiting coupled ocular dynamics (S8E Fig). Against this backdrop, we observed flexible ocular coupling in *P. vitticeps* (Fig 2A, 2F and 2G). Asymmetric and monocular saccades were common, forming a graded continuum of coupling strengths (Fig 2F). This regime is consistent with proposals that monocular and conjugate saccades may arise from the same control architecture, with coupling strength being a tunable parameter [94]. In this view, monocular independence and binocular conjugacy represent endpoints of a shared control space, with coordination varying by context. Chameleons, for example, exhibit extreme ocular independence to support crypsis during scanning, yet transiently increase coupling for prey capture [52,95]. By contrast, mammals with front-facing eyes, including primates and several predatory rodents, exhibit reinforced binocular coupling that favors tightly yoked rotations for stereopsis and coordinated pursuit [33,96]. *P. vitticeps* exemplifies a balanced regime in which modest monocular independence coexists with reliable binocular coordination.

The temporal structure of eye movements in *P. vitticeps* revealed a striking departure from patterns described in endothermic vertebrates. Lizards often remained without ocular motion for several tens of seconds, intervals far longer than the continuous high-frequency sampling typical of endothermic vertebrates, including birds [97], rodents [36,80], and primates [16,62,98,99]. In *P. vitticeps*, these prolonged quiet states alternated with relatively brief epochs of dense saccadic activity (active states), producing a bimodal distribution of inter-saccadic intervals (Fig 3). Whether this temporal organization represents a broader reptilian or ectothermic visual acquisition strategy will require comparative measurements across additional species. This behavior aligns with the well-documented postural immobility in ectotherms [100], but the accompanying stillness of the eyes appears distinctive and indicates a state in which visual sampling demands differ markedly from those that drive active scanning [101]. How the brain uses these quiet periods to process information remains to be studied [102,103].

In mammals, behavioral states are linked with brain states and pupil diameter across both sleep and wakefulness [36,65–68,80,104]. We found pupil diameter in *P. vitticeps* constricted during active, movement-rich states and dilated during quiet wakefulness. This polarity contrasts with the dilation-with-arousal pattern characteristic of mammals [66–68]. A similar inversion has recently been documented in birds, where pupil constriction accompanies aroused wakefulness and REM-like sleep [105]. Because both reptiles and birds possess predominantly striated iris musculature [106], this cross-taxon correspondence suggests that rapid, behavior-linked pupil adjustments may reflect a broader sauropsid pattern rather than a feature unique to birds. Since the functional basis for pupillary state-dependence remains unclear, the existence of highly structured sleep state transitions in *P. vitticeps* [107] could provide valuable clues for its function.

Taken together, our findings highlight the value of comparative head-free eye-tracking approaches. Recent technological advances provide unprecedented access to structural and functional properties of eyes and retinas on both the cellular and molecular level and across species [69,108–113]. Our work complements these experimental advances by enabling precise quantification of ocular behavior in species that have been largely inaccessible using conventional eye-tracking techniques. Going forward, the compatibility of our method with electrophysiology and multi-camera tracking provides a practical route for extending our understanding of visual neural processing across taxa. Our results demonstrate how expanding eye-tracking to reptiles can facilitate the extraction of conserved acquisition strategies (e.g., the saccadic main sequence and axial biases in scanning) as well as specializations (e.g., *P. vitticeps’s* acquisition mode and modulation of pupil diameter by behavioral state). Such a multi-perspective view on natural visual acquisition across species will be essential for developing an understanding of vision, its ecological functions, and its evolutionary trajectory.

## Materials and methods

### Animals

Eye movements were recorded from five dragon lizards (*Pogona vitticeps)*, two red-eared slider turtles (*Trachemys scripta elegans)*, and one mouse (*Mus musculus)*. Lizards were obtained from commercial suppliers and housed in a dedicated animal facility at the I. Meier Segals Garden for Zoological Research at Tel Aviv University, under a 12:12 hour light-dark cycle (07:00–19:00) at 24 °C. Lizards were fed mealworms (*Tenebrio molitor*), cockroaches (*Nauphoeta cinerea*), and vegetables, with water available ad libitum. Red-eared slider turtles were maintained in an enclosed outdoor pond at the zoological garden of Tel Aviv University (Approval number: H10900/2024). Before experiments, turtles weighing between 800 and 2000 grams were transferred to an indoor laboratory tank (100 × 120 × 74 cm; W × L × H) equipped with a basking platform and heat and light sources, under a 12:12 hour light-dark cycle (07:00–19:00) at 24 °C. Male and female mice (C57BL/6) were used. Animals were housed under standard vivarium conditions (22 °C, 12-h light/dark cycle, with ad libitum food and water). All experimental protocols complied with relevant ethical regulations for animal use and were approved by the institutional animal care and use committee at Tel Aviv University (Approval numbers: TAU-LS-IL-2410-145-4, TAU-LS-IL-2411-147-4).

### Eye-tracking system

Eye movements were recorded using a custom dual-camera IR eye-tracking system. Two miniature IR-sensitive cameras (NoIR Spy Camera for Raspberry Pi, Arducam OV5647 sensor) each controlled by an independent Raspberry Pi 4B. Cameras were aimed at the eyes via small (8 x 8 mm) 940 nm IR-reflective mirrors connected to a personalized, single-piece 3D-printed headmount. The headmount was designed from a photogrammetry-derived 3D model of each animal to define precise camera-eye geometry and ensure unobstructed visibility of both pupils. Head shape itself primarily imposes species-specific design constraints on camera and mirror placement, which can be easily accommodated by the customizable geometry of the system. A stable headmount attachment point is a required prerequisite but should be possible for animals with a skull. Animals with a larger distance between the eyes and the headmount will require longer mirror holders, which if too long may result in vibrations and require thicker holders. Successful tracking requires sufficient pupil contrast. Although we did not quantify a minimum contrast threshold or explicit skull thickness thresholds, the system was successfully installed and used in mice, which had the thinnest skull and least distinct pupil boundary among the species tested here. Mirrors were held in separately printed holders, allowing rapid replacement of this delicate component without modifying the rest of the headmount. Each camera was fitted with a QILENS 3.7 mm manual-focus M7 lens to account for the short working distances (~ 1.8–4 cm) and a custom-cut IR-pass filter (high-pass > 930 nm). Illumination was provided by two 940 nm IR LEDs per camera, wired through a Cyclops LED driver for precise control and synchronization verification via predefined LED-off events (see Data synchronization below). To allow rapid, repeatable connection across sessions, the headmount is attached to a chronically implanted hexagonal standoff (brass M3 (0.6 g) for lizards and turtles, plastic M2 (0.15 g) for mice), and the underside of each headmount has a matching hexagonal recess. This keyed interface constrained rotation and ensured that the headmount always returned to the same orientation relative to the eyes. Rarely, animals collided with the arena boundaries, breaking the mirror holders extending from the headmount. We therefore integrated generic holders that interlock to the headmount, restoring the predefined geometry upon replacement.

### Anchor implantation surgery

To provide a stable connection point for the headmount, animals were implanted with a lightweight cranial anchor. Surgeries were performed under isoflurane anesthesia (4%) with perioperative analgesia (meloxicam 0.2 mg/kg or carprofen 2 mg/kg) and prophylactic antibiotics (enrofloxacin 5 mg/kg). Following exposure and cleaning of the skull, the hexagonal anchor (brass M3 or plastic M2) was affixed using UV-curing adhesive and dental cement. The possibility to implant screws in the skull is animal-specific. In reptiles and turtles, the anchor was reinforced using miniature cranial screws inserted to a depth of 0.4–0.5 mm. Since in some animals the skull may not be thick enough to install screws, the anchor can be secured using adhesive and dental cement alone, as we demonstrated for the mouse. Ectothermic animals (*P. vitticeps*, *T. scripta elegans*) typically resumed normal activity within ~12 hours, whereas mice recovered after ~2 hours. Photogrammetry scans for headmount design were performed under anesthesia once the anchor had fully stabilized (after cement curing, typically within minutes). A detailed surgical protocol is provided in S1 Text. The implantation procedure and anchor configurations used across species are illustrated in S7 Fig.

### Turtle-specific adaptations

Because turtles retract their heads during unconstrained behavior, we set the anchor point for the system on an extender module between the cranial anchor and the headmount attachment point (S7C5 Fig). The module length (50 mm) was calibrated to allow the animal full head retraction of the head without causing a collision between the carapace and headmount. To provide a connection point for the extender module, an M2 hex nut was connected to the module and embedded in dental cement, creating a detent that enabled a reliable 3D model and precise repositioning between sessions (S7C4 Fig). An early version of the system was used for data collection in Becker and colleagues [56].

### Photogrammetry-based animal modeling

High-resolution 3D models of each animal’s head were generated to enable precise virtual fitting of the personalized headmount. While animals were under anesthesia, 40–80 photographs were captured from multiple viewpoints using a Sony RX100-II camera under diffuse room lighting, ensuring complete coverage of the head-anchor-eye geometry. Images were processed with the open-source photogrammetry pipeline Meshroom (AliceVision), producing 3D textured mesh of the scene. Scale was set using the known physical dimensions of the implanted standoff, which provided a rigid and easily identifiable reference for the model. Calibrated meshes were inspected for reconstruction artifacts and, when necessary, lightly cleaned (e.g., removal of floating surfaces and background) before being imported into CAD software (Autodesk Fusion) for headmount design and virtual camera alignment. We did not encounter difficulties due to differences in overlying skin or muscle in the species we tested. The broad application of photogrammetry to diverse subjects suggests that this is not an inherent limitation of the technique itself [114]. However, it is critical that both the implanted hexagonal screw and the eyes are visible in the photogrammetry scan. Full photogrammetry settings and parameters for Meshroom are provided as a project file in the associated repository.

### Headmount virtual fitting and fabrication

Personalized headmounts were designed in Autodesk Fusion using the scaled photogrammetry-derived meshes as geometric reference. Rather than sculpting full headmounts de novo, we assembled each design from a library of standardized functional modules (camera holders, mirror holders, and anchor interfaces) that were positioned around the 3D head model to define the desired camera-eye geometry. These modules were then joined into a single rigid piece using simple lofts and connectors, yielding a reproducible headmount that preserved the designed spatial relationships. An interactive fitting environment for Autodesk Fusion and a step-by-step guide are available in the project repository. A hexagonal recess matching the implanted standoff provided a keyed, repeatable mounting orientation across sessions. After printing, IR mirrors were inserted into their dedicated sockets, and IR LEDs were wired into the built-in channels and secured with adhesive, then coated with a thin layer of epoxy resin. Comprehensive virtual fitting guide, printing specifications, and project files are available in S1 Text and the associated repository.

### Alternative modular headmount solution

In rapidly recovering animals (e.g., mice), normal behavior is resumed before a custom single-piece headmount can be printed (which may require >10 hours). To bridge this gap, we used a modular headmount kit consisting of interlocking, ball-jointed camera holders connected to the slotted anchor by adjustable support arms (Fig 1D). Support arms spanning a range of lengths and angles were printed in advance. Following the photogrammetry session, fitting was performed under anesthesia: the modular headmount was assembled on the hexagonal anchor, and cameras were installed on the support arms and aimed at the eyes using live video feedback under IR illumination. Once alignment was achieved, ball-joint positions were fixed with UV-curing adhesive. The headmount was then removed for wiring and epoxy coating, as in the single-piece design.

### Video acquisition camera specifications and optics

Eye videos were acquired using miniature IR-sensitive Arducam B0066 “NoIR” board cameras equipped with OV5647 1/4-inch, 5-MP sensors and mounted on the custom headmount. To enable sharp imaging at short working distances, the stock optics were replaced with manual-focus lenses. For lizards and turtles, cameras were fitted with 3.7-mm f/2.55 M7 pinhole lenses; for mice, 4.5-mm lenses with a narrower field of view were used. Custom IR-pass filters (>930 nm) were fitted to the lenses to minimize visible-light contamination. Cameras viewed the eyes indirectly via 940 nm reflective mirrors that remained visually transparent to the animals, allowing close-range imaging while keeping the cameras outside the direct field of view. Two 940 nm IR LEDs mounted adjacent to each camera provided illumination.

Camera configuration and video acquisition were controlled using custom Python code running on each Raspberry Pi via SSH. Videos were acquired at 640 × 480 pixels and 60 frames/s, with working distances of ~1.5–5 cm. A digital frame-acquisition event was generated for every captured frame and transmitted to the Open Ephys acquisition system for temporal alignment across data streams. The IR LEDs were powered by a Cyclops LED driver, which was programmed to suspend illumination for 32 ms at the end of each minute while transmitting an LED-ON event to the Open Ephys acquisition board. These programmed illumination interruptions provided an additional offline synchronization marker between the eye videos, accelerometer data, and external behavioral recordings. Recording sessions, typically lasting 3–4 h, were divided into 30-min blocks.

### Video stability assessment

Camera stability was quantified by tracking frame-to-frame shifts of a fixed region of interest (ROI) in the eye video. For each block and eye, a static ROI was manually selected on a stable feature of the image (headmount feature when available or stable mirror edges or prominent stationary scales in *P. vitticeps*). The ROI was extracted for all frames, and a normalized 2-D cross-correlation was computed between the ROI of the current frame and that of the first frame in the sequence. The location of the correlation peak was taken as the best alignment between frames, and its displacement relative to the reference peak (frame 0) was recorded for each frame. Per-frame jitter was computed as the Euclidean distance of this displacement, which was subsequently converted to physical units using an individually calibrated pixel-to-millimeter scale derived from anatomical landmarks. These displacements were aggregated across stable periods to yield a per-camera jitter distribution. To compare mechanical stability across headmount configurations, this analysis was applied to recordings obtained using modular headmounts in mice, turtles, and lizards, as well as personalized single-piece headmounts in lizards. We found that in all configurations the median jitter amplitude was ≤ 90 µm—typically 1–2-pixels. Within lizards, the single-piece headmounts were characterized by smaller jitter (S9 Fig), suggesting it is the optimal solution when applicable.

### Behavioral arena and external video acquisition

Experiments were conducted in a custom behavioral arena (70 x 100 x 45 cm) equipped with four overhead and side-view video cameras providing synchronized coverage of the full arena. External behavioral videos were recorded continuously at 60 frames-per-second. For recordings involving head-movement analysis, animals also carried an Intan RHD-2132 headstage, and accelerometer measurements from the onboard sensor were acquired directly by the Open Ephys system. External video streams and accelerometer signals were used to annotate behavioral state (active versus quiet) and to verify the correspondence between eye movements and whole-body activity. Mice were placed within their home cage inside the arena for recordings.

### Data synchronization

Digital frame-acquisition signals from the left and right eye cameras, LED-state signals from the Cyclops driver, and external video events were recorded concurrently using an Open Ephys acquisition board. For each recording block, these digital event logs were parsed to construct an initial mapping between Open Ephys timestamps and eye-camera frame indices, external video frames, and accelerometer samples (already recorded directly in Open Ephys). Synchronization was visually validated using the 32-ms LED-off events programmed into the Cyclops driver. All LED-off events were automatically detected across modalities and compared. When discrepancies occurred, a small user-applied temporal correction was made via a Python interface. After verification, a per-block synchronization table was generated containing frame-level correspondences between eye videos, external behavioral videos, and accelerometer data. All analyses used these synchronized time bases (S6 Fig and S1 Video).

### Video analysis and eye angle reconstruction

Pupil positions were first estimated frame-by-frame using DeepLabCut, and frames with low-confidence detections were excluded. For each valid frame, a least-squares ellipse was fitted to the pupil boundary, yielding its center and major/minor axes. Angular eye position was then computed using a geometric method derived from Kerr and colleagues (2013), which infers azimuth (φ) and elevation (θ) from two measurable image quantities: the pupil-ellipse eccentricity (minor/major axis ratio) and the pupil-center displacement relative to the condition where the eye is pointing directly along the camera axis. In this framework, changes in ellipse eccentricity determine a depth-like scaling factor that relates pupil-center offsets in the image to rotations of the eye, and φ and θ are obtained by converting these normalized offsets into angular displacements using inverse-trigonometric relationships [25]. Because animals rarely look exactly at the camera, the reference position required for this computation was determined geometrically. The two IR LEDs mounted symmetrically adjacent to the camera produce reliable reflections on the cornea. We use them to define the camera-axis reference position. By annotating frames in which both reflections were visible, we identified the pupil location corresponding to the camera axis for each eye and used this as the zero-angle reference for subsequent calculation of the eye vector relative to the camera as described above. To quantify the accuracy of this reconstruction procedure under our optical geometry, we evaluated it using a Blender-generated synthetic dataset in which a 3D eye was rotated systematically through known azimuth-elevation combinations (−60° to +60°) (Blender file available in the project repository). Applying the full ellipse-fitting and projection pipeline to the rendered images yielded angular errors that increased with eccentricity, ranging from ~1 near the center to ~5 at the maximal eccentricities reached by *P. vitticeps* during behavior when taking camera tilt into account (S1A Fig). Before plotting, φ and θ traces were recentered from the camera-centric (0,0) alignment to anatomically comparable resting positions by subtracting their block-wise medians. Because subsequent analyses quantified eye movements relative to each eye’s resting position, we additionally assessed the effect of reprojection error on relative angular measurements. Raw and corrected eye-position estimates were independently zeroed to their respective resting positions, and the residual difference between these rest-centered datasets was used to quantify relative reprojection error (S1B Fig). Pupil diameter (mm) was computed from the ellipse major axis using the same pixel calibration value used for camera jitter quantification.

### Spatiotemporal limitations

The temporal resolution of eye movement measurements was limited by the 60-Hz camera frame rate (~16.7 ms between frames). Consequently, velocities represent interframe angular displacements and do not resolve sub-frame velocity profiles; movements that occur and return near their initial position within a single interframe interval cannot be detected nor can changes in velocity occurring at time scales faster than the inter frame interval. Spatial resolution is limited by pixel size, magnification, and spatial noise from pupil ellipse fitting.

The combined tracking noise was estimated empirically from frame-to-frame angular gaze displacement during stationary-eye periods (S10 Fig). For each consecutive frame pair, displacement was computed as D = √(Δφ^2^ + Δθ^2^) [°/frame]. Under the assumption that the per-axis angular increments (Δφ, Δθ) are independent and Gaussian with equal standard deviation, D follows a Rayleigh distribution. To corroborate this, we compared each quiet-window sample to an analytical Rayleigh distribution with scale matched to the sample median, using the Kolmogorov–Smirnov statistic DKS (largest gap between empirical and model CDFs; S10 Fig). Maximum gaps were small (lizard DKS = 0.057, mouse DKS = 0.071, turtle DKS = 0.106). We took the sample median of D as the typical noise magnitude. Detection signal-to-noise ratio (SNR) was defined as the saccade-detection threshold divided by this median. For the lizard data in S10 Fig (5,640 frame-to-frame steps from five stationary segments, both eyes), the noise value for the interframe eye displacement was: median(D) = 0.11°/frame (~6°/s). While it is possible to extract eye movements at lower speeds, we set a conservative detection threshold of 0.8°/frame (~50°/s) that is considerably higher than the noise (SNR = 0.8 / 0.113 = 7.08). For mice, median(D) = 0.31°/frame and the detection threshold was 3.23°/frame, corresponding to SNR = 3.23 / 0.31 = 10.59.

### Head movement detection and behavioral state segmentation

Where mentioned, head-movement activity was quantified from the three-axis accelerometer embedded in the Intan RHD2132 headstage, which was attached to the animal’s head via an implanted connector. Raw acceleration traces were processed using custom MATLAB code to identify movement events by first estimating the noise distribution from low-variance segments of the filtered signal (selected using kurtosis-based criteria) and then classifying timepoints as movement when the acceleration magnitude exceeds the noise baseline by several standard deviations. The resulting time-stamped movement events were imported into Python and used to annotate saccadic events (see below). Movement events were also converted into a continuous behavioral annotation by applying a rolling-window analysis (10-s window, 1-s step). For each window, the mean movement magnitude was computed, and windows were labeled as active when the mean exceeded a manually chosen threshold (visually chosen and verified by comparing accelerometer traces with arena videos of animal behavior) and quiet otherwise. Consecutive windows of matching labels were merged into one behavioral epoch. These annotations were used without temporal smoothing for all state-dependent analyses in Fig 3, thus epochs ≥1-s were included in the computations. To quantify the duration of sustained behavioral states (S11B Fig), we additionally generated a smoothed annotation in which opposite-state interruptions shorter than 3 s were bridged and resulting epochs shorter than 5 s were excluded. The median lengths of these smooth states were 15 s for Active epochs (IQR = 10–21 s) and 59 s for Quiet epochs (IQR = 32–170 s).

### Saccade detection and segmentation

Eye movement events were detected separately for each eye by computing frame-to-frame changes in reconstructed eye angles (φ, θ) and identifying periods in which the interframe angular speed exceeded a fixed threshold (48 °/s). Consecutive suprathreshold frames were grouped into candidate saccades. Because some events contained multiple direction changes, each candidate event was subdivided whenever the instantaneous movement direction shifted by more than 90°, and only segments with a minimum duration (2 frames) and a net angular displacement >0.5° were retained. For each qualified saccade segment, onset and offset times and net angular displacement were extracted. When head-movement information was available, each saccade was labeled as “with head movement” if its duration overlapped any accelerometer-defined movement event (as described above), and “without head movement” otherwise. Binocular classification was performed by matching left and right eye saccades based on their onset time. Pairs whose onsets differed by <34 ms were classified as synchronized binocular saccades, and all remaining events were treated as monocular. All subsequent analyses of saccade amplitude, direction, dynamics, and pupil behavior used these classified sets. For Fig 2F, the ipsilateral peak for each event was taken from the detected saccade, while the contralateral peak is the maximal instantaneous speed within ±51 ms of onset (irrespective of contralateral detection). Because onset-based pairing can undercount binocular saccades when temporal drift occurs between eye videos within the 1-min verified epochs, we implemented an additional step to define a conservative set of verified monocular events. For each nonsynchronized (candidate monocular) saccade detected in one eye (the “ipsilateral” eye), we examined the continuous ocular speed trace of the contralateral eye directly, irrespective of whether a contralateral saccade was detected by the segmentation algorithm. We computed the contralateral peak instantaneous angular speed within a symmetric temporal window around the ipsilateral onset (±51 ms, i.e., ±3 frames at 60 Hz). If the contralateral peak speed remained below the saccade threshold (0.8 °/frame, ≈50 °/s) throughout this window, the event was classified as verified. If contralateral peak speed exceeded the threshold, the event was tagged as unverified. Events for which contralateral traces were unavailable in the verification window were excluded from the verified monocular set (S2 Video). All reported “monocular” fractions in the analysis were computed using this verified subset. To complement the monocular head-static analysis in Figs 2F, S3 Fig shows a similar coupling for all head-static and head-moving saccades with increased resolution (100 x 100 bins versus 60 x 60 in Fig 2F). To test whether monocular saccades were followed by a corrective movement of the contralateral eye, we identified contralateral saccades occurring within 80 ms of monocular saccade onset and within 45° of its movement direction. Such corrective mono-binocular sequences were rare, occurring in only 3.7% of 15,070 monocular events. To compute Inter-saccade-interval histograms (Fig 3F), ISI values were collected within each recording and probabilities were calculated for each animal over the full ISI range (10–40,000 ms), then applied uniformly to both panels. For the post-saccadic analysis (S12 Fig), large saccades (≥7° net displacement) occurring during head movement were classified as back-and-forth sequences when they were followed within 150 ms by at least two additional smaller saccades, with >90° direction reversals between consecutive events and at least one event counter-directed relative to the primary saccade.

### Head movement and saccade amplitude

To test whether head movement was associated with saccade amplitude, saccades were pooled across blocks within each animal and split by a binary head-movement label. For each animal (*n* = 4 animals with accelerometer measurements), we computed the difference in mean amplitude between conditions, and summarized the effect as the mean Δamplitude across animals. Significance was assessed with a within-animal Monte–Carlo permutation test. Head-movement labels were randomly reassigned across saccades to generate a null distribution of mean Δamplitude values, from which a two-sided *p*-value was computed.

### Head movement and saccade onset

To quantify the timing of head movement relative to saccades, accelerometer movement samples were grouped into bouts, with consecutive samples separated by >40 ms assigned to separate bouts; head movement onset was defined as the first sample of each bout. Concurrent left- and right-eye saccades with onsets within 34 ms were treated as a single event, timed at the earlier onset. For each saccade, the nearest head movement onset within ±500 ms was identified and its signed latency relative to saccade onset was calculated (Δt = t_head − t_saccade; negative values indicate head-leading events). Events within one video frame (±17 ms) were classified as simultaneous. This analysis included 18 recording blocks from four animals for which accelerometer-derived movement annotations were available.

### Behavioral state and saccade rate

To test whether behavioral state predicted saccade rate, Active/Quiet annotations were converted into nonoverlapping 1-s time bins, and saccade onsets were counted per-bin. For each animal, rates were computed as total saccades divided by total annotated time in each state, yielding Δrate (rate_active - rate_quiet), and the population effect as the mean Δrate of this across animals. Significance was assessed by Monte–Carlo permutation of state labels across time bins within each animal (preserving the number of active/quiet bins) to generate a null distribution of mean Δrate values, from which a two-sided p-value was computed.

### Pupil data analysis

Pupil diameter (mm) was derived from the fitted ellipse parameters in each eye-tracking frame by converting the major axis from pixels using the per-eye pixel calibration described above. Frames with low DeepLabCut likelihood (<0.9), including periods in which the pupil was occluded by blinking or movement of the nictitating membrane, were excluded from subsequent eye movement analyses. To limit eye-position artifacts caused by movement of the camera assembly relative to the eye, samples exhibiting >5-pixels interframe image displacement were excluded from subsequent eye movement analysis. This criterion excluded 0.21% of samples across the dataset. To characterize state-dependent changes in pupil size, pupil traces were aligned to behavioral annotations, and distributions were computed separately for active and quiet periods. For each animal, pupil diameters from both eyes were combined, clipped to remove extreme outliers, and z-scored relative to the animal’s pooled distribution. State-dependent effects were quantified as the difference between the active and quiet z-scored histograms (Active - Quiet), allowing comparison across animals despite inter-individual differences in absolute pupil size. This reported, for each normalized diameter bin, whether that range is sampled more frequently during active or quiet behavior (Fig 3E). To assess whether these differences exceeded chance, we performed a permutation test separately for each animal by randomly shuffling the active/quiet labels within each block and eye while keeping the pupil time series fixed. For each shuffled dataset, we recomputed the difference in median pupil diameter between quiet and active epochs and used the resulting null distribution to obtain a two-sided *p*-value for the observed median difference. Inter-eye pupil coordination was quantified from the z-scored traces by computing rolling Pearson correlations between left and right pupil diameter in 10 s windows, requiring at least one third of samples per window. Each correlation value was labeled by behavioral state and classified as positively correlated (r > 0.2), weakly coupled (−0.2 ≤ r ≤ 0.2), or anti-correlated (r < −0.2). For each animal, the fraction of time spent in each category was calculated separately for all, active, and quiet epochs and visualized as stacked bar plots.

### Orientation and directional analysis

To characterize the directional structure of eye movements, each detected subsaccade was represented by its net angular displacement in azimuth-elevation space (Δφ, Δθ). For visualization, all saccades were translated so that their starting positions lay at the origin, and their endpoints were plotted in this common coordinate frame. This produced an egocentric map of saccade directions that is independent of the animal’s instantaneous eye position. For saccade target heatmaps (Fig 2H), densities were estimated with a 2D Gaussian kernel on a ±20° grid. For each animal, saccade events were histogrammed and a main movement axis was identified by scanning candidate orientations (0–180) and selecting the axis with maximum saccadic events (Fig 2I). To determine the movement bias for saccades along this axis, on-axis events were defined as within ±45° of the main movement axis. Monocular and binocular saccades were analyzed separately, and the bias for each animal was displayed for concurrent and monocular events (Fig 2J).

### Declaration of AI-assisted technologies use

Custom scripts for statistical analysis and data visualization were developed with the assistance of ChatGPT (OpenAI) and Cursor AI. All AI-assisted code was reviewed and validated by the authors through manual inspection, testing, and verification of the resulting outputs. No generative AI tools were used to create or modify graphical content in the manuscript figures. The authors take full responsibility for the accuracy and integrity of the reported results.

## Supporting information

S1 Text Supplementary methods. (PDF)

S1 Fig Validation of angular eye-position reconstruction. **(A)** Simulation-based assessment of angular reconstruction accuracy. A rendered 3D eye was rotated through known azimuth (φ) and elevation (θ) angles from −60° to +60° in 1° increments, and reconstructed eye positions were compared with the ground truth. Ellipse eccentricity (minor/major axis ratio; top) and mean total angular error, √[(Δφ)^2^ + (Δθ)^2^], as a function of gaze eccentricity (bottom) are shown. The dashed line indicates the extended *P. vitticeps* oculomotor range (±35°). **(B)** Eye-pooled relative reprojection error after independently centering the raw and corrected eye-position estimates on their respective resting positions. Residual absolute errors were φ: 0.84 ± 0.48° (range: 0.42–1.67°; mean ± SD across animals; *n* = 5) and θ: 1.19 ± 0.48° (range: 0.65–1.93°; *n* = 5).
(TIF)

S2 Fig Oculomotor span for each animal. For each animal and eye, saccade directions were used to estimate an initial movement axis by identifying the peak of the bidirectional (0–180° folded) saccade-direction histogram. Gaze coordinates (φ, θ; centered relative to each eye’s resting orientation) were rotated by the shortest angle needed to align this axis horizontally. The central 95% gaze span (2.5–97.5 percentiles) was computed along the rotated axes. If the axis perpendicular to the saccade-aligned axis showed a larger span, axes were swapped so that the dimension with the greater gaze range defined the main axis. Points show per-animal spans (mean across eyes). Color indicates animal identity.
(TIF)

S3 Fig Coupling of peak saccade speed between eyes, with and without head movements. Two-dimensional histograms show the joint distribution of right- and left-eye peak saccade speed (peak S; °/ms) for monocular and binocular saccades occurring in the absence of head movements (left) and with head movements (right), plotted over 0–0.2 °/ms with 100 × 100 bins. Each point contributing to the histogram corresponds to a saccade detected in one eye, paired with the maximum angular speed in the contralateral eye within a ±51 ms window around saccade onset. Histograms were pooled between animals for equal contribution. The dashed line indicates equality between eyes. Color denotes probability per bin (each panel independently normalized to sum to 1; color scale shared between these two panels only, not with Fig 2F).
(TIF)

S4 Fig Correlation of pupil dynamics between eyes. Correlated (r > 0.2, “Correlated”), weakly coupled (−0.2 ≤ r ≤ 0.2, “Weak”), and negatively correlated (r < −0.2, “Anti”) bilateral pupil dynamics. Fractions were computed from rolling Pearson correlations (10-s window) applied to z-scored pupil traces from all valid blocks per animal. Bars represent the time-weighted proportion of correlation states across the full dataset for each animal.
(TIF)

S5 Fig Comparative oculomotor spans across species. Main axis (blue) and Perpendicular (orange) oculomotor ranges (peak-to-peak eye-in-head rotations, in degrees) for humans, barn owl, great cormorant, mouse, and *P. vitticeps*. Bars show literature-derived estimates for humans [81], mice [34], barn owls [87], and great cormorants [10], alongside the spans measured in *P. vitticeps* in the present study.
(TIF)

S6 Fig Full system wiring diagram. Double lines = physical wiring and cables to peripherals. Solid arrows = data transfer via wire, cable, or file transfer.
(TIF)

S7 Fig Cranial anchor implantation procedure across species. **(A)** *P. vitticeps*. (1) Preoperative view, with the dorsal skin region selected for removal marked. (2) Dorsal skin and scales removed to expose the skull; intended screw implantation sites are marked. (3) Reinforcement screws implanted and the skull prepared for adhesive application and anchor placement. (4) Post-implantation photogrammetry session. The yellow tape placed over the metallic anchor reduces specular reflections and provides visual features for reconstruction from multiple viewing angles. (5) Postoperative view of the implanted cranial anchor. **(B)** *M. musculus*. (1) Preoperative preparation, showing application of ophthalmic ointment (Duratears) and positioning of the animal. (2) Dorsal skin removed and skull exposed in preparation for adhesive application. (3) Anchor positioning and adhesive application. (4) Postoperative recovery with the anchor in place. (5) Postoperative view from an additional angle illustrating anchor position on the skull. **(C)** *T. scripta elegans*. (1) Preoperative view with the dorsal skin region selected for removal marked. (2) Dorsal skin and scales removed to expose the skull; intended screw implantation sites are marked. (3) Reinforcement screws implanted and the skull prepared for adhesive application and anchor placement. This surgery was performed concurrently with implantation of an intracranial electrode; the visible craniotomy is unrelated to and is not required for cranial anchor implantation. (4) Postoperative view of the cranial anchor without the turtle-specific extender. (5) Cranial anchor fitted with the extender module, which positions the headmount attachment point outside the carapace and allows full head retraction without contact between the carapace and the anchor/headmount assembly.
(TIF)

S8 Fig Cross-species eye-tracking and quantitative characterization of mouse eye movements. **(A)** Representative eye-video frames and corresponding horizontal (φ) and vertical (θ) eye-position traces from lizard (*P. vitticeps*), mouse (*M. musculus*), and turtle (*T. scripta elegans*). All traces are zeroed to animal rest, computed as the median eye position across each recording, for each eye. **(B)** Mouse saccade angular-speed profiles aligned to peak speed. Other same-eye saccades occurring within the ±100-ms window were masked. **(C)** Mouse eye-position profiles corresponding to the isolated saccades shown in (B). **(D)** Relationship between saccade amplitude and peak angular speed in mice. Amplitude was calculated from onset to offset, or from the pre-onset position to offset for single-frame events, consistent with S13 Fig. **(E)** Interocular peak-speed coupling for all detected saccades in *P. vitticeps* (left; detection threshold 0.8°/frame) and *M. musculus* (right; 3.23°/frame), a zoomed-out version (top) and a zoomed-in version (bottom) are shown. The same relative subset and histogram binning are used for both species. Because head movement annotations were unavailable for mice, the lizard data are shown without the head-stationary filtering used in Fig 2F.
(TIF)

S9 Fig Camera jitter at the imaged eye plane. Displacement histograms for rigid (single-piece) lizard and modular lizard/mouse /turtle mounts. Medians: mouse = 28 μm (2.2 px), turtle = 80 μm (2.0 px), modular lizard = 90 μm (2.0 px), and rigid lizard = 42 μm (1.0 px). 95th percentiles: mouse = 90 μm (7.1 px), turtle = 179 μm (4.5 px), modular lizard = 211 μm (5.0 px), and rigid lizard = 173 μm (5.4 px).
(TIF)

S10 Fig Frame-to-frame eye-tracking noise in quiet intervals. **Left:** example traces from the manually picked quiet intervals. **Right:** Histograms show interframe angular displacement D = √(Δφ^2^ + Δθ^2^) (°/frame) pooled over stationary windows (both eyes). The solid curve is a Rayleigh probability density with scale matched to the sample median. The dashed orange line marks median (D), taken as the typical noise magnitude; the dashed purple line is the saccade-detection threshold. Detection SNR = threshold/median(D). Goodness of fit used the Kolmogorov–Smirnov statistic D_KS. **(A)** Lizard (*n* = 5,640). median(D) = 0.113°/frame, B = 0.096°/frame, threshold = 0.8°/frame, SNR = 7.08, D_KS = 0.057. **(B)** Mouse (*n* = 5,204). median(D) = 0.305°/frame, B = 0.259°/frame, threshold = 3.23°/frame, SNR = 10.58, D_KS = 0.071. **(C)** Turtle (*n* = 2,692). median(D) = 0.163°/frame, B = 0.138°/frame, threshold = 2.0°/frame, SNR = 12.28, D_KS = 0.106.
(TIF)

S11 Fig Inter-saccadic interval distributions during Active and Quiet epochs, and epoch duration distributions. **(A)** Inter-saccadic interval (ISI) distributions separated by behavioral state (left = Active, right = Quiet). Intervals spanning a transition between Active and Quiet were excluded. Colors denote individual animals. **(B)** Distributions of sustained Active and Quiet epoch durations after bridging opposite-state interruptions shorter than 3 s and retaining epochs ≥5 s. Quiet-epoch durations shown at full range and on the same expanded timescale as Active epochs (5-s bins).
(TIF)

S12 Fig Post-saccadic back-and-forth sequences during head movement. **(A)** Example sequence where both eyes show the ‘back-and-forth’ reversals. Window −0.25 to +0.55 s around primary onset. Solid: primary onset; dashed: primary offset; green dotted: extras within 150 ms. **(B)** ‘Back-and-forth’ rate among large saccades during head movement.
(TIF)

S13 Fig Main-sequence relationships for all, concurrent, and monocular saccades. **(A–C)** Mean peak angular speed as a function of saccade amplitude for all (A), concurrent (B), and monocular (C) saccades. Colored lines represent individual animals; dashed lines show the population linear fit. **(D)** Peak angular speed versus amplitude for all individual saccades pooled across animals (*n* = 33,294); dashed line shows the linear fit. Saccade amplitude was calculated from onset to offset; for single-interframe-interval events, the pre-onset position was used as the starting position to capture the displacement associated with the detected velocity peak. **(E)** Comparison of per-animal ordinary least-squares (OLS) slopes for concurrent and monocular saccades. Monocular saccades exhibited a slightly steeper main-sequence relationship (concurrent: 0.0227 ± 0.0007; monocular: 0.0250 ± 0.0004 °/ms per degree, mean ± SEM; paired *t* test: *t* = −3.32, df = 4, *p* = 0.0294; *n* = 5 animals).
(TIF)

S1 Data Source data for all quantitative graphs in this study. (XLSX)

S1 Table Comparison with previously published video oculography systems for freely behaving animals. Our system was compared with the following systems: Wallace and colleagues (2013) [25], Meyer and colleagues (2018) [26], Michaiel and colleagues (2020) [34], Sattler and Wehr (2021) [27], Huang and colleagues (2022) [30], and Singh and colleagues (2025) [96]. In summary, our system has technical specifications comparable with the state-of-the-art while demonstrating a personalized reproducible approach that is adaptable to diverse head-eye geometries, thus allowing eye-tracking across species.
(PDF)

S1 Video Synchronized video example. 55 s of synchronized video output for (left-to-right) right eye, top-down arena camera, and left eye. Videos are shown raw, with eye data traces showing eye angle and pupil diameter below. red = right eye, blue = left eye.
(MP4)

S2 Video Monocular saccades example. A collection of synchronized videos for events detected as monocular saccades. Green dot denotes saccade detection. Slowed down to 20% speed.
(MP4)

## Abbreviations

ISI: inter-saccade interval
OLS: ordinary least-squares
ROI: region of interest
SNR: signal-to-noise ratio.
