# Supplementary material for: An open-source eye-tracking system adaptable to diverse terrestrial vertebrates reveals state-dependent visual acquisition in lizards
Source: PLoS Biol. 2026 Nov 3;24(11):e3004066. doi: 10.1371/journal.pbio.3004066 (PMC13754232; doi:10.1371/journal.pbio.3004066)
Supplement: Supplementary file 1 [file pbio.3004066.s001.pdf]

An open-source eye-tracking system adaptable to diverse terrestrial vertebrates reveals state-dependent visual acquisition in lizards

Nimrod Leberstein[1,2], Tali Remenick[1,2], Milan Becker[1], Mark Shein-Idelson[1,2,*]
[1]School of Neurobiology, Biochemistry, and Biophysics, Tel Aviv University, Tel Aviv, Israel
[2]Sagol School of Neuroscience, Tel Aviv University, Tel Aviv, Israel
*sheinmark@tauex.tau.ac.il

S1 Text. Supplementary methods

This supporting protocol provides practical details of cranial anchor implantation, headmount fitting, video acquisition, and analysis. Figure labels and numbered references refer to the accompanying main article.

Cranial Anchor Implantation and Photogrammetry

This procedure describes a protocol for surgically implanting a lightweight cranial anchor for the eye-tracking headmount onto the skull of *P. vitticeps*. The same overall approach was used in turtles and mice, with species-specific anchor sizes and fixation as described below. Representative stages of the implantation procedure across all three species are shown in S7 Fig.

Before surgery, animals received analgesic, anti-inflammatory, and antibiotic treatment to minimize discomfort and infection risk. We administered meloxicam at 0.2 mg/kg or carprofen at 2 mg/kg (diluted to 1 mg/ml), enrofloxacin at 5 mg/kg (diluted to 1 mg/ml), and dexamethasone at 0.5 mg/kg. All surgical materials were prepared in advance and instruments were thoroughly sterilized. The anchor location was chosen along the midline of the dorsal skull in regions where the bone is sufficiently thick to support shallow drilling and a strong adhesive bond.

Anesthesia was induced in an isoflurane induction box and the animal was then intubated and ventilated with 4% isoflurane (AWS 100, Hallwell EMC). The animal was placed in a stereotactic apparatus (RWD 68409) on a heating pad set to 35°C to maintain body temperature. Ophthalmic ointment (Duratears) was applied to both eyes to prevent drying. The skin overlying the dorsal skull was disinfected with 10% povidone iodine, rinsed with double-distilled water, and coated with a thin layer of 2% lidocaine ointment. We exposed only the portion of the skull where no muscle lies between skin and bone: a blunt tool was used to palpate the transition between muscle and bare skull, and a marker pen was used to delineate the skin area to be removed. Representative examples of implantation-site marking and subsequent skull exposure are shown in S7A1 Fig, S7A2 Fig, S7B1 Fig, S7B2 Fig, S7C1 Fig and S7C2 Fig. This patch of skin was then excised carefully with a scalpel so as not to damage the underlying bone. Residual soft tissue was cleaned using a micro curette, and remaining fragments were dissolved with 30% hydrogen peroxide until a clean, dry bony surface was

visible. Where applicable (*T. scripta elegans* and *P. vitticeps,* but not *M. musculus*), stainless-steel screws were added to strengthen anchor fixation. In these cases, 2-4 M1 pilot holes were drilled into stable, visibly thick areas of the skull, with screw insertion depths of 0.4–0.5 mm, taking care not to breach the cranial cavity. Representative reinforcement-screw placement in *P. vitticeps* and *T. scripta elegans* is shown in S7A3 Fig and S7C3 Fig, respectively; screws were not used for the mouse implantation (S7B3 Fig). Short, sterilized M1 screws were inserted into these holes and tightened just enough to verify rigid stability without applying excessive force to the bone. The exposed skull was then etched using Dentaflux (gel de Acido grabador azul) and coated with Evetric Bond to create a uniform adhesive surface. The anchor itself (M3 brass hexagonal standoff weighing 0.6 grams for lizards and turtles, smaller M2 plastic hexagonal standoffs weighing 0.15 grams for mice) was prepared by drilling a small circular indentation into its circumference near the base to provide mechanical purchase for the adhesive. Using the stereotaxic arm, the anchor was positioned over the prepared midline site and adjusted so that it was level and correctly oriented to support the future camera geometry. UV-curing adhesive (Fusion-Flo Light Cured Nano Hybrid Flowable Composite, Prevest DenPro Ltd.) and dental cement (Coral Fix) were applied to connect the etched skull surface, the reinforcement screws (when present), and the anchor base into a single rigid construct. The dental cement was allowed to dry completely before any further handling. Examples of the completed implanted anchor are shown in S7A5 Fig, S7B5 Fig, and S7C4 Fig.

We proceeded to photogrammetry while the animal was still under anesthesia. The goal of this step was to obtain a precise 3D model of the head, including the eyes and the implanted anchor, for subsequent virtual headmount fitting. We used a Sony RX100-II camera to acquire sharp, overlapping views of the head from multiple angles. Lighting conditions did not need to be spatially uniform, but they were kept stable during acquisition. Changes in illumination between images degrade reconstruction quality. For each animal, we collected 40–80 high-quality images covering all relevant angles and features of the head (including the anchor and both eyes), ensuring that each image was in focus and free of motion blur. For a precise reconstruction, the animal must remain as still as possible. If movement occurred before sufficient images were acquired, the partial dataset was discarded and imaging restarted. Uniform and/or reflective backgrounds such as stainless-steel tables, plain white walls and reflective objects were avoided, as the reduced feature richness introduced reconstruction artifacts. To reduce specular reflections from the metallic anchor, we applied a small piece of masking tape marked with a high-contrast pattern over the exposed socket (S7A4 Fig), which substantially improved reconstruction of this region because reflective metallic surfaces otherwise change appearance across viewing angles. The exact Meshroom settings used for reconstruction and an example dataset are provided in the associated project file in the project repository.

For *T. scripta elegans*, the implanted cranial anchor also served as the connection point for a species-specific extender module (S7C4 Fig and S7C5 Fig). The extender displaced the eventual headmount attachment point approximately 50 mm from the skull, allowing the animal to retract its head fully into the carapace without collision between the carapace and the headmount assembly. The implanted anchor without and with the extender installed is shown in S7C4 Fig and S7C5 Fig, respectively.

After photogrammetry, animals were allowed to recover in a warmed enclosure until they resumed normal behavior. Ectothermic animals (P. vitticeps and T. scripta elegans) typically resumed normal activity within approximately 12 hours, whereas mice recovered after approximately 2 hours. Postoperative care included close monitoring during recovery and administration of analgesics and antibiotics at the same dosages as in the preoperative phase for five consecutive days. Animal weight

was monitored weekly as an indicator of stress. Individuals whose weight fell below 80% of their pre-surgical baseline were euthanized according to institutional guidelines.

Headmount Design and Virtual Fitting

The photogrammetry-derived 3D mesh served as the anatomical basis for individualized headmount design. We imported the mesh into Autodesk Fusion 360 and rescaled it to true physical dimensions by matching the digital representation of the implanted hexagonal standoff to its measured real-world size. The mesh was then cleaned to remove background details and lightly decimated to facilitate interactive manipulation while maintaining details in critical regions like the eyes and anchor. Predefined modular components including the camera holders, mirror holders, and anchor interface, were loaded into the workspace as independent bodies. To form the headmount, these modules were positioned separately in their proper locations relative to the animal mesh, anchor and eyes, then joined together to create a single-piece rigid model. First, the anchor interface was positioned in the orientation defined by the anchor's actual position on the 3D animal reconstruction. Next, the camera-mirror assemblies were oriented so that each camera's optical axis intersected the eye via the reflective surface of its corresponding mirror (the camera-mirror geometry is predefined by the interaction of the camera module and the mirror holder, ensuring reproducible angles even when a mirror breaks and is replaced). Camera inclination was defined relative to a virtual horizontal axis connecting the two eye centers, with inclinations of 20–30°. The camera and mirror positions were adjusted to preserve an unobstructed optical path, with working distances of approximately 1.5–5 cm*. Cameras* were typically above the eyes, facing outward and downward toward the mirrors (Fig 1B). Once alignment was finalized, the modular elements were fused into a single rigid body using either a loft-based procedure or the generative design option in Fusion 360. This single-piece headmount was then exported and prepared for printing using Formlabs's PreForm software. Because the headmount must preserve precise geometric relationships, functional surfaces such as the internal faces of the camera holders and the hexagonal recess were kept free of support structures by carefully choosing printing orientation (support touchpoints leave small bumps or indentations on the finished 3D print even after removal and cleaning). The resulting file was printed in a Formlabs Form 2 using Grey V4.1 resin at a 25-μm layer thickness, producing headmounts that were mechanically robust, dimensionally consistent, and sufficiently lightweight for use in freely behaving small animals.

To facilitate adoption, we include a Fusion 360 file with adjustable components as part of the project repository ('Virtual_Fitting_Main.f3d'), and include a step-by-step guide for its usage.

Camera and Optical System

The eye-tracking cameras were based on commercially available Arducam NoIR modules (OV5647 sensor), which were mechanically and optically modified to allow infrared imaging at short working distances. Each camera was first stripped of its stock plastic lens assembly to expose the bare sensor and accommodate a custom M7 threaded lens mount. For lizards and turtles, cameras were fitted with 3.7-mm f/2.55 manual-focus M7 lenses (QILENS); mice were recorded using 4.5-mm f/2.8 M7 lenses. Custom-cut infrared-pass filters (>930 nm) were attached to the lenses. Filters were cut into circular discs approximately 1 mm in diameter using a laser cutter. To affix the filter without introducing optical artifacts, the lens and filter were held together under uniform pressure using a small custom-built vice with soft-foam contact faces, and a single micro-drop of cyanoacrylate was applied at the perimeter. The modified lens was inserted into the glued-on M7 threaded mount on the camera board. Care was taken to avoid both glue vapor contamination and mechanical stress on the sensor, as any residue on the sensor surface would markedly degrade image quality. Each camera holder module of the headmount provided two angled slots adjacent to the camera holders for mounting illumination

LEDs. Into these, two 940 nm infrared LEDs (Vishay VSMB2943GX01) were positioned and permanently affixed using UV-curing adhesive or cyanoacrylate. LED orientation and slot geometry were predefined to produce uniform illumination of the eye through the IR mirror without producing glare. The four LEDs of a headmount were connected to a Cyclops LED driver for controlled illumination and programmed LED-off events used for synchronization. The cameras were aimed at the eyes using small 8x8 mm infrared-reflective mirrors (940 nm), affixed to holders with dedicated grooves to guide mirror placement, which fit within a slot on each camera module to support the mirror in front of the camera. Since this is the most at-risk part of the headmount as the mirror protrudes from the head boundary, we made the mirror holder easily replaceable. The mirror-camera geometry is hard-coded in the modular design, preserving the angle between camera, mirror, and eye when replaced. During each session, camera focus was adjusted manually by threading the lens along the M7 mount while viewing a live feed on an HDMI-connected display attached to each Raspberry Pi. Final focus was set to produce a crisp pupil contour. To prevent mechanical load on the animal, camera and LED cables were routed through a counterbalanced pulley system. A thin fishing line passed through two freely rotating plastic rollers above the arena, with an adjustable water-filled counterweight on one end and a metal clip holding the camera cables on the other. The counterweight was tuned for each session such that the headmount was neutrally balanced at the center of the arena. Cables were relatively long (2 meters from pulley) to minimize shear forces on the animal head during behavior.

Raspberry Pi Video Acquisition and Synchronization

Eye videos were acquired at 640 × 480 pixels and 60 frames/s using Raspberry Pi 4B computers running custom Python software based on libcamera and picamera2. Each Raspberry Pi controlled one camera, encoded videos in H.264, and generated a digital frame-acquisition signal recorded by Open Ephys. Eye-camera frame events, LED-state signals, and external video events were recorded concurrently to construct an initial correspondence between frames and Open Ephys timestamps. The Cyclops driver suspended LED illumination for 32 ms at the end of each minute. These interruptions were visible in the eye videos and provided an independent synchronization check against the recorded LED-state events. LED-off events were automatically detected and compared across modalities. When discrepancies occurred, a small temporal correction was applied through a custom Python interface. After verification, a per-block synchronization table linked eye-video frames, external video frames, and accelerometer data. All analyses used these verified time bases (S6 Fig and S1 Video). Recording sessions typically lasted 3–4 hours and were divided into 30-minute blocks.

Pupil positions were obtained using DeepLabCut (DLC) with eight points along the pupil boundary, and two marking the rostral and caudal tear ducts. Networks were trained using the standard ResNet-50 backbone. Frames with low-confidence DeepLabCut detections (likelihood <0.9), including pupil occlusion during blinking or nictitating-membrane movement, were excluded from subsequent eye-movement analyses. Additional frame rejection was performed downstream: frames with abnormally small pupil diameters (indicative of partial eyelid closure) were removed automatically, and segments with erratic or unstable pupil-diameter behavior were flagged and manually reviewed using a custom inspection tool available in the project repository.

For each valid frame, the pupil contour was fit with a direct least-squares ellipse [59], yielding the pupil center, axis lengths, and ellipse rotation angle. These parameters served as the basis for pupil-diameter estimates and for reconstruction of eye orientation. Angular gaze coordinates (φ, θ) were computed using a geometric method which infers azimuth and elevation from pupil-center displacement relative to a reference position and the ellipse minor/major axis ratio [25]. A zero-angle

reference was defined separately for each eye and recording block. Frames in which both LED reflections were simultaneously visible in the pupil were identified using a dedicated GUI. The known LED–camera geometry was used to identify the pupil location corresponding to the camera axis for each eye. This defined the camera-centric zero-angle reference for reconstructing the eye vector.

Reconstruction accuracy was evaluated using a synthetic dataset generated in Blender, in which a 3D eye model was rotated through known azimuth–elevation combinations from −60° to +60°. The same ellipse-fitting and reconstruction pipeline was applied to the rendered images. Angular errors increased with eccentricity, from approximately 1° near the optical axis to approximately 5° at the maximal eccentricities reached by *P. vitticeps* when camera tilt was taken into account (S1A Fig). Before analysis, φ and θ traces were centered on each eye's resting position by subtracting their block-wise medians. To assess errors in these relative angular measurements, raw and corrected eye-position estimates were independently centered on their respective resting positions, and their residual difference was quantified (S1B Fig).

Saccade Detection and Segmentation

Saccades were detected independently for each eye from frame-to-frame changes in reconstructed azimuth and elevation (φ, θ). Detection used unsmoothed interframe angular speed. The lizard detection threshold was 0.8°/frame, equivalent to 48°/s at 60 frames/s; thresholds for mouse and turtle data were 3.23°/frame and 2.0°/frame, respectively (S8 Fig and S10 Fig). Consecutive suprathreshold frames were grouped into candidate events. Events were subdivided whenever the instantaneous movement direction changed by more than 90°, and only segments meeting the minimum duration of two frames and net angular displacement >0.5° were retained. Onset was the first suprathreshold frame; offset was the first frame after peak speed at which speed fell below threshold. For amplitude measurements, displacement was calculated from onset to offset; for single-interframe-interval events, the pre-onset position was used to capture the displacement associated with the velocity peak (S13 Fig). Left- and right-eye saccades with onsets differing by <34 ms were classified as concurrent (synchronized binocular) events. All remaining events were candidate monocular saccades. To verify monocular events, the maximum contralateral angular speed was measured within ±51 ms of ipsilateral onset, regardless of whether a contralateral saccade had been detected. Candidate events were retained as verified monocular saccades only when contralateral speed remained below the detection threshold; events with unavailable contralateral traces were excluded from this verified set. Reported monocular fractions used the verified subset (S2 Video). When accelerometer data were available, a saccade was labeled as occurring with head movement if its duration overlapped an accelerometer-defined movement event. For display, the speed profiles in Fig 2C were aligned to peak speed and smoothed with a 10-ms Gaussian kernel; the corresponding displacement traces in Fig 2D used the same alignment.

Pupil Diameter Analysis

Pupil diameter was computed from the major axis of the fitted ellipse in each valid frame. Pixel-to-millimeter conversion used the per-eye calibration derived from anatomical landmarks, as in the camera-jitter analysis. For comparison across animals, pupil diameters were normalized within each animal. To examine behavioral modulation of pupil size, diameter traces were aligned to behavioral-state annotations and grouped into Quiet and Active epochs. Distributions were constructed from all valid samples within each state, combining left and right eyes per animal and clipping extreme outliers. For each animal, values were z-scored relative to the pooled distribution across both states, and Active–Quiet difference curves were computed in matched diameter bins to visualize shifts in sampling probability. For each animal, state-dependent differences in median pupil diameter were

assessed by shuffling the Active/Quiet labels within each block and eye while retaining the pupil time series, generating a null distribution for a two-sided permutation test. Inter-ocular coordination was quantified using rolling Pearson correlations (10-second windows, requiring at least one-third of samples). Correlation values were categorized as positively correlated ($r > 0.2$), weakly coupled ($-0.2 \le r \le 0.2$), or anti-correlated ($r < -0.2$). Time spent in each category was computed separately for Active, Quiet, and all epochs. These metrics were used directly for the state-dependent coupling analyses (Fig 3 and S4 Fig).

Behavioral State Segmentation

Behavioral state was quantified using the three-axis accelerometer embedded in the Intan RHD-2132 headstage, recorded directly by the Open Ephys system and synchronized with the eye videos as described above. Raw acceleration traces were first filtered and then processed to estimate baseline noise levels. Low-variance segments were identified using kurtosis-based criteria and the standard deviation of these segments served as an estimate of sensor noise. Accelerometer samples were classified as movement when the magnitude of the filtered signal exceeded this noise baseline by several standard deviations. Movement events were then converted into a continuous state annotation using a rolling analysis. For each 10-s window (advanced in 1-s steps), the mean movement magnitude was computed. Windows whose mean exceeded a manually selected threshold were labeled Active, and all others labeled Quiet. External behavioral videos taken from above the animal were inspected to validate accelerometer-based labels. Consecutive windows with matching labels were merged into behavioral epochs. The unsmoothed annotations, including epochs ≥1 s, were used for the state-dependent analyses in Fig 3. For the sustained-state duration analysis in S11B Fig, interruptions shorter than 3 s were bridged, and resulting epochs shorter than 5 s were excluded.

Directional Eye Movement Analyses

Directional structure of eye movements was quantified from the net angular displacement of each segmented subsaccade. For every saccade event, the start and end gaze positions in azimuth and elevation (φ, θ) were extracted, and the displacement vector ($\Delta\varphi$, $\Delta\theta$) was computed. To visualize directionality independent of absolute gaze position, all saccades were translated so their starting point lay at the origin, yielding an eye-centered endpoint map for each eye and animal. For the endpoint heatmaps in Fig 2H, densities were estimated using a two-dimensional Gaussian kernel on a ±20° grid in the unrotated camera axes. A dominant movement axis was identified for each eye by scanning orientations from 0° to 180° and selecting the axis with the greatest number of saccade events. Directional distributions were rotated to align this axis to 0°/180° for Fig 2I. Directional bias was quantified relative to this aligned axis. For each animal, we computed:

$$bias = \frac{P(along-axis) - P(perpendicular)}{P(along-axis) + P(perpendicular)}$$

where “along-axis’’ saccades were defined as those whose displacement direction lay within ±45° of the dominant axis, and “perpendicular’’ saccades were defined relative to the orthogonal axis. Bias values were computed separately for synchronized and monocular saccades, using the same rotation for both categories.

Because world-referenced head pose was not reconstructed, these analyses describe relative eye-in-head movements rather than gaze direction in world coordinates. Block-wise medians were subtracted from φ and θ before analysis to correct for small differences in resting eye position across sessions.

Video Stability Assessment and Jitter Rejection

Camera stability was quantified for each eye and recording block using a manually selected region of interest (ROI) on a stationary image feature, such as part of the headmount, a stable mirror edge, or non-moving facial scales. The ROI in each frame was compared with that in the first frame using normalized two-dimensional cross-correlation. The displacement of the correlation peak relative to the reference frame was converted to physical units using an individually calibrated pixel-to-millimeter scale derived from anatomical landmarks. These reference-relative displacements were aggregated to characterize stability across headmount configurations (S9 Fig). Separately, samples with >5 pixels of interframe image displacement were excluded from subsequent eye-movement analyses to limit artifacts caused by movement of the camera assembly relative to the eye. This exclusion criterion removed 0.21% of samples across the dataset, as reported in the main Methods. When a mirror holder was damaged, it was replaced to restore the predefined camera–mirror geometry.

Behavioral Recording Arena

Eye-tracking experiments were conducted in the PreyTouch behavioral arena described in detail in Eyal et al. [57]. In brief, the arena measured 70 × 100 × 45 cm and was uniformly illuminated using overhead white LEDs powered by a constant 12 V, 5 A supply to avoid flicker. Four synchronized high-speed arena cameras (FLIR monochrome, 60 fps) were positioned above and at the sides of the enclosure to provide continuous coverage of the animal's full body posture and locomotion. External video events were recorded by Open Ephys to align arena recordings with eye-camera recordings and accelerometer data. For experiments requiring head-movement annotation, animals carried an Intan RHD-2132 headstage, and the three-axis accelerometer embedded in the unit was recorded synchronously through the same Open Ephys acquisition pipeline. A complete description of the arena construction, camera synchronization hardware, illumination uniformity, and optional peripheral modules (reward delivery, motion sensors, thermocontrol) can be found in Eyal et al. [57], and all relevant details for replicating the core setup are provided there.
