# Supplementary material for: An open-source eye-tracking system adaptable to diverse terrestrial vertebrates reveals state-dependent visual acquisition in lizards
Source: PLoS Biol. 2026 Nov 3;24(11):e3004066. doi: 10.1371/journal.pbio.3004066 (PMC13754232; doi:10.1371/journal.pbio.3004066)
Supplement: Supplementary file 2 [file pbio.3004066.s016.pdf]

Publication / system	This study	Wallace et al. 2013	Meyer et al. 2018	Michaiel et al. 2020	Sattler & Wehr 2021	Huang et al. 2022	Singh et al. 2025
Species / cross-species implementation	Lizard; turtle; mouse (3 species demonstrated)	Rat (1 species)	Mouse (1 species)	Mouse (1 species)	Mouse (1 species)	Mouse (1 species; broader small-animal adaptability proposed)	Marmoset (1 species; ~300–400 g)
Reported carried mass	Mouse: 3.5 g; lizard/turtle: ~5 g	~0.8 g per eye camera + ~0.5 g shared housing	~1.3 g single-camera system	2.6 g bilateral cameras + optics/holders + IMU	0.68 g 2-camera; 1.64 g 4-camera assembly	0.10 g single Sch1 ETU; up to 1.01 g one-eye Sch2	~20 g headpiece + ~40 g backpack
Eye-video acquisition	640 × 480; 60 Hz	752 × 480; 40–50 Hz	640 × 480; 60 Hz	480 × 640; 30-Hz interlaced → 60 fields/s	800 × 480 optical; 30-Hz interlaced → 60 fields/s	1280 × 720; 30 Hz	HD eye camera (pixel dimensions not reported); 90 fps
Bilateral eye recording	Yes	Yes	Optional / demonstrated	Yes	Yes (2-camera configuration)	Yes (two ETUs possible)	No (one eye camera)
Head-motion measurement	Motion sensor (3-axis accelerometer)	World-referenced 3D head pose (6 head LEDs + overhead cameras)	Motion sensor (accelerometer; gyroscope used in extended analyses)	Motion sensor (accelerometer + gyroscope IMU)	Motion sensor (headstage accelerometers)	Not intrinsic to ETU	Motion sensor (IMU)
Eye-position extraction	DLC pupil-edge landmarks → ellipse fit	Gradient-based pupil-boundary detection / ellipse representation	Pupil identified from registered eye images; pupil position/shape extracted computationally	DeepLabCut: 8 pupil-edge points → least-squares ellipse	System paper demonstrates eye/iris feature tracking; extraction method is application-dependent rather than a single prescribed pupil pipeline	Image segmentation + DeepLabCut tracking fused with adaptive Kalman filtering	UNet segmentation of pupil/iris from eye-camera images
Eye-angle reconstruction & validation*	Wallace-derived geometric reconstruction from pupil ellipse/camera geometry; independently validated on this dataset: relative eye-in-head reprojection residuals φ 0.84 ± 0.48°, θ 1.19 ± 0.48°	Geometric 3D eye/camera model based on ellipse deformation; model-eye rotational detection error 0.9 ± 0.1°	No calibrated angular reconstruction or ground-truth angular validation reported	Explicitly follows Wallace et al. 2013: affine/geometric conversion of pupil ellipse to angular displacement. No ground truth; reported 0.51 ± 0.25° is an upper-bound stability/error estimate during stationary epochs	Cyclotorsion quantified in degrees; no calibrated horizontal/vertical pupil-to-eye-angle reconstruction or validation reported	Tracks pupil/eye position, but no directly comparable Wallace-style angular reconstruction validation or angular ground-truth accuracy metric identified	Pupil position mapped to screen/world coordinates by per animal calibration (horizontal/vertical scale + offset). Head-fixed RMS stability 0.05°; freely moving median gaze error <1°
Mechanical stability characterized	Yes: image-plane displacement distributions (overall displacement compared with recording beginning) (Fig S9)	Yes: camera displacement estimated from anatomical eye landmarks and corrected; no directly comparable physical jitter statistic	Yes: mean inter-frame registered image motion <4 µm freely moving; <1 µm head-fixed	Yes: RMS frame-to-frame camera shake 0.49 ± 0.33 px	No quantitative jitter metric identified	No directly comparable jitter metric identified	No directly comparable mechanical-jitter metric identified
Recording longevity / repeated-use window	Up to ~6 months (lizard); ~2 months (turtle); ~4 weeks (mouse)	Not reported	Not reported	Not reported	Not reported	Not reported	Repeated sessions demonstrated; maximum viable system lifetime not reported as a comparable metric

**Eye-in-image / usable eye coverage**	Quantified across species (eye ROI occupancy reported relative to frame dimensions)	Not reported as an equivalent eye in-frame occupancy metric	Not reported as an equivalent eye-in-frame occupancy metric	Not reported as an equivalent eye-in-frame occupancy metric	Not reported as an equivalent eye-in-frame occupancy metric	Not reported as an equivalent eye-in-frame occupancy metric	Not reported as an equivalent eye-in-frame occupancy metric
**Individual-anatomy-derived headmount geometry**	Yes: rigid workflow uses individual photogrammetry	No: camera positioned using adjustable ball joint	No: generic printed holder; mirror adjusted in situ	No: connectors positioned/fixed during fitting	No: standardized printed carriers; position/focus adjusted as needed	No: connector position adapted during fitting	No: custom-fitted/adjusted per animal, but not derived from 3D anatomical reconstruction
**Predefined rigid camera–eye geometry**	Yes (rigid); No (modular)	No: camera position adjusted to eye	Partial: holder fixed; mirror adjusted then permanently fixed	Partial: alignment established during initial fitting then fixed	Yes for fixed 4-camera carrier; adjustable 2-camera variant also available	No: detachable ETU position adjusted through connector placement	Partial: repeatable after per-animal fitting to two headposts
**Multiple system configurations**	Individualized photogrammetric rigid headmount + modular in-situ fitted headmount; bilateral configurations	Bilateral eye-camera system with adjustable camera positioning	One- or two-camera implementations; adjustable mirror/camera geometry	Primarily bilateral: two eye cameras + IMU; alignment fixed after fitting	Fixed 4-camera multimodal carrier + adjustable 2-camera headset	One or two detachable ETUs; direct-view (Sch1) and NIR-reflector (Sch2) configurations	Single eye + world-camera headpiece; components custom-fitted to animal
**Open hardware / design resources**	Yes	Detailed supplementary construction/calibration methods; no dedicated open source repository + highly [illegible]	Yes: STL + acquisition resources	Partial: analysis/data resources; no equivalent complete open hardware package	Yes: detailed build + printable files	Yes: code/tracking resources; hardware construction described	Yes: headpiece STL + pupil-tracking GUI/code
