# Supplementary material for: An open-source eye-tracking system adaptable to diverse terrestrial vertebrates reveals state-dependent visual acquisition in lizards
Source: PLoS Biol. 2026 Nov 3;24(11):e3004066. doi: 10.1371/journal.pbio.3004066 (PMC13754232; doi:10.1371/journal.pbio.3004066)
Supplement: Supplementary file 4 [file pbio.3004066.s022.pdf]

Response to reviewers

We thank the reviewers for their helpful comments and suggestions. We have attempted to answer each and every one of their comments and questions. For clarity, we colored the reviewers' comments in **blue**, our responses in **black**, and the changes we made in the manuscript are marked in **red**. We have numbered the reviewer's comments and reference them accordingly in this response.

Reviewer #1

Leberstein et al present a detailed and innovative study outlining a lightweight, customizable eye tracking system amenable across the terrestrial animal species. Their system is clearly useful in mouse, turtles, and lizards--with a deeper exploration into oculomotor and binocular behaviors of freely moving lizards. The paper is well written and clear, difficult given the difficulties in implementing these types of hardware and software/analyses needed to estimate egocentric gaze. In addition, I think it's an excellent resource just in terms of the exhaustive list of historical and recent papers referenced in the field of freely-moving animal eye tracking (I will be asking students in my lab to use this paper as a reference). I consider my comments minor, but I would like to see them address in a revised version of this manuscript.

We thank the reviewer for finding our study "detailed and innovative", "clearly useful", and "well written". We hoped that it would contribute to the community of eye tracking research and we are therefore very happy that the reviewer thinks it's an "excellent resource" for students.

Comment #1: There is a lack of details on the surgical implantation and I would like to learn/see more. Perhaps some images as a supplemental are all that are required, but this seems important given that different methods of implanting the hexagonal screw anchor across animals.

To clarify the implantation procedures, we added a supplementary Fig S7 which illustrates the anchor implantation process and the different implantation configurations used across species. This information is now referenced in the Results section:
"Importantly, this scan followed a surgical implantation of a single hexagonal screw anchor point for connecting the headmount (Fig 1 and S7; Methods)".

And the Methods section:
"A detailed surgical protocol is provided in Supplementary Methods (Document S1). The implantation procedure and anchor configurations used across species are illustrated in Fig S7."

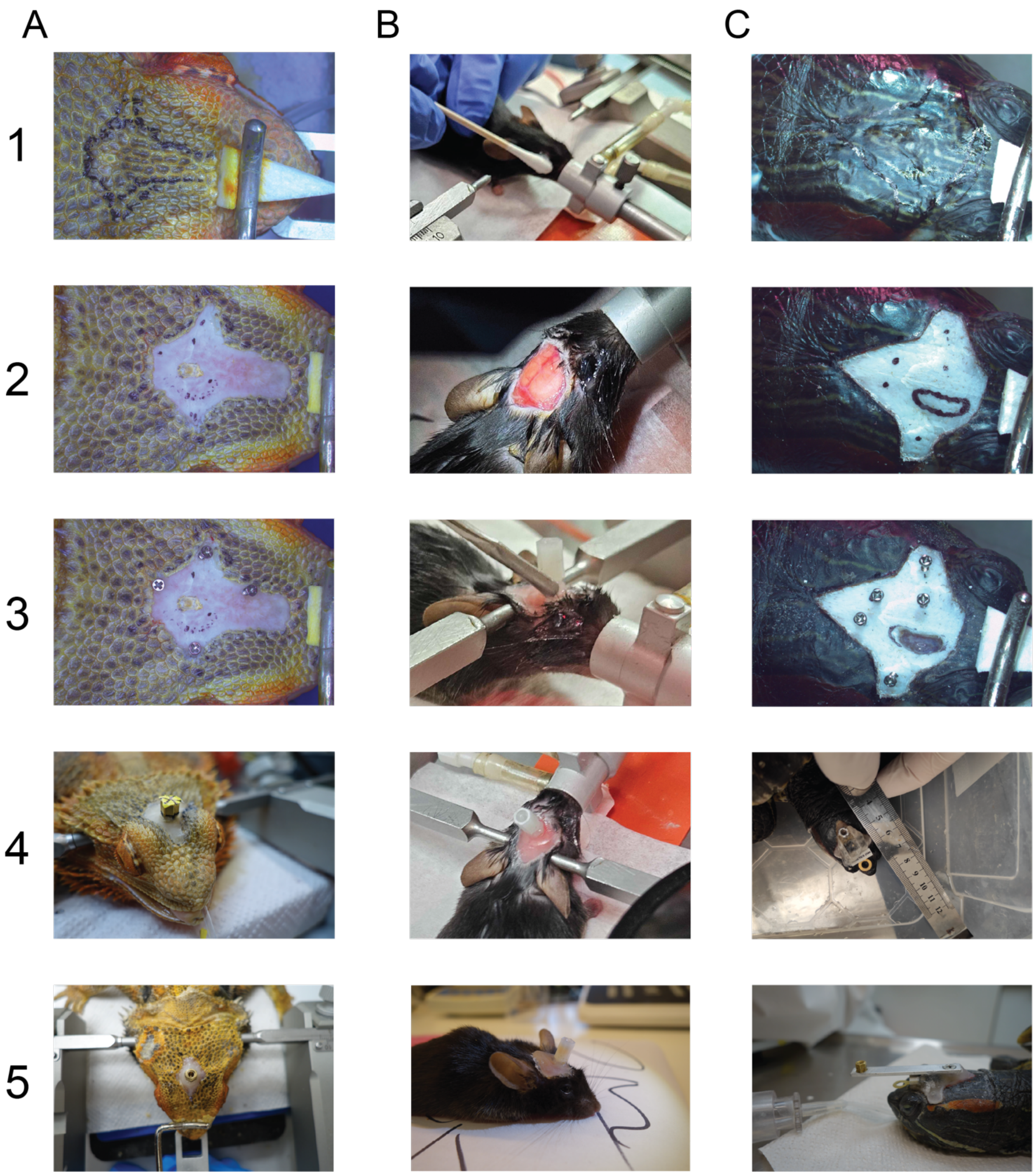

"**Fig S7. Cranial anchor implantation procedure across species. (A)** *P. vitticeps*. (1) Preoperative view, with the dorsal skin region selected for removal marked. (2) Dorsal skin and scales removed to expose the skull; intended screw implantation sites are marked. (3) Reinforcement screws implanted and the skull prepared for adhesive application and anchor placement. (4) Post-implantation photogrammetry session. The yellow tape placed over the metallic anchor reduces specular reflections and provides visual features for reconstruction from multiple viewing angles. (5) Postoperative view of the implanted cranial anchor. **(B)** *M. musculus*.

(1) Preoperative preparation, showing application of ophthalmic ointment (Duratears) and positioning of the animal. (2) Dorsal skin removed and skull exposed in preparation for adhesive application. (3) Anchor positioning and adhesive application. (4) Postoperative recovery with the anchor in place. (5) Postoperative view from an additional angle illustrating anchor position on the skull. **(C)** *T. scripta elegans*. (1) Preoperative view with the dorsal skin region selected for removal marked. (2) Dorsal skin and scales removed to expose the skull; intended screw implantation sites are marked. (3) Reinforcement screws implanted and the skull prepared for adhesive application and anchor placement. This surgery was performed concurrently with implantation of an intracranial electrode; the visible craniotomy is unrelated to and is not required for cranial anchor implantation. (4) Postoperative view of the cranial anchor without the turtle-specific extender. (5) Cranial anchor fitted with the extender module, which positions the headmount attachment point outside the carapace and allows full head retraction without contact between the carapace and the anchor/headmount assembly."

In addition, we refer to Fig S7 in various locations throughout the surgical protocol in Document S1.

Comment #2: The photogrammetry is clever, but I wonder how well this solution works for animals with large amounts of skin or muscle on top of their head. How important is it to estimate the skull-eye relationship for this technique to be useful?

To clarify the generality of the photogrammetry we added the following text to the Methods and Results sections.
"The relevant features for virtually fitting the camera position to each animal, are the geometrical relationship between the scanned implanted anchor (that is connected to the skull) and the eyes, together with the head geometry required to verify an unobstructed optical path between the eye, mirror, and camera. Estimation of the skull-eye relationship is therefore not required."

"We did not encounter difficulties due to differences in overlying skin or muscle in the species we tested. The broad application of photogrammetry to diverse subjects suggests that this is not an inherent limitation of the technique itself. [114] However, it is critical that both the implanted hexagonal screw and the eyes are visible in the photogrammetry scan."

Comment #3: Camera specs are important and these details should be directly added to the main text. In addition, I was wondering if a framerate of 60 Hz and the pixel resolution used, given the eye size on the sensor, had any limitations in detecting certain speed of saccades?

To address this comment, we added the camera specs to the main text, improved the clarity of the Methods section and calculated the spatial and temporal limits of the system.
The Results now read:
"To achieve sharp eye images at short working distances without background contamination, the cameras were fitted with close-focus lenses and IR-pass filters, with two IR LEDs providing stable illumination of each eye (Fig 1D; Methods). This setup allowed video acquisition at 640x480 pixels

and 60 frames/s with stable illumination of the eye and synchronization verification via programmed LED power-off windows (Methods)."

The video acquisition in Methods now reads:
"**Video acquisition camera specifications and optics**

Eye videos were acquired using miniature IR-sensitive Arducam B0066 "NoIR" board cameras equipped with OV5647 1/4-inch, 5-MP sensors and mounted on the custom headmount. To enable sharp imaging at short working distances, the stock optics were replaced with manual-focus lenses. For lizards and turtles, cameras were fitted with 3.7-mm f/2.55 M7 pinhole lenses; for mice, 4.5-mm lenses with a narrower field of view were used. Custom IR-pass filters (>850 nm) were fitted to the lenses to minimize visible-light contamination. Cameras viewed the eyes indirectly via 940-nm reflective mirrors that remained visually transparent to the animals, allowing close-range imaging while keeping the cameras outside the direct field of view. Two 940-nm IR LEDs mounted adjacent to each camera provided illumination.

Camera configuration and video acquisition were controlled using custom Python code running on each Raspberry Pi via SSH. Videos were acquired at 640×480 pixels and 60 frames/s, with working distances of approximately 1.5–5cm. A digital frame-acquisition event was generated for every captured frame and transmitted to the Open Ephys acquisition system for temporal alignment across data streams. The IR LEDs were powered by a Cyclops LED driver, which was programmed to suspend illumination for 32 ms at the end of each minute while transmitting an LED-ON event to the Open Ephys acquisition board. These programmed illumination interruptions provided an additional offline synchronization marker between the eye videos, accelerometer data, and external behavioral recordings. Recording sessions, typically lasting 3–4 h, were divided into 30-min blocks."

In addition, we calculated the spatial and temporal limits of the system (S10) and discussed these results in the Methods:

"Spatiotemporal limitations

The temporal resolution of eye-movement measurements was limited by the 60-Hz camera frame rate (~16.7 ms between frames). Consequently, velocities represent interframe angular displacements and do not resolve sub-frame velocity profiles; movements that occur and return near their initial position within a single interframe interval cannot be detected nor can changes in velocity occurring at time scales faster than the inter frame interval. Spatial resolution is limited by pixel size, magnification, and spatial noise from pupil ellipse fitting.

The combined tracking noise was estimated empirically from frame-to-frame angular gaze displacement during stationary-eye periods (Fig S10). For each consecutive frame pair, displacement was computed as $D = \sqrt{\Delta\varphi^2 + \Delta\theta^2}$ [°/frame]. Under the assumption that the per-axis angular increments ($\Delta\varphi$, $\Delta\theta$) are independent and Gaussian with equal standard deviation, D follows a Rayleigh distribution. To corroborate this, we compared each quiet-window sample to a analytical Rayleigh distribution with scale matched to the sample median, using the Kolmogorov–Smirnov statistic DKS (largest gap between empirical and model CDFs; Fig S10). Maximum gaps were small (lizard DKS = 0.057, mouse DKS = 0.071, turtle DKS = 0.106). We

took the sample median of D as the typical noise magnitude. Detection signal-to-noise ratio (SNR) was defined as the saccade-detection threshold divided by this median. For the lizard data in Fig S10 (5,640 frame-to-frame steps from five stationary segments, both eyes), the noise value for the interframe eye displacement was: median(D) = 0.11°/frame (~6°/sec). **While it is possible to extract eye movements at lower speeds, we set a conservative detection threshold of 0.8°/frame (~50°/s) that is considerably higher than the noise (SNR = 0.8 / 0.113 = 7.08). For mice, median(D) = 0.31°/frame and the detection threshold was 3.23°/frame, corresponding to SNR = 3.23 / 0.31 = 10.59."**

We additionally refer to the above calculation in the Results:

"To characterize the kinematics of saccadic eye movements in *P. vitticeps*, we identified saccades using an angular speed threshold of 0.8°/frame (~50°/s) applied to gaze-angle traces. This threshold was well above the empirically measured eye position tracking noise during verified no-eye-movement epochs (SNR=7.08; Fig S10; Methods)."

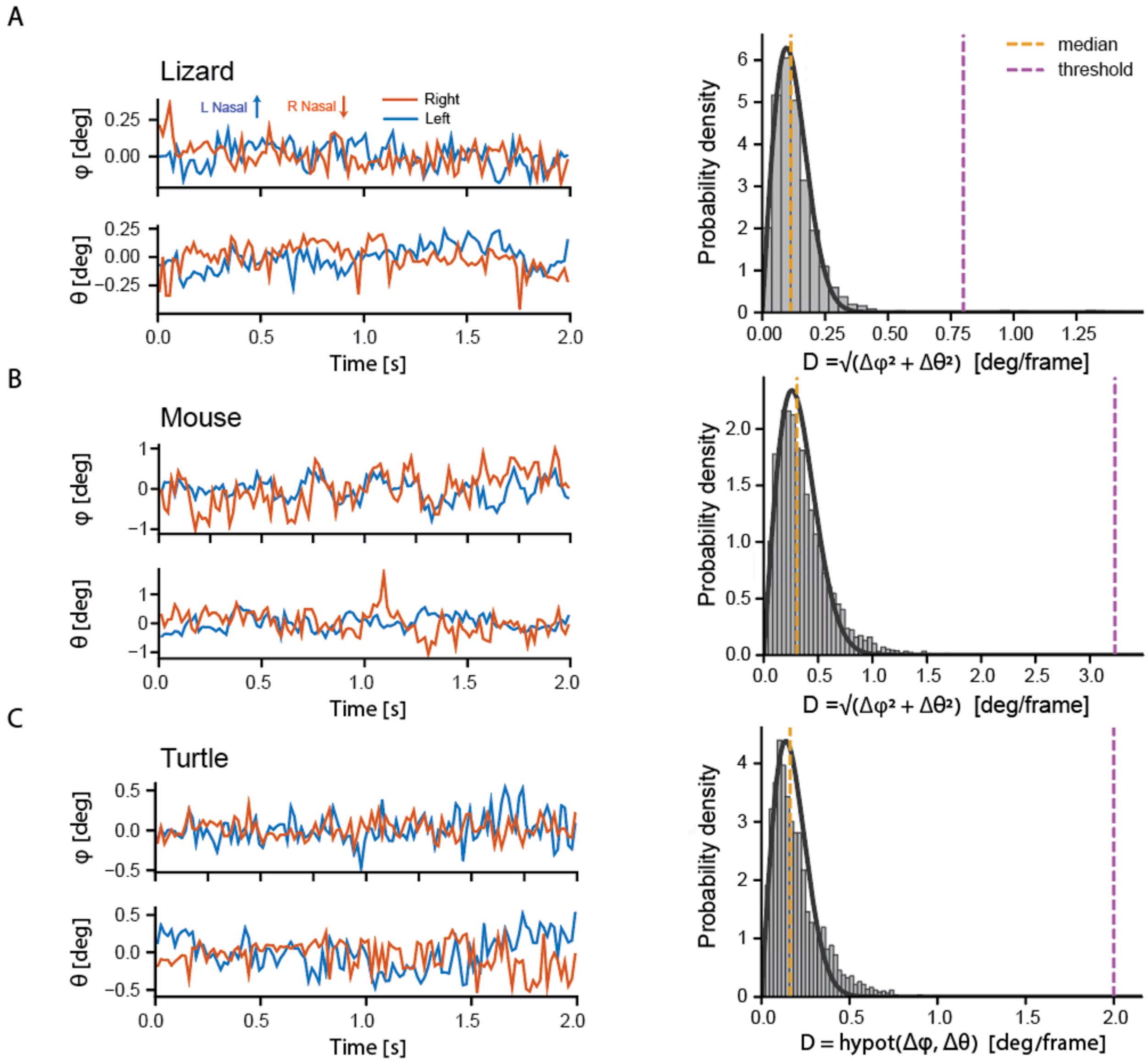

"Fig S10. Frame-to-frame eye tracking noise in quiet intervals.

Left: example traces from the manually picked quiet intervals. Right: Histograms show interframe angular displacement $D = \sqrt{(\Delta\varphi^2 + \Delta\theta^2)}$ (°/frame) pooled over stationary windows (both eyes). The solid curve is a Rayleigh probability density with scale matched to the sample median. The dashed orange line marks median(D), taken as the typical noise magnitude; the dashed purple line is the saccade- detection threshold. Detection SNR = threshold / median(D). Goodness of fit used the Kolmogorov–Smirnov statistic D_KS.

(A) Lizard (n = 5640). median(D) = 0.113°/frame, B = 0.096°/frame, threshold = 0.8°/frame, SNR = 7.05, D_KS = 0.057.

(B) Mouse (n = 5204). median(D) = 0.305°/frame, B = 0.259°/frame, threshold = 3.23°/frame, SNR = 10.58, D_KS = 0.071.

(C) Turtle (n = 2692). median(D) = 0.163°/frame, B = 0.138°/frame. threshold = 2.0°/frame, SNR = 12.28, D_KS = 0.106."

Comment #4: - Please specify the source of many parts. For example, the source of the IR mirrors as there are many companies producing these size mirrors and not all are the same quality.

We have revised the system-components section of Table 1 to provide manufacturer and product information for the optical, mechanical, and surgical components used in the system, including the IR mirrors and filters.

REAGENT or RESOURCE	SOURCE	IDENTIFIER
**Software and algorithms**		
Python 3.10	Python Software Foundation	https://www.python.org
MATLAB 2025a	MathWorks	https://www.mathworks.com
Blender 4.4	Blender Foundation	https://www.blender.org
Custom code for eye camera acquisition and analysis	This paper	https://github.com/EvolutionaryNeuralCodingLab/PETS
Autodesk Fusion (computer aided design)	Autodesk	https://www.autodesk.com/products/fusion-360/overview
DeepLabCut (pose estimation)	Mathis et al.	https://github.com/DeepLabCut/DeepLabCut
Meshroom (Photogrammetry)	Alice Vision	https://alicevision.org/#meshroom
**System components**		
3D printer (Form 2)	Formlabs	https://formlabs.com/3d-printers/form-2/
3D printer resin GreyV4.1	Formlabs	https://formlabs.com/global/products/grey-resin-v4-1/
Arena cameras (FLIR Firefly FFY-U3-16S2M-DL)	Teledyne FLIR	https://www.flir.com/products/firefly-dl
Electrophysiology recording system (Open Ephys Acquisition Board)	Open Ephys	https://open-ephys.org
Head mounted accelerometer (embedded in Intan RHD2132 headstage)	Intan	https://intantech.com/RHD_headstages.html?tabSelect=RHD32ch
Raspberry Pi 4 model B	Raspberry Pi	https://www.raspberrypi.com/products/raspberry-pi-4-model-b/
Eye cameras (5MP OV5647 Miniature Camera Module with Long Flex Cable for Raspberry Pi (NoIR))	Arducam	https://www.arducam.com/1-4-5-noir-mpspy-camera-module-sensor-flex-cable-raspberry-pi.html
Manual focus lenses for eye cameras used for lizards and turtles (3.7mm, M7 thread, f/2.55)	QILens, AliExpress	link
Manual focus lenses for eye cameras used for mice (4.5mm, M7 thread, f/2.8)	QILens, AliExpress	link
IR mirror	Quanmin, Amazon	link

(8mm×8mm×0.7mm 650nm IR-Cut Blocking Filter)		
930 nm, 100 × 100 × 1 mm PMMA IR-pass filter (cut to 1mm diameter disks and fitted on camera lenses)	Nantong Foric Optical Glass Factory Store, AliExpress	link
M7 Lens holder height 6.0mm	QILens, AliExpress	link
Programmable LED driver (Cyclops)	Open Ephys	https://open-ephys.org/cyclops
High Speed Infrared Emitting Diodes, 940 nm vsmb2943rgx01	Vishay Semiconductors	https://www.vishay.com/en/product/83486/
Brass hexagonal standoff, M3, 6+6 mm F/M (lizard and turtle cranial anchor)	Uxcell store, Amazon	link
Nylon hexagonal standoff, M2, 12 mm F/F (mouse cranial anchor)	XT Hardware Store, AliExpress	link
UV adhesive (Fusion-Flo Light Cured Nano Hybrid Flowable Composite)	Prevest DenPro Ltd.	https://www.prevestdenpro.com/product/fusion-flo/
Dental Flux (Gel de Acido grabador azul)	DentaFlux	https://www.dentaflux.com/producto/acido-fosforico-37-miniacid-4x12ml-azul/
UV adhesive (Evetric Bond)	Ivoclar	https://www.ivoclar.com/hr_hr/products/adhesives/evetric-bond
UV adhesive (Transbond XT)	3M Unitek	https://www.3mindia.in/3M/en_IN/p/d/b00007525/
Coral-Fix Dental Cement	Ron Dental Depo	https://rons.co.il/product/coral-fix/
Photogrammetry acquisition camera (Sony RX100-ii)	Sony	link

Comment #5: is there anything to be gained by analyzing the corneal reflections of the two IR LEDs? This is a technique that can be used to increase accuracy of eye position and potentially estimate gaze-in-world. The fixed position of these LEDs and the stability of the head mount seem great for this kind of tracking of corneal reflections.

Indeed. The corneal reflections produced by the two IR LEDs are important and already used in our analysis. We have clarified this in the Methods text:

"The two IR LEDs mounted symmetrically adjacent to the camera produce reliable reflections on the cornea. We use them to define the camera-axis reference position. By annotating frames in which both reflections were visible, we identified the pupil location corresponding to the camera axis for each eye and used this as the zero-angle reference for subsequent calculation of the eye vector relative to the camera as described above."

We did not find a way to utilize the LED position for estimating gaze-in-world vectors. Because the LEDs are rigidly attached to the headmount, their reflections remain within the egocentric reference frame. While we use the LED position to calibrate gaze-in-head-coordinates, we could not find a way to use them for gaze-in-world coordinates. In our view, this would require additional measurement of head pose relative to the environment.

Comment #6: fig 2b: Can you please add some anatomical directional indicators (e.g. nasal/temporal or something similar)? Also, could indicate the interest binocular conditions described in the text (line 32-33) to help readers?

To clarify directions in the figures, nasal directional markers were added to Fig 2B and Fig 3A, and text denoting the different binocular pairing conditions was added to Fig 2B top.

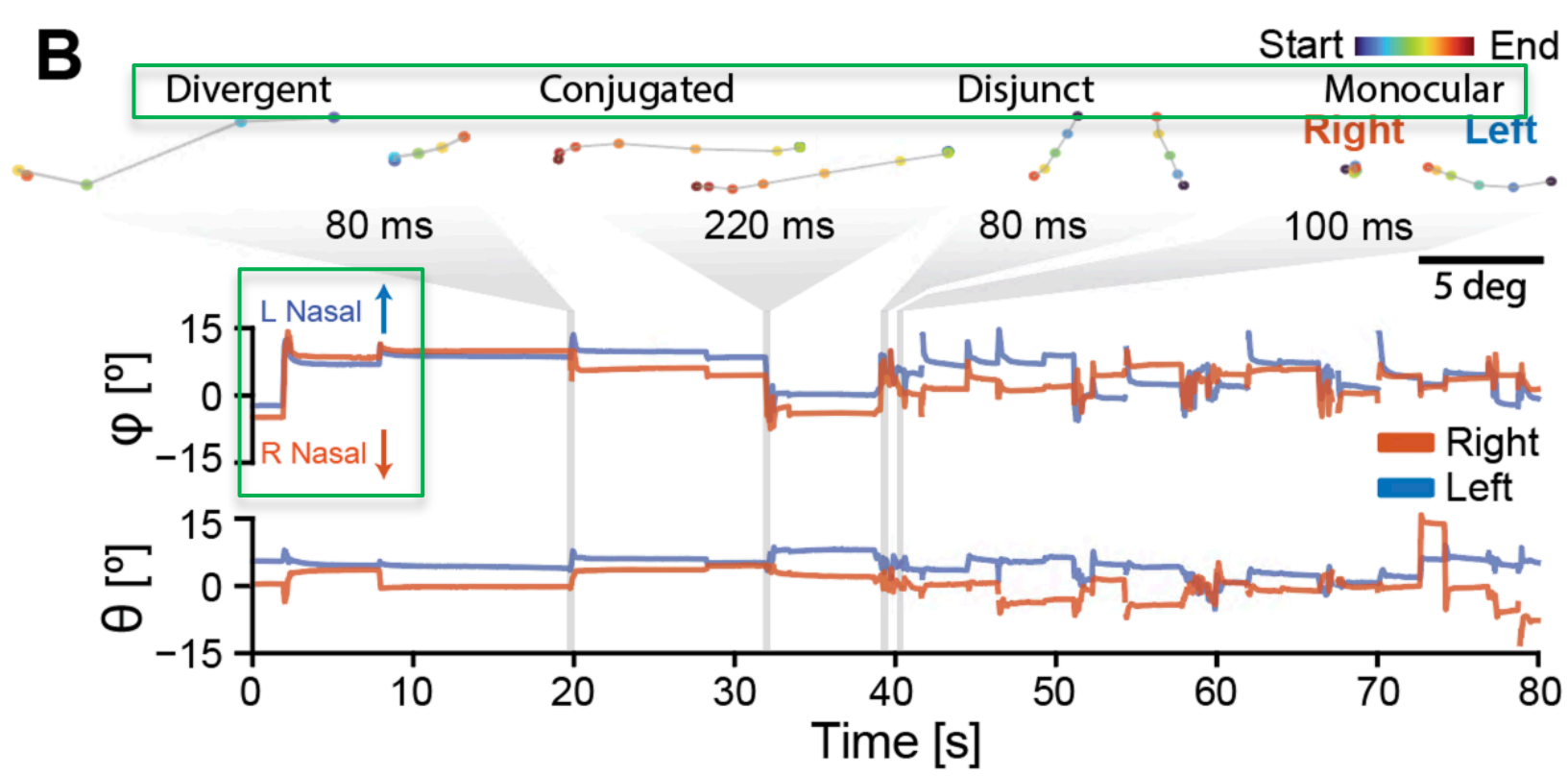

Comment #7: - fig 3a-b: x axes in seconds?

Thank you for noticing! Correct label was added to Fig 3A and 3B x-axis.

Comment #8: - Can you provide more details of how quiet/active periods were assessed? What happens if only a single 1 second window is labeled active in a long period of quiet? How long are active periods (they appear shorter than quiet ones)?

We expanded the behavioral-state annotation description in the Methods. We also added a separate analysis to assess Active and Quiet epoch distributions. To avoid short interruptions affecting continuous state annotations, we ignored short interruptions like the reviewer described. We termed these the 'sustained' Active and Quiet epoch durations, and plot their distribution (Fig S11B):

"Movement events were also converted into a continuous behavioral annotation by applying a rolling-window analysis (10-s window, 1-s step). For each window, the mean movement magnitude was computed, and windows were labeled as active when the mean exceeded a manually chosen threshold (visually chosen and verified by comparing accelerometer traces with arena videos of animal behavior) and quiet otherwise. Consecutive windows of matching labels were merged into one behavioral epoch. **These annotations were used without temporal smoothing for all state-dependent analyses in Fig 3, thus all epochs with length ≥ 1 s were included in the analysis.** To quantify the duration of sustained behavioral states **(Fig S11B),** we additionally generated a smoothed annotation in which opposite-state interruptions shorter than 3 s were bridged and resulting epochs shorter than 5 s were excluded. The median lengths of these smooth states were 15 s for Active epochs (IQR = 10-21 s) and 59 s for Quiet epochs (IQR = 32-170 s)"

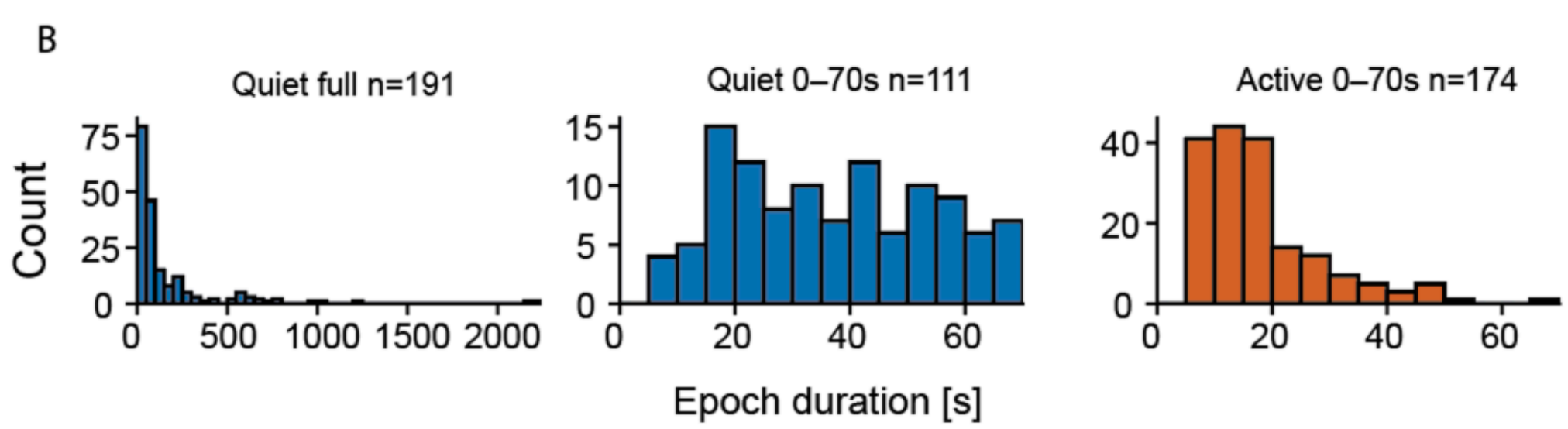

Figure S11B caption: (B) Distributions of sustained Active and Quiet epoch durations after bridging opposite-state interruptions shorter than 3 s and retaining epochs ≥5 s. Quiet-epoch durations (blue) are shown at full range (left), and on the same expanded timescale as Active epochs (right) (5-s bins). No active epochs are longer than 70 seconds.

Reviewer #2

The manuscript by Leberstein and colleagues introduces a personalized eye tracking system as a versatile method to study eye movements in freely moving animals. Using a combination of photogrammetry, 3D-modelling and -printing, the authors produce head mounts adaptable for diverse head-eye geometries and small vertebrate species (here: mouse, turtle, and lizard). The authors then use this system to provide a systematic characterization of eye movements in the lizard Pogona vitticeps. Stable, high-resolution recordings of eye movements in freely moving animals are technically challenging, yet can provide critical information for (neuro)biological studies. A system that is easily adaptable to different species, reliable, and accessible to a broad

scientific community, would represent an important contribution, and I am excited by the prospect of seeing this method being adopted and used in many labs and for studies in different species.
First of all, I want to applaud the authors for the quantitative analysis of eye movements in Pogona vitticeps, which I found both interesting and well executed, also representing a major part of the manuscript.

We thank the reviewer for sharing our excitement about the potential and importance of reliable eye tracking in freely behaving animals across species, and for defining it an “important contribution”. We also thank them for finding our analysis in *P. Vitticeps* “interesting and well executed”.

That said, I have been tasked to review this manuscript as a "Methods and Resources" article, and my evaluation thus focuses on the methodological approach, its novelty and potential to ask previously inaccessible biological questions, and to qualitatively compare it to existing systems. With this in mind, I am not yet convinced, and even though the method may well meet the necessary criteria, it needs to be demonstrated more clearly. However, I do suspect the necessary data may be there already, at least in part, or can be collected within a reasonable timeframe for a revised manuscript.

Comment #1: Specifically, one main strength of the approach would be its promised versatility and flexibility. The authors show images of three species carrying the head mount system, and they suggest they have successfully recorded eye movements in all three (that is, M. musculus, T. scripta elegans, and P. vitticeps). But that hasn't been shown, it is unclear what "successful" means, and how the data looks like and compares to that obtained with previously published methods.

To address these comments, we performed the following revisions: 1) We expanded the Results to define and demonstrate what “successful” means. Specifically, we quantified the eye image quality and reported the duration over which viable recordings were obtained. 2) We provided these statistics for all three animal models; 3) We added Fig S8 showing how the data looks like for all three models (see our response to comment #2); 4) To compare with previously published methods, we added a comparison table with existing video oculography solutions (Table S1).
The Results now read:
“We define successful recording as the ability to acquire long (20-60 min) eye-tracking videos during unrestrained behavior, with low camera jitter and a large eye image relative to the camera frame (representative eye data traces are shown in Fig S8A). At 640x480 pixels resolution, the eye ROI occupied 61.8% of the frame diagonal in lizards, 40.1% in mice, and 39.1% in turtles (mean manually annotated ROI diagonals: 495, 321, and 313 pixels, respectively). Recordings were obtained repeatedly over weeks to months from individual animals, typically once every 2-4 days, with viable recording periods of up to six months in *P. vitticeps*, two months in *T. scripta elegans*, and four weeks in mice. We compare system technical specifications against previously published video oculography works in Table S1”

Publication / system	This study	Wallace et al. 2013	Meyer et al. 2018	Michaiel et al. 2020	Sattler & Wehr 2021	Huang et al. 2022	Singh et al. 2025
**Species / cross-species implementation**	Lizard; turtle; mouse (3 species demonstrated)	Rat (1 species)	Mouse (1 species)	Mouse (1 species)	Mouse (1 species)	Mouse (1 species; broader small-animal adaptability proposed)	Marmoset (1 species; ~300–400 g)
**Reported carried mass**	Mouse: 3.5 g; lizard/turtle: ~5 g	~0.8 g per eye camera + ~0.5 g shared housing	~1.3 g single-camera system	2.6 g bilateral cameras + optics/holders + IMU	0.68 g 2-camera; 1.64 g 4-camera assembly	0.10 g single Sch1 ETU; up to 1.01 g one-eye Sch2	~20 g headpiece + ~40 g backpack
**Eye-video acquisition**	640 × 480; 60 Hz	752 × 480; 40–50 Hz	640 × 480; 60 Hz	480 × 640; 30-Hz interlaced → 60 fields/s	800 × 480 optical; 30-Hz interlaced → 60 fields/s	1280 × 720; 30 Hz	HD eye camera (pixel dimensions not reported); 90 fps
**Bilateral eye recording**	Yes	Yes	Optional / demonstrated	Yes	Yes (2-camera configuration)	Yes (two ETUs possible)	No (one eye camera)
**Head-motion measurement**	Motion sensor (3-axis accelerometer)	World-referenced 3D head pose (6 head LEDs + overhead cameras)	Motion sensor (accelerometer; gyroscope used in extended analyses)	Motion sensor (accelerometer + gyroscope IMU)	Motion sensor (headstage accelerometers)	Not intrinsic to ETU	Motion sensor (IMU)
**Eye-position extraction**	DLC pupil-edge landmarks → ellipse fit	Gradient-based pupil-boundary detection / ellipse representation	Pupil identified from registered eye images; pupil position/shape extracted computationally	DeepLabCut: 8 pupil-edge points → least-squares ellipse	System paper demonstrates eye/iris feature tracking; extraction method is application-dependent rather than a single prescribed pupil pipeline	Image segmentation + DeepLabCut tracking fused with adaptive Kalman filtering	UNet segmentation of pupil/iris from eye-camera images
**Eye-angle reconstruction & validation***	Wallace-derived geometric reconstruction from pupil ellipse/camera geometry; independently validated on this dataset: relative eye-in-head reprojection residuals φ 0.84 ± 0.48°, θ 1.19 ± 0.48°	Geometric 3D eye/camera model based on ellipse deformation; model-eye rotational detection error 0.9 ± 0.1°	No calibrated angular reconstruction or ground-truth angular validation reported	Explicitly follows Wallace et al. 2013: affine/geometric conversion of pupil ellipse to angular displacement. No ground truth; reported 0.51 ± 0.25° is an upper-bound stability/error estimate during stationary epochs	Cyclotorsion quantified in degrees; no calibrated horizontal/vertical pupil-to-eye-angle reconstruction or validation reported	Tracks pupil/eye position, but no directly comparable Wallace-style angular reconstruction validation or angular ground-truth accuracy metric identified	Pupil position mapped to screen/world coordinates by per-animal calibration (horizontal/vertical scale + offset). Head-fixed RMS stability 0.05°; freely moving median gaze error <1°
**Mechanical stability characterized**	Yes: image-plane displacement distributions (overall displacement compared with recording beginning) (Fig S9)	Yes: camera displacement estimated from anatomical eye landmarks and corrected; no directly comparable physical jitter statistic	Yes: mean inter-frame registered image motion <4 µm freely moving; <1 µm head-fixed	Yes: RMS frame-to-frame camera shake 0.49 ± 0.33 px	No quantitative jitter metric identified	No directly comparable jitter metric identified	No directly comparable mechanical-jitter metric identified
**Recording longevity / repeated-use window**	Up to ~6 months (lizard); ~2 months (turtle); ~4 weeks (mouse)	Not reported	Not reported	Not reported	Not reported	Not reported	Repeated sessions demonstrated; maximum viable system lifetime not reported as a comparable metric
**Eye-in-image / usable eye coverage**	Quantified across species (eye ROI occupancy	Not reported as an equivalent eye-in-	Not reported as an equivalent eye-in-frame	Not reported as an equivalent eye-in-frame	Not reported as an equivalent eye-in-frame	Not reported as an equivalent	Not reported as an equivalent

	reported relative to frame dimensions)	frame occupancy metric	occupancy metric	occupancy metric	occupancy metric	eye-in-frame occupancy metric	eye-in-frame occupancy metric
**Individual-anatomy-derived headmount geometry**	Yes: rigid workflow uses individual photogrammetry	No: camera positioned using adjustable ball joint	No: generic printed holder; mirror adjusted in situ	No: connectors positioned/fixed during fitting	No: standardized printed carriers; position/focus adjusted as needed	No: connector position adapted during fitting	No: custom-fitted/adjusted per animal, but not derived from 3D anatomical reconstruction
**Predefined rigid camera–eye geometry**	Yes (rigid); No (modular)	No: camera position adjusted to eye	Partial: holder fixed; mirror adjusted then permanently fixed	Partial: alignment established during initial fitting then fixed	Yes for fixed 4-camera carrier; adjustable 2-camera variant also available	No: detachable ETU position adjusted through connector placement	Partial: repeatable after per-animal fitting to two headposts
**Multiple system configurations**	Individualized photogrammetric rigid headmount + modular in-situ-fitted headmount; bilateral configurations	Bilateral eye-camera system with adjustable camera positioning	One- or two-camera implementations; adjustable mirror/camera geometry	Primarily bilateral: two eye cameras + IMU; alignment fixed after fitting	Fixed 4-camera multimodal carrier + adjustable 2-camera headset	One or two detachable ETUs; direct-view (Sch1) and NIR-reflector (Sch2) configurations	Single eye + world-camera headpiece; components custom-fitted to animal
**Open hardware / design resources**	Yes	Detailed supplementary construction/calibration methods; no dedicated open source repository + highly specialized design	Yes: STL + acquisition resources	Partial: analysis/data resources; no equivalent complete open hardware package	Yes: detailed build + printable files	Yes: code/tracking resources; hardware construction described	Yes: headpiece STL + pupil-tracking GUI/code

"Table S1. Comparison with previously published video-oculography systems for eye tracking in freely moving animals. Our system was compared with the following systems: Wallace et al. (2013) [25], Meyer et al. (2018) [26], Michaiel et al. (2020) [34], Sattler and Wehr (2021) [27], Huang et al. (2022) [30], and Singh et al. (2025) [96]. In summary, our system has technical specifications comparable with the state-of-the-art while demonstrating a personalized reproducible approach that is adaptable to diverse head-eye geometries, thus allowing eye tracking across species."

To demonstrate the versatility of the system, we revised the Results text to clearly discuss how we further tuned it to get better recordings from mice:

"To accommodate the smaller size and weight of mice, we further miniaturized the headmount to 3.5 g and replaced the brass hexagonal anchor with a lightweight plastic version. To enlarge the area of the smaller mouse eye on the sensor, we used longer-focal-length lenses (4.5 mm) with a shorter minimum working distance, producing a narrower field of view and a larger eye image within the camera frame (Fig S8A).

Comment #2: The images in Fig 1F don't tell us much, and all other figures and data are specific to Pogona. To allow for a comparison with existing methods, and to actually demonstrate the system's versatility and usefulness to the broader scientific community, it is essential to provide additional analyses at least for the mouse, and hence a widely used model for which various other systems have been introduced in the past.

Moreover, though perhaps less critical in this context, this would also permit a direct comparison of visual acquisition strategies in a reptile and a mammal, using the same (novel) approach, strengthening the author's scientific conclusions and interpretation of their data.

To expand on Fig 1F and demonstrate the usefulness of our system for other animals models we added a new supplementary figure (Fig S8). This figure includes representative reconstructed eye-position traces from all three species and a quantitative analysis of mouse recordings highlighting similarities and differences between mouse and lizard. While a more detailed interspecies comparison would be extremely interesting, and we are currently working on such a comparison with collaborators, it is beyond the scope of this Methods paper (as noted by the reviewer).

We revised the text to discuss this as mentioned in Comment #1, and additionally in this point of the Results:
"Across animals, peak angular speed scaled systematically with saccade amplitude, forming a linear relationship across most of the measured range, with a tendency toward saturation at higher amplitudes (>20°) (Fig 2E). The relationship was similar for monocular and concurrent saccades (Fig S13), **and to the one we observed in mice (Fig S8B-S8D)."**

And these points of the Discussion:
"Individual eye movements in *P. vitticeps* showed the characteristic rapid acceleration and deceleration phases that define vertebrate saccades [38,76] (Fig 2C and 2D), and their peak speed scaled linearly with amplitude (Fig 2E). **We observed the same regime in our mouse recordings (Fig S8B-S8D).** This relationship largely resembles the 'main sequence' previously described in primates, cats, and mice [77–80] with a similar rate slope of ~20 [1/s] in both primates [79] and *P. vitticeps* (Fig 2E). "

"However, this potential is not uniformly expressed across taxa: many lateral-eyed mammals and birds exhibit tightly yoked ocular rotations [29,36]. **Our analysis of mouse eye movements agrees with this pattern, with saccades exhibiting coupled ocular dynamics (Fig S8E).** Against this backdrop, we observed flexible ocular coupling in *P. vitticeps* (Fig 2A, 2F and 2G)."

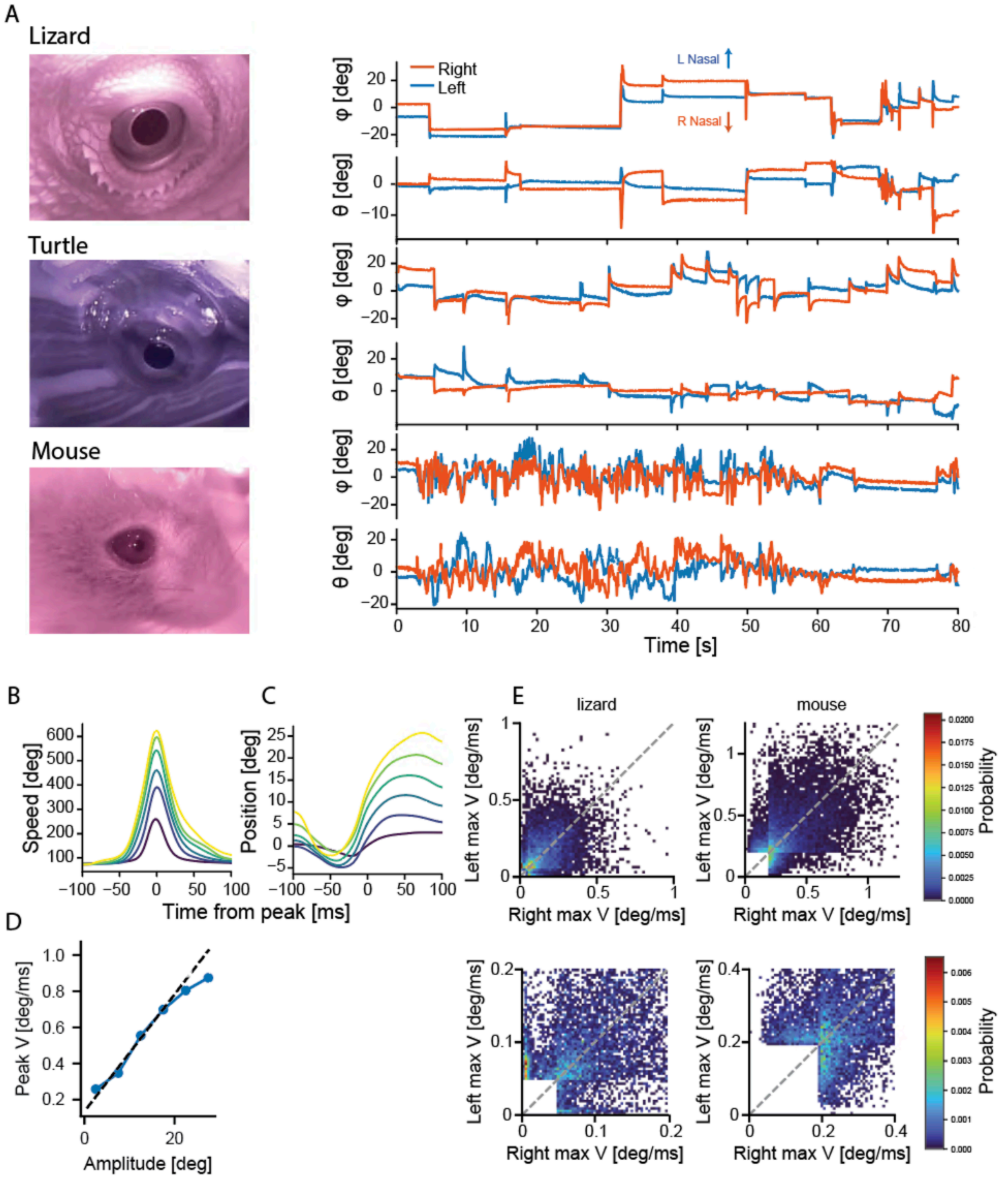

Fig S8. Cross-species eye tracking and quantitative characterization of mouse eye movements.

(A) Representative eye-video frames and corresponding horizontal (φ) and vertical (θ) eye-position traces from lizard (*P. vitticeps*), mouse (*M. musculus*), and turtle (*T. scripta elegans*). All traces are zeroed to animal rest, computed as the median eye position across each recording, for

each eye. **(B)** Mouse saccade angular-speed profiles aligned to peak speed. Other same-eye saccades occurring within the ±100-ms window were masked. The dashed line indicates the mouse saccade-detection threshold (3.23°/frame). **(C)** Mouse eye-position profiles corresponding to the isolated saccades shown in (B). **(D)** Relationship between saccade amplitude and peak angular speed in mice. Amplitude was calculated from onset to offset, or from the pre-onset position to offset for single-frame events, consistent with Fig S13. **(E)** Interocular peak-speed coupling for all detected saccades in *P. vitticeps* (left; detection threshold 0.8°/frame) and *M. musculus* (right; 3.23°/frame). The same relative subset and histogram binning are used for both species. Because head movement annotations were unavailable for mice, the lizard data are shown without the head-stationary filtering used in Fig 2F.

Comment #3: Another important aspect is the optional use of a "modular solution kit", which Leberstein and colleagues introduce as an alternative by which the fitting procedure can be performed directly on the animal. This would represent a major advantage, because photogrammetry may indeed often not be an option, and a modular solution would make the whole method much more accessible, certainly for people working with common rodent models, but also for others. To demonstrate this, it would be essential for the authors to provide some direct comparison with their rigid system in terms of stability and data quality/noise etc. (Note: By analyzing the existing mouse data, the authors could probably do much of it in one go, as they have collected these with the modular system; though ideally one could also show a direct comparison within the same species, such as the lizard).

To directly evaluate the modular solution kit, we expanded the camera-stability analysis to compare recordings obtained using modular headmounts in all three species. We also compared the modular headmount to the single-piece headmount for lizards in Fig S9. The Methods now state:

"To compare mechanical stability across headmount configurations, this analysis was applied to recordings obtained using modular headmounts in mice, turtles, and lizards, as well as personalized single-piece headmounts in lizards. We found that in all configurations the median jitter amplitude was ≤ 90µm - typically 1-2-pixels. Within lizards, the single-piece headmounts were characterized by smaller jitter (Fig. S9) suggesting it is the optimal solution when applicable."

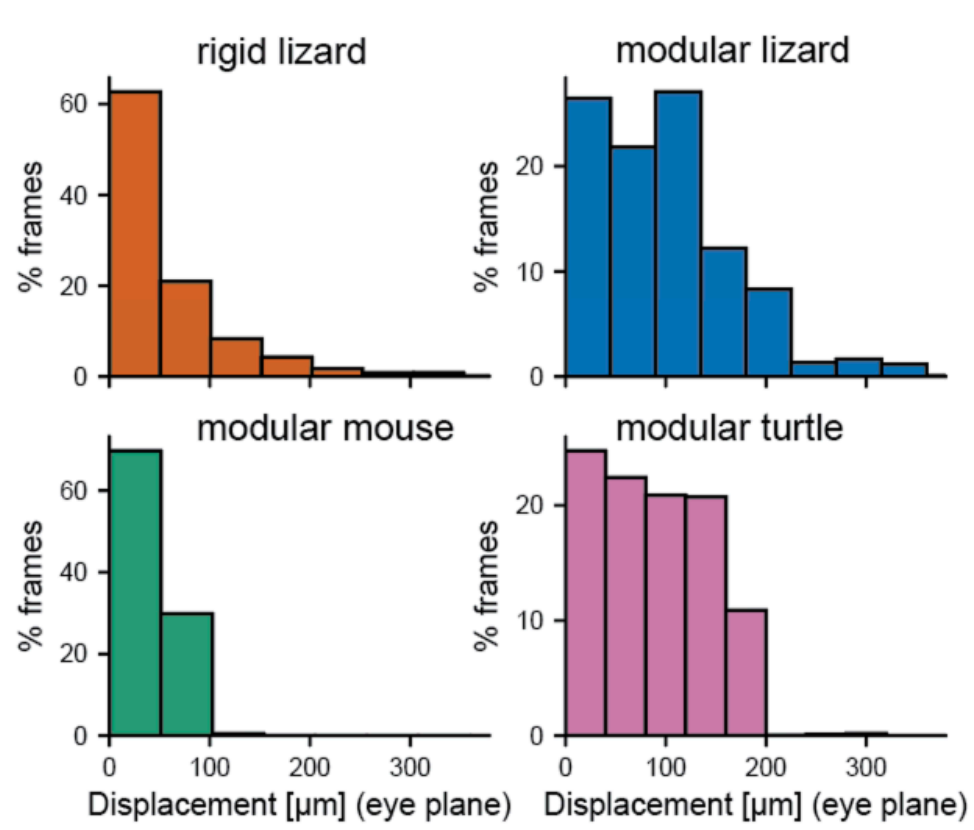

Fig S9. Camera jitter at the imaged eye plane. Displacement histograms for rigid (single-piece) lizard and modular lizard / mouse / turtle mounts. Medians: mouse = 28 μm (2.2 px), turtle = 80 μm (2.0 px), modular lizard = 90 μm (2.0 px), rigid lizard = 42 μm (1.0 px). 95th percentiles: mouse = 90 μm (7.1 px), turtle = 179 μm (4.5 px), modular lizard = 211 μm (5.0 px), rigid lizard = 173 μm (5.4 px).

Minor comments:
Comment #4: Comparing a mouse and lizard species does of course not allow for conclusions about possibly general differences in visual acquisition strategies between ecto- and endotherms. This would require a much broader sampling, and the authors should hence tone down their conclusions/motivation to make this clear.
But, the Pogona data provides a first hint such differences might exist, which is interesting. Along those lines, adding data from the turtle could help in this respect, provided it matches the overall patterns observed in the distantly related lizard species (i.e. it could suggest a more general reptilian phenotype).

To address the reviewer's comment we revised the text in the abstract, introduction and discussion sections to restrict our conclusions explicitly to *P. vitticeps*
The Abstract now reads):
"Visual acquisition strategies were strongly tied to behavioral states with periods of active sampling (3-5Hz saccades) separated by periods of ocular stillness lasting many seconds and suggesting a visual acquisition strategy distinct from those commonly described in endothermic model species."

The introduction now reads:
"Next, focusing on *P. vitticeps*, a foveated visually orienting, laterally eyed agamid lizard [47,55], we address key questions in visual acquisition. We ask whether agamid lizards exhibit visually guided ocular saccades. We examine whether visual acquisition strategies in *P. vitticeps* differ from patterns described in endotherms across long time scales. We probe the relation between pupil diameter and behavioral states. Studying these questions revealed a complex pattern of visual gaze shifts and lateralized behaviors, as well as state-dependent changes in visual acquisition and pupil diameter in *P. vitticeps*."

The discussion now was changed in two locations and now reads:
"The temporal structure of eye movements in *P. vitticeps* revealed a striking departure from patterns described in endothermic vertebrates. Lizards often remained without ocular motion for several tens of seconds, intervals far longer than the continuous high-frequency sampling typical of endothermic vertebrates, including birds [94], rodents [36,95], and primates [16,62,96,97]. In *P. vitticeps*, these prolonged quiet states alternated with relatively brief epochs of dense saccadic activity (active states), producing a bimodal distribution of inter-saccadic intervals (Fig 3). Whether this temporal organization represents a broader reptilian or ectothermic visual acquisition strategy will require comparative measurements across additional species."

"Our results demonstrate how expanding eye-tracking to reptiles can facilitate the extraction of conserved acquisition strategies (e.g. the saccadic main sequence and axial biases in scanning) as well as specializations (e.g. *P. vitticeps's* acquisition mode and modulation of pupil diameter by behavioral state)."

Comment #5: It wasn't clear to me what the difference is between Fig. 2f and S3 (left panel). In both cases, are we looking at inter-ocular peak-speed coupling? If so, is S3 simply a zoomed-in version?

To clarify the ambiguity in the captions they were both revised as follows:
The Fig 2 caption now reads"**(F)** Inter-ocular peak-speed coupling for **monocular, head stationary saccades.** Two-dimensional probability histogram of right- vs left-eye peak angular speed [°/ms] for monocular saccades during head-stationary epochs pooled across animals (equal-animal weighting)"

"**Fig S3. Coupling of peak saccade speed between eyes, with and without head movements.** Two-dimensional histograms show the joint distribution of right- and left-eye peak saccade speed (peak S; °/ms) for monocular and binocular saccades occurring in the absence of head movements (left) and with head movements (right), plotted over 0–0.2 °/ms with **100 × 100 bins**. Each point contributing to the histogram corresponds to a saccade detected in one eye, paired with the maximum angular speed in the contralateral eye within a ±51 ms window around saccade onset. Histograms were pooled between animals for equal contribution. The dashed line indicates equality between eyes. Color denotes probability per bin (each panel independently normalized to sum to 1; color scale shared between these two panels only, not with Fig 2F)."

Comment #6: I am probably missing something, but why does the density of the points seem to differ, and why do the probabilities differ by a factor of 10 (colour bar)?

Fig S3 is not a zoomed-in version of Fig 2F: Fig 2F is restricted to monocular saccades during head-stationary epochs, whereas Fig S3 includes both monocular and binocular saccades and compares head-stationary and head-moving conditions. The difference in probability scales results primarily from the different histogram binning (100 × 100 bins in Fig. S3 versus 60 × 60 in Fig 2F), which distributes the probability across substantially more bins. We clarified this in the figure caption (See Comment #4) and edited the Methods text to reflect this difference:
"To complement the monocular head-static analysis in Fig 2F, Fig S3 shows a similar coupling for all head-static and head-moving saccades with increased resolution (100 x 100 bins vs 60 x 60 in Fig 2F)"

Comment #7: Fig. 3: Figure captions d to f were mixed up and don't match with the legend below.

We thank the reviewer for noticing. The legend was corrected.

Reviewer #3

In this study, the authors developed technological solutions for tracking eye movements in two dimensions for freely moving terrestrial vertebrates of varying head shapes. The authors then use this technique to characterize eye movements of the lizard P. vitticeps, especially saccades. Lizard saccades show similarity to human saccades in terms of speed profiles and speed-amplitude dependence ("main sequence"), which isn't entirely surprising, but a useful characterization. The authors then move on to show that lizard saccades (somewhat similar to the situation in the chameleon) show many monocular saccades (especially small-amplitude ones) as well as binocularly coordinated saccades. The monocularly coordinated saccades do occur in any direction ("isotropic"), while the binocularly coordinated saccades show movement mainly along one orientation (that likely corresponds to the horizontal plane). These findings are intriguing as they show that the lizard (unlike e.g. humans) readily performs monocular as well as binocular saccades, which is discussed to potentially be related to different proximal functions (sampling vs. reflexive gaze shifting). Finally, the authors add head acceleration measurement to identify quiet and active periods (locomotion/posture). Saccade rate is much higher during active periods and pupil size scales inversely with locomotor activity. These results show that the lizard eye movements during awake times follow different behavior states, which provides interesting links to related processes and sleep in other species. Given that lizards are ectothermic and eye movements and locomotor activity are related to energy consumption, the provided characterization is a valuable addition to the literature, as the results will inform further comparative studies on the functional role and evolutionary optimization of eye movements. The authors also discuss the observed eye movements and the situation in the lizard as an opportunity to study "attention" in an evolutionary ancient vertebrate. However, I do not find this claim very convincing in the absence of evidence for "attentive" behavior in this study.

The provided analysis is very sound and the statistical methods appear appropriate. This is a well-rounded study on eye movements in a non-canonical animal model that fills an important gap in the literature and furthermore provides technical solution examples for eye tracking that can likely be adapted to other animal species easily.
I also have a few more detail comments and criticisms, which I provide below.

We thank the reviewer for finding that our study “fills an important gap in the literature”, is “well-grounded“, and that our analysis is “very sound”, and our statistical methods “appropriate”. We are also happy to hear the reviewer recognizes the potential of our approach for expanding eye-tracking in freely moving animals to other species. We address the reviewer’s comment on attention as part of the more specific comments detailed below (Comment #12).

Major comments:
Comment #1: Analysis/Illustration caveat: In Figure 2f, why do conjugate saccades have such small amplitude (speed) compared to monocular ones (In the text you state the opposite)?

Following this comment, we realized that our description of Figure 2F was not sufficiently clear. The results in Fig 2F are restricted to events classified as monocular. Thus, the events near the

unity line should not be interpreted as the amplitude distribution of concurrent saccades. We clarified this in the caption and added a title to the plot (green rectangle):

"**F.** Inter-ocular peak-speed coupling for monocular, head stationary saccades. Two-dimensional probability histogram of right- vs left-eye peak angular speed [°/ms] for monocular saccades during head-stationary epochs pooled across animals (equal-animal weighting)"

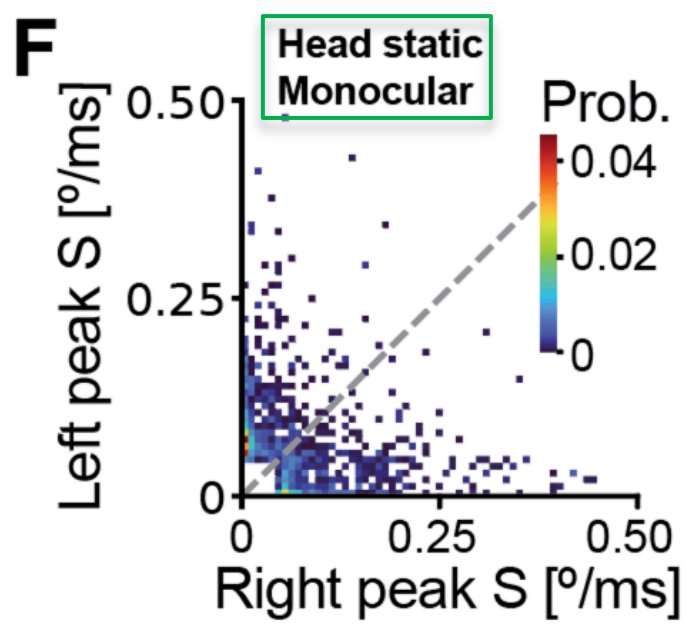

Comment #2: In conjunction with Figure 2g: why are monocular saccades so frequent (is it due to detection noise? -the label "concurrent" is less likely to occur for random data than the label "monocular", this could explain the high peak for small velocity monocular saccades)?

The two distributions in Fig 2G do not compare frequency of monocular vs concurrent saccades but rather show the amplitude distribution of each saccade type (monocular/ concurrent). Each distribution was independently normalized. To clarify this, we revised the caption to read:

"**G**. (Top) **Independently normalized** probability distributions of saccade amplitudes for concurrent (green) and monocular (blue) events. Mean (±S.E.M shading) animal-wise probability distributions (0-25°). (Bottom), Per-animal difference curves (concurrent - monocular)."

We have also revised the results text to read:

"We found monocular (highly asymmetric) saccades in all animals and across all recording sessions (Fig 2F, S3, and Video S2). Only 3.7% of monocular saccades were followed by a directionally aligned contralateral saccade within 80 ms, indicating that the vast majority were not subsequently corrected by the other eye. We next compared **the independently normalized** amplitude distributions of monocular and concurrent saccades. While small-amplitude saccades were more frequent than large-amplitude events in both distributions, monocular saccades were typically smaller than concurrent events (Fig 2G)."

Finally, we verify that small monocular events do not reflect the detection of noise events, we added a quantitative characterization of the eye tracking noise and saccade detection parameters in Fig S10. In addition, the "Spatiotemporal limitations" of saccade detection in our system are now elaborated in Methods (See response to Reviewer 1, Comment #3).

Comment #3: p.5, l. 7: it is stated that concurrent saccades are of larger amplitude, but Figure 2f in combination with 2d imply the opposite…much fewer large saccades along the line of unity are present.

Concurrent events are indeed of increased amplitudes relative to monocular (Fig 2G), but Fig 2F does not show this relationship as it only shows monocular saccades during head-static epochs. We clarified this in the revised manuscript (See response to Reviewer 3 Comment #1)

Comment #4: Do monocular and binocular saccades have the same main sequence?
In Fig 2e, it would be helpful to plot the individual saccade data points in the background.

Following the reviewer's suggestion, we added Fig S13 which includes a direct comparison of the main-sequence relationships of monocular and concurrent events (Fig S13A-S13C and S13E) and the corresponding individual saccade data (Fig S13D).
These figures are now referenced in the results:
"Across animals, peak angular speed scaled systematically with saccade amplitude, forming a linear relationship across most of the measured range, with a tendency toward saturation at higher amplitudes (>20°) (Fig 2E). The relationship was similar for monocular and concurrent saccades (Fig S13A-S13C), with monocular saccades showing reduced saturation at higher amplitudes and a slightly steeper slope (Fig S13E)"

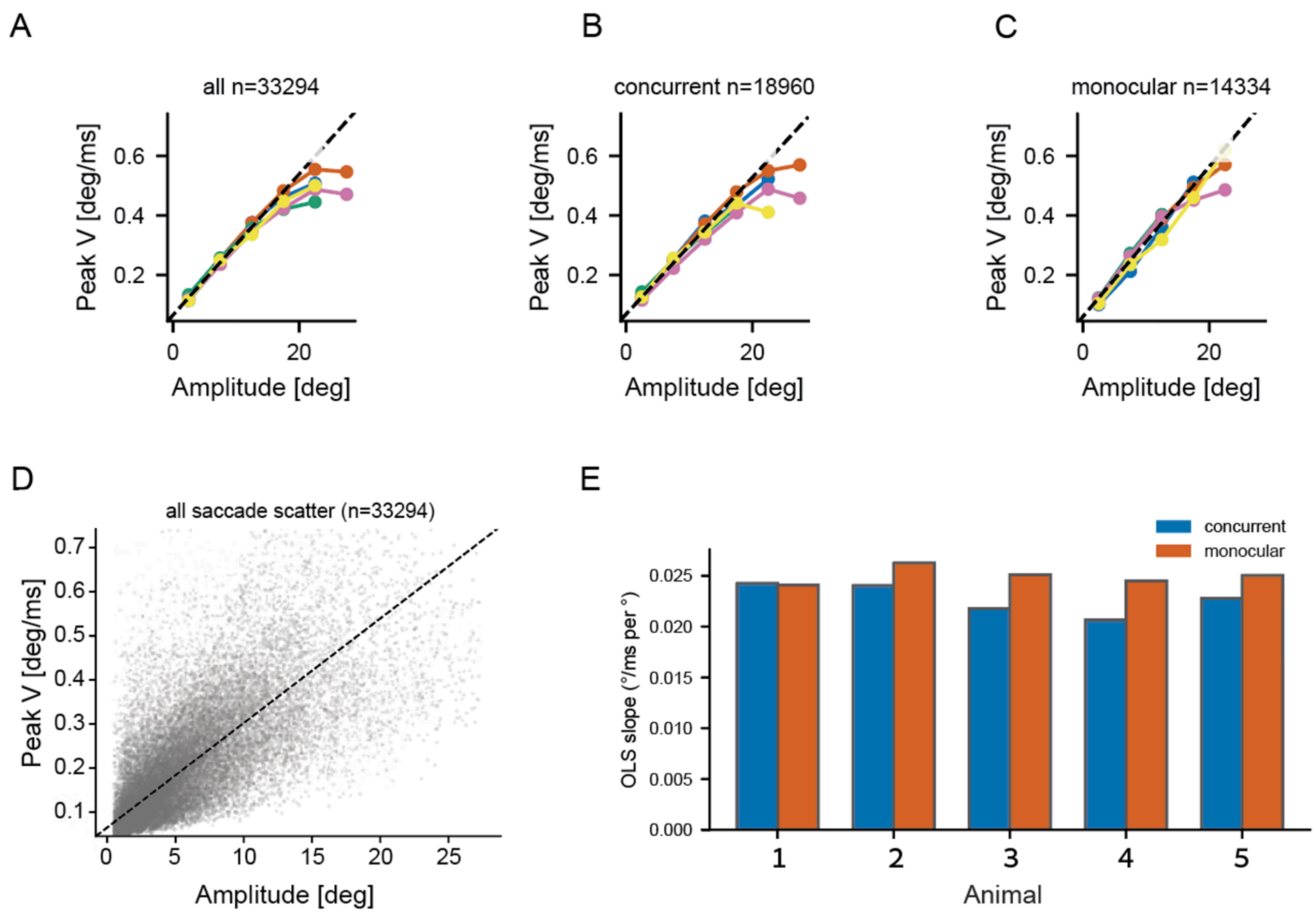

"Fig S13 Main-sequence relationships for all, concurrent, and monocular saccades.
(A-C) Mean peak angular speed as a function of saccade amplitude for all (A), concurrent (B), and monocular (C) saccades. Colored lines represent individual animals; dashed lines show the population linear fit. **(D)** Peak angular speed versus amplitude for all individual saccades pooled across animals (n = 33,294); dashed line shows the linear fit. Saccade amplitude was calculated

from onset to offset; for single-interframe-interval events, the pre-onset position was used as the starting position to capture the displacement associated with the detected velocity peak. **(E)** Comparison of per-animal ordinary least-squares (OLS) slopes for concurrent and monocular saccades. Monocular saccades exhibited a slightly steeper main-sequence relationship (concurrent: 0.0227 ± 0.0007; monocular: 0.0250 ± 0.0004 °/ms per degree, mean ± SEM; paired t-test: $t = -3.32$, $df = 4$, $p = 0.0294$; $n = 5$ animals)."

Comment #5: Results in Figure 1 are pitched as a general technical solution that works for many species. I believe that this is true, however, I'd appreciate if the authors put in more effort to describe the difficulties and important points of consideration in the main text. This would help future scientists to estimate whether the planned approach (for a new species) would likely work or not. For example, a) What are the limitations with regard to head shape for headmounts,

To clarify the constraints imposed by head shape and pupil contrast, we added the following text to Methods:
"Head shape itself primarily imposes species-specific design constraints on camera and mirror placement, which can be easily accommodated by the customizable geometry of the system. A stable headmount attachment point is a required prerequisite but should be possible for animals with a skull. Animals with a larger distance between the eyes and the headmount will require longer mirror holders, which if too long may result in vibrations and require thicker holders. Successful tracking requires sufficient pupil contrast. Although we did not quantify a minimum contrast threshold or explicit skull thickness thresholds, the system was successfully installed and used in mice, which had the thinnest skull and least distinct pupil boundary among the species tested here."

For limitations related to screw implantation see Comment #6 and for limitations related to the photogrammetry scanning (see response to Reviewer 1 Comment #2):

Comment #6: b) What type of skull (thicknesses) allow screw anchoring/implantation?

We address the species-specific anchoring procedures in the Methods:
"The possibility to implant screws in the skull is animal-specific. In reptiles and turtles, the anchor was reinforced using miniature cranial screws inserted to a depth of 0.4-0.5mm. Since in some animals the skull may not be thick enough to install screws, the anchor can be secured using adhesive and dental cement alone, as we demonstrated for the mouse."

Comment #7: c) Please describe, for how long the implants were stable and what the latest time points (hours/days after implantation) were for the successful recordings.

We now report the recording longevity obtained in each species in the Results:
"Recordings were obtained repeatedly over weeks to months from individual animals, typically once every 2-4 days, with viable recording periods of up to six months in *P. vitticeps*, two months in *T. scripta elegans*, and four weeks in *M. musculus*."

Comment #8: d) for what type of eye morphologies/pupil contrasts will this method work?

See response to Comment #5.

Comment #9: Angular eye position detection errors were likely up to 5° in size (p. 16, l26). This is important information and should be stated in the main text.

The 5° error is the total error of the weak perspective model but its effect on our results is smaller. We have clarified the error measure relevant to our relative eye-movement analyses and now report it in the main text and Fig S1B (see below):

"Eye videos were used to reconstruct egocentric gaze vectors (eye-in-head angles) from pupil annotations using a weak perspective model (Fig 2A) [25,33,35,36,59,60], validated using a 3D Blender-based [61] simulation of the eye-camera system (Methods, Fig S1A). Because head posture relative to the environment was not reconstructed, our analyses quantify relative eye-in-head positions rather than absolute gaze direction in world coordinates. We therefore used the 3D simulation to quantify the relative eye-in-head estimation errors (|Δ|) in φ (mean= 0.84 ± 0.48°, range 0.42–1.67°, std= 0.95 ± 0.42°) and θ (mean = 1.19 ± 0.48°, range 0.65–1.93°, std = 1.18 ± 0.51°) (Methods, Fig S1B). "

The Methods text now describes these calculations:

"Because subsequent analyses quantified eye movements relative to each eye's resting position, we additionally assessed the effect of reprojection error on relative angular measurements. Raw and corrected eye-position estimates were independently zeroed to their respective resting positions, and the residual difference between these rest-centered datasets was used to quantify relative gaze angle error (Fig. S1B)."

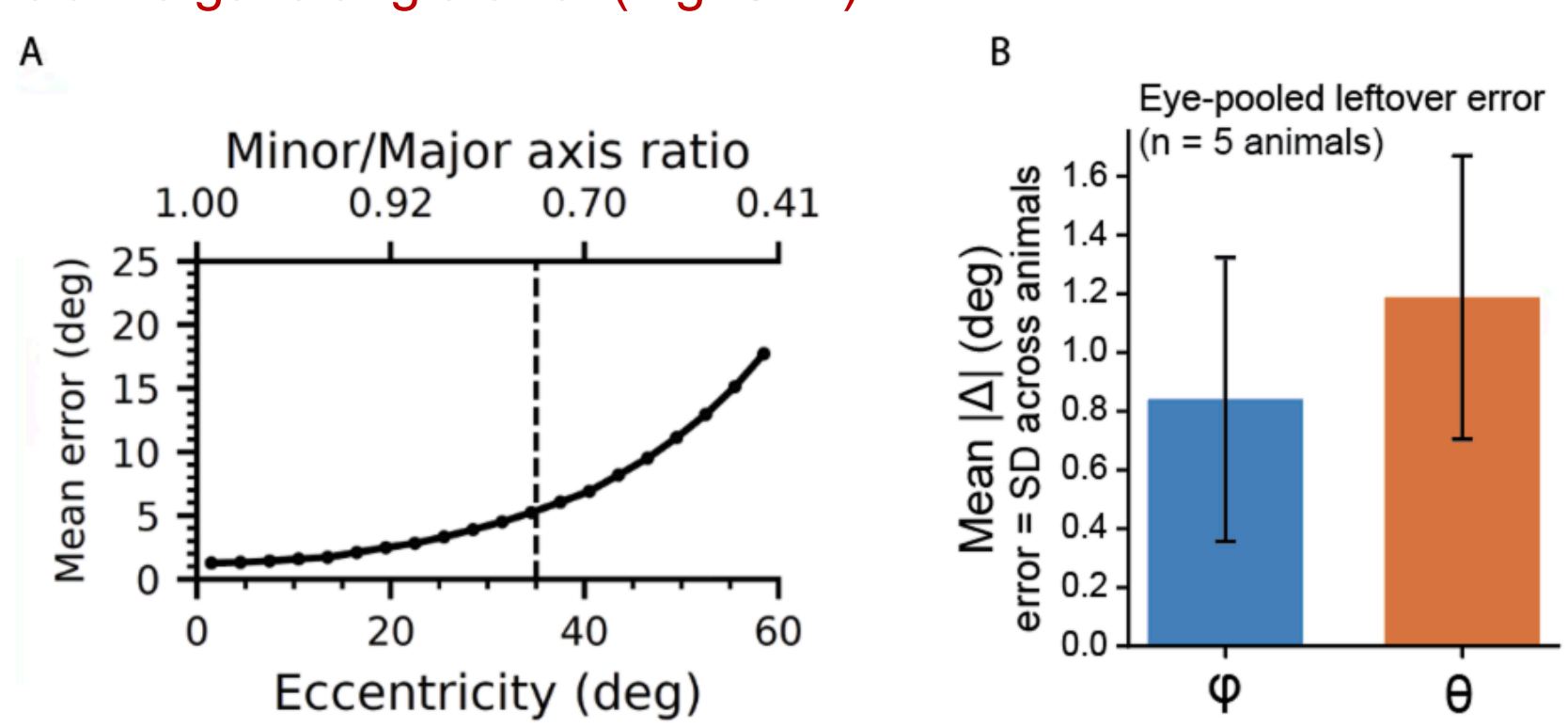

Fig S1. Validation of angular eye-position reconstruction.

(A) Simulation-based assessment of angular reconstruction accuracy. A rendered 3D eye was rotated through known azimuth (φ) and elevation (θ) angles from −60° to +60° in 1° increments, and reconstructed eye positions were compared with the ground truth. Ellipse eccentricity (minor/major axis ratio; top) and mean total angular error, $\sqrt{[(\Delta\varphi)^2 + (\Delta\theta)^2]}$, as a function of gaze eccentricity (bottom) are shown. The dashed line indicates the extended *P. vitticeps* oculomotor range (±35°) **(B)** Eye-pooled relative reprojection error after independently centering the raw and corrected eye-position estimates on their respective resting positions. Residual absolute errors

were φ: 0.84 ± 0.48° (range: 0.42–1.67°; mean ± SD across animals; n = 5) and θ: 1.19 ± 0.48° (range: 0.65–1.93°; n = 5).

Comment #10: What fraction/types of detected saccades are likely artifacts or behavior not related to an intended eye position change? E.g. in the video S1, the saccade is sometimes followed (within milliseconds) by a saccade back to the original starting position. Are these "jerks", in which eye movements result from the animal trying to do something else than moving its eyes? If not, what could be the function of such eye movements?

The threshold for saccades was set to minimize the detection of small artifact saccades. In the revised manuscript we added noise analysis and discuss the spatiotemporal limitations in Methods (see Reviewer 1 Comment #3).
The function of eye movements in video S1 is indeed an interesting question. However, mapping the full repertoire of eye movements in *P. Vitticeps* and across species is a challenge. Further, to understand their function, we are currently conducting experiments under different visual stimulation conditions and during defined visual tasks. However, this investigation is beyond the scope of the current publication. Here, we focus on introducing our experimental system and demonstrated its biological potential in a lizard. We aim to perform an exhaustive characterization of eye movements and their function in future works.

Comment #11: Is eye tracking stable during locomotion or do eye position artifacts occur (e.g. due to wobble of the hardware assembly)?

We quantified the stability of the system and presented it in Fig. 1E. Importantly, this analysis was performed during both static epochs and periods of locomotion. We clarify this in the Results which now read:
“To assess the stability of the system, we quantified camera jitter (spatial cross-correlation peak shift between the first frame and other frames calculated on a stationary image segment) during free behavior **(which included locomotion)**. The typical measured jitter was small (overall span 0–200μm at the imaged eye plane with a median of 42μm (~1 pixel) for rigid lizard headmounts) (Fig 1E and S9) and could be corrected by subtracting the jitter amplitude.”

In addition, we excluded a small minority of events (constituting 0.21% of samples) with above threshold jitter values. We clarify this in the methods:
“To limit eye-position artifacts caused by movement of the camera assembly relative to the eye, samples exhibiting >5-pixels interframe image displacement were excluded from subsequent eye-movement analysis. This criterion excluded 0.21% of samples across the dataset.”

Finally, in the revised manuscript, we added the stability analysis to all 4 headmount applications across species (Fig S9, see response to Reviewer 2 Comment #3).

Comment #12: p.7 l.35: "attention" research possible in P. vitticeps: This is an interesting possibility. However, the authors did not show that the saccades in P. vitticeps are goal-directed or "voluntary" and it seems possible that they are "spontaneous" scanning saccades. Therefore,

the relation of the observed eye movements to "attention" is not straightforward. This should be discussed more carefully or toned down.

We appreciate this distinction and agree that our data do not establish whether individual saccades are voluntary, goal-directed, or associated with shifts of attention. We therefore revised the Results section to remove interpretations of these movements as direct evidence of attention and instead describe them in terms of visual sampling and gaze redirection:
“Thus, monocular saccades are fast and local while concurrent saccades are larger, possibly reflecting gaze shifts during locomotion or **broader visual sampling rather than local detail extraction.**”

“Thus, while most gaze shifts occur during movement and play a role in gaze stabilization, a considerable fraction of eye-led monocular (and binocular) gaze shifts occur during stillness, demonstrating that **these movements are not exclusively coupled to head movement or gaze stabilization.**”

We also revised the Discussion in two locations to distinguish these observations from conclusions regarding attention:
“By contrast, *P. vitticeps* frequently generated saccades in the absence of head movements, consistent with eye-led reorienting similarly to primates. These events likely reflect **visually guided ocular shifts**, and are supported by evidence for a fovea in agamid lizards [47]. We note however, that foveated animals can perform visually guided behaviors also using head movements.”
“In primates, the existence of directed, time-stamped saccades have been widely leveraged to study attention and decision-making [5,92]. The presence of eye-led gaze shifts in *P. vitticeps* suggests a tractable route for extending visual attention studies to reptiles, thus providing a comparative perspective that can shed light on the evolution of attention and corresponding neural processing [93]”

Also, the title sounds a bit overstated to the ears of the reviewer ("complex state-dependent visual acquisition"). To uphold this claim, the authors should show that the different types of eye movements are task-associated, e.g. by showing an intricate relation of eye movements and visual stimuli and/or locomotion.

While we agree with the reviewer’s perspective about attention, in our view, the current phrasing of the title does not claim task-associated eye movements. By “complex” we refer to the diverse oculomotor repertoire observed in *P. vitticeps*, including monocular and binocular saccades, the spatial profiles of both types, their amplitude distribution, and their directional biases, as well as their link to head movement. By “State-dependent” we refer to the measured differences in saccade frequency, temporal organization, and pupil diameter between Active and Quiet movement states.

Comment #13: In the video S1 the reviewer observed a nystagmus-like eye movement (eye moves back-and-forth a couple of times with small amplitude) right after the saccade in a couple

of cases. How frequently did this occur and what is a putative purpose of such eye movement? For primates, the eye position would rather remain stable directly after the saccade.

We thank the reviewer for this interesting observation. We quantified these post-saccadic back-and-forth movements and added the analysis to Fig. S12, together with the corresponding Results, Discussion and Methods descriptions.

The Results now read:
"Post-saccadic back-and-forth sequences were also observed following 21.3% of large (≥7°) saccades during head movement (639/2997 events; Fig S12)."

The Discussion now reads:
"In many lateral-eyed mammals such as rodents and ungulates, saccades are reported primarily as quick phases supporting head-led gaze shifts and vestibular correction [36,38]. These animals rely predominantly on head rotations to redirect gaze with minor oculomotor span (~20° for mice [36]). This behavior fits the lack of a clear localized fovea or the existence of a visual streak [81–83]. **Similarly, we observed nystagmus-like post-saccadic back-and-forth movements in a subset of large saccades occurring during head movements, consistent with a compensatory role in gaze stabilization during head movement (Fig S12)**."

The Methods now read:
"For the post-saccadic analysis (Fig S12), large saccades (≥7° net displacement) occurring during head movement were classified as back-and-forth sequences when they were followed within 150 ms by at least two additional smaller saccades, with >90° direction reversal between consecutive events and at least one event counter-directed relative to the primary saccade."

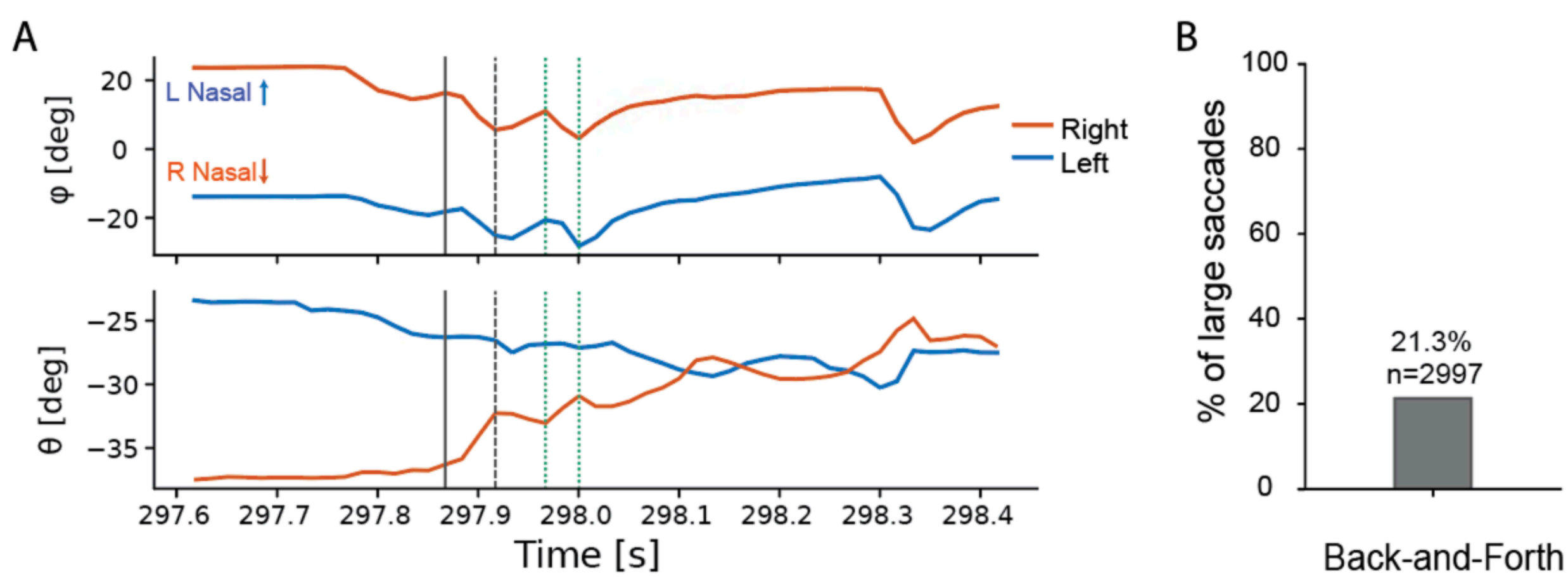

"Fig S12. Post-saccadic back-and-forth sequences during head movement.
(A) Example sequence where both eyes show the 'back-and-forth' reversals. Window −0.25 to +0.55 s around primary onset. Solid: primary onset; dashed: primary offset; green dotted: extras within 150 ms. **(B)** 'Back-and-forth' rate among large saccades during head movement."

Minor comments:

Comment #14: p.1, l. 3: "decision": There are also gaze-shifts that occur relatively automatically (without "conscious" decision), such as reflexes during locomotor orienting behavior, so claiming that a decision is present for every gaze shift is maybe misleading for many readers.

To increase the accuracy of the text we revised this sentence:
"Shifting gaze is fundamental to visual exploration and processing [1,2]. **Visually guided gaze shifts selectively acquire information from specific regions of the visual environment**. In foveated animals [3], this process is particularly precise and informative, offering a unique window into the dynamic prioritization of goals across contexts [4,5].

Comment #15: p.1, l.29: introduce Fusion360 properly. Is it called Autodesk Fusion nowadays?

We thank the reviewer for their careful reading! It is indeed Autodesk Fusion, and we have updated all references to the proper title.

Comment #16: Figure 1e: Micrometer on camera chip? Or Micrometer on imaged sample (eyes)? Please specify. In the methods it is stated that it is the latter, but it is unclear in the main ms text.

We have added this specification to the main text:
"The typical measured jitter was small (overall span 0–200μm **at the imaged eye plane** with a median of 42μm (~1 pixel) for rigid lizard headmounts) (Fig 1E and S9) and could be corrected by subtracting the jitter amplitude."

Comment #17: p.5 l. 5 : "biased towards smaller amplitudes" --> relative to what other amplitude?

We have revised the text to clarify this:
"We next compared the independently normalized amplitude distributions of monocular and concurrent saccades. **While small-amplitude saccades were more frequent than large-amplitude events in both distributions, monocular saccades were typically smaller than concurrent events** (Fig 2G)."

Comment #18: Figure 2c: is camera speed quick enough to measure true peak eye velocity? Provide info on camera frame rate and mention in the methods section.

We have added the "Spatiotemporal limitations" section to the Methods with quantification of the system's limitations in measuring velocity, as detailed in our response to Reviewer 1, Comment #3.

Comment #19: Figure 2h: label axes (pre and post)

The plots show post-saccadic eye endpoint densities relative to the pre-saccadic eye position; Thus, the pre-saccadic position is always located at the origin (0,0) and the heatmap represents

the distribution of post-saccadic positions. We have clarified this in the figure caption which now reads:
"Heatmaps of post-saccadic eye-endpoint densities for concurrent and monocular saccades (single animal), expressed relative to the pre-saccadic eye position (0,0) and plotted in degrees on the unrotated camera axes."

Comment #20: Show noise structure of eye position recordings? Right now, only zoomed-out time traces are shown and it is difficult to judge the noise level of the eye position recordings.

We have added a quantitative characterization of the eye-position noise during stationary eye periods as Fig. S10. This analysis directly compares the measured eye tracking noise and compares it with the saccade-detection threshold. In the revised manuscript we refer to this analysis in the Results and in the new "Spatiotemporal limitations" section of the Methods (see Reviewer 1, Comment #3).

Comment #21: Are there corrective mono-bino saccades (first mono then the other eye as well)?

To answer the reviewer's question, we added a quantitative analysis of the suggested saccade subtype and discuss it in the revised manuscript. The Results now read:
"Only 3.7% of monocular saccades were followed by a directionally aligned contralateral saccade within 80 ms, indicating that the vast majority were not subsequently corrected by the other eye."

The Methods now read:
"To test whether monocular saccades were followed by a corrective movement of the contralateral eye, we identified contralateral saccades occurring within 80 ms of monocular saccade onset and within 45° of its movement direction. Such corrective mono-binocular sequences were rare, occurring in only 3.7% of 15,070 monocular events."

Comment #22: p.5, l. 12: What is the timing of head motion relative to saccades? Do saccades occur before or after the head movement?

We thank the reviewer for this interesting question. We have quantified the timing of head movement onset relative to saccade onset. We added the analysis to Results:
"To support the interpretation that some saccades reflect active gaze redirection rather than purely vestibulo-ocular stabilization, we asked whether they are observed when the head is still. To do so, we recorded head kinematics using a head-mounted accelerometer and identified movement and no-movement epochs (Methods). When head movements were coupled to saccades, they tended to precede saccade onset (median: 78.1 ms). Both head-leading (59.4%) and saccade-leading (36.3%) sequences were common (with only 4.3% of events of simultaneous $|dt| \leq 17$ ms saccades and head movement onset). While most saccades occurred during periods of head movement, a considerable fraction were detected during head-stationary periods (33.2 ± 9.4%, mean ± SEM; n = 15,613 events, 4 animals)."

The above analysis was elaborated in the Methods:

“Head movement and saccade onset

To quantify the timing of head movement relative to saccades, accelerometer movement samples were grouped into bouts, with consecutive samples separated by >40 ms assigned to separate bouts; head movement onset was defined as the first sample of each bout. Concurrent left- and right-eye saccades with onsets within 34 ms were treated as a single event, timed at the earlier onset. For each saccade, the nearest head movement onset within ±500 ms was identified and its signed latency relative to saccade onset was calculated ($\Delta t = t\_head - t\_saccade$; negative values indicate head-leading events). Events within one video frame (±17 ms) were classified as simultaneous. This analysis included 18 recording blocks from four animals for which accelerometer-derived movement annotations were available.”

Comment #23: p.5, l.28: what is the main axis here and how was it determined?

We have revised the text to state the explicit definition of ‘main axis’:

“We therefore rotated distributions to align to the main movement axis, defined as the axis of the maximal number of saccade events for each eye. This revealed that a bias towards a specific axis existed for concurrent saccades across animals (Fig 2I and 2J).”

Comment #24: Figure legend 3d-f: ISI and pupil size panels are mixed up.

We thank the reviewer for this observant point. We corrected the Fig 3 legend.

Comment #25: Also: Do the ISI show the active or the quiescent time points, or both?

To clarify this, revised Fig 3F caption:

“Inter-saccade interval (ISI) distributions for **combined Active and Quiet states** (all data). ISI probability histograms shown on linear (top) and logarithmic (bottom) scales, with overlapping domains between 200-400ms. Each trace represents one animal with the average over animals in black.”

Comment #26: I recommend showing the ISI distributions for active and quiescent periods separately, since this is a major finding of the paper.

Following the reviewer’s recommendation, we added a state-separated analysis of inter-saccadic intervals (Fig S11). The Results section now reads:

“Notably, prolonged quiescent periods, devoid of any detectable eye movements, lasting up to several tens of seconds, were common (Fig 3A and 3D), suggesting discrete shifts between exploratory and stationary visual states**. Separating ISI distributions by behavioral state confirmed that these long intervals were associated predominantly with Quiet periods: while the short-ISI distributions were broadly similar between states, Quiet periods showed a substantially greater prevalence of multi-second inter-saccadic intervals (Fig S11A).**”

A

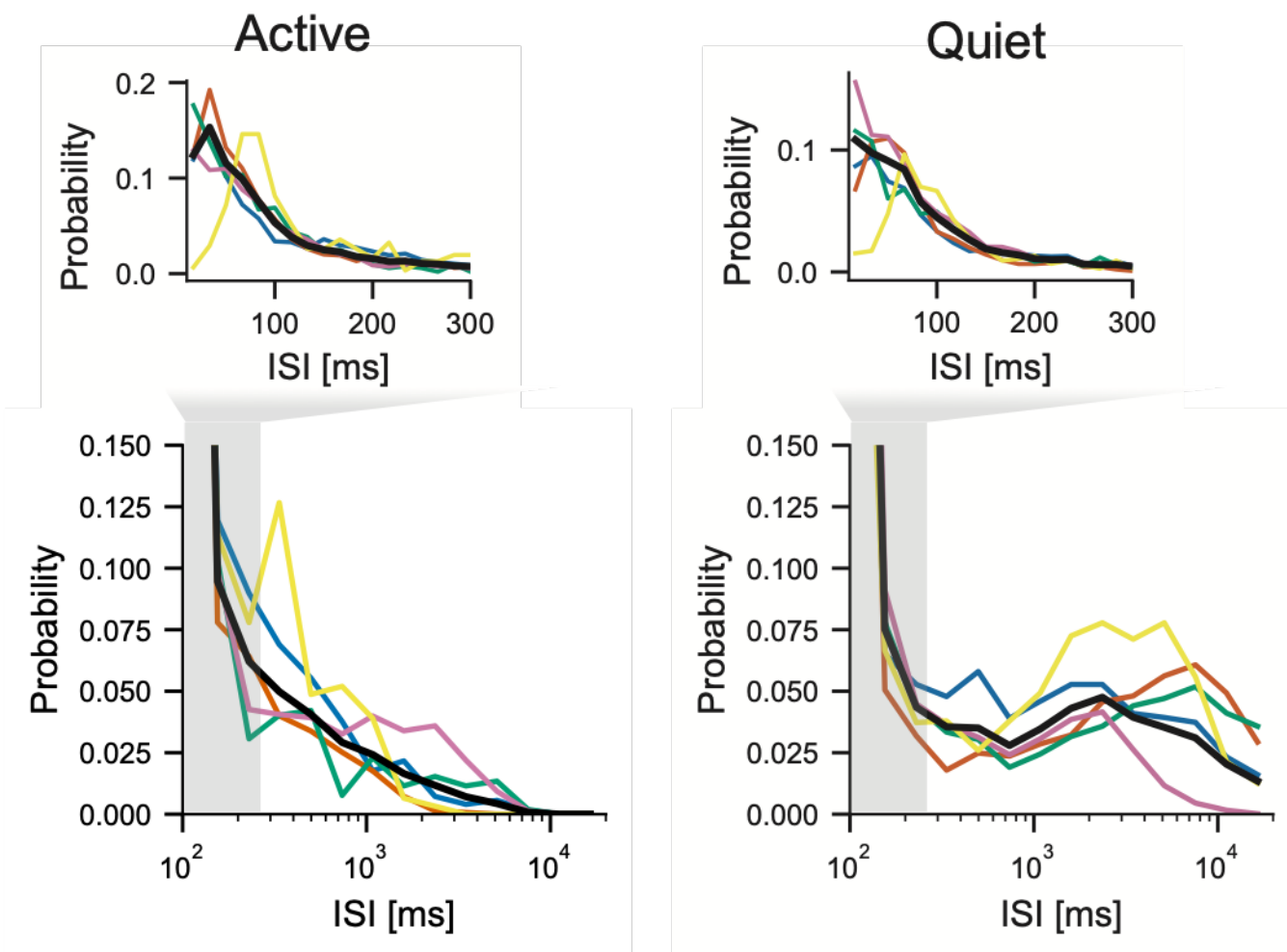

“Fig S11. Inter-saccadic interval distribution in Active Vs. Quiet epochs, and epoch length distributions. (A) Inter-saccadic interval (ISI) distributions separated by behavioral state (left=Active, right=Quiet). Intervals spanning a transition between Active and Quiet were excluded. Colors denote individual animals.”

Comment # 27: p.6, l.40f.: another possible reason: locomotor and postural movement range! The vestibular system keeps the head roll- and pitch-stable, while yaw rotations occur during orienting turns of the animals. As a result, horizontal saccades might be overrepresented evolutionarily. (see e.g. https://pubmed.ncbi.nlm.nih.gov/28607486/ for this evolutionary argument in the visual sensory modality of mice and https://pubmed.ncbi.nlm.nih.gov/41582624/ for eye movements of teleosts).

We thank the reviewer for this hypothesis and agree that locomotor and postural constraints provide an additional plausible explanation for the observed directional anisotropy. We have incorporated this possibility into the Discussion and cited the references.
“Such anisotropy may reflect a combination of biomechanical constraints of the oculomotor plant [71,72] and the nonuniform distribution of behaviorally relevant visual targets across the sampled environments [73], or the effect of the locomotor and postural movement range of the animal. Stabilization of head roll and pitch during locomotion, while yaw rotations occur during orienting turns, may favor an evolutionary overrepresentation of horizontal eye movements [74,75].

Comment #28: Absolute eye positions relative to ground are unclear (since head posture wasn't tracked). This means that we are only looking at relative eye positions. This should be stated more clearly in the main text.

We have now clarified this distinction in the Results text:
“Because head posture relative to the environment was not reconstructed, our analyses quantify relative eye-in-head positions rather than absolute gaze direction in world coordinates.

Comment #29: What type of eye movements are associated with moisturizing the eye (e.g. shortly before the nictitating membrane covers the eye)?

We did not specifically analyze eye movements associated with blinking or nictitating-membrane movements and have clarified their exclusion from the eye-movement analysis in the Methods: "Frames with low DeepLabCut likelihood (< 0.9), including periods in which the pupil was occluded by blinking or movement of the nictitating membrane, were excluded from subsequent eye-movement analyses."

Comment #30: p.6 l.29: movements --> head movements?

Here "movement" refers to the accelerometer-defined movement epochs rather than specifically to head movements. We revised the text to make this distinction clearer:
"Focusing on *P. vitticeps*, we found a rich oculomotor repertoire comprising gaze stabilizing saccades during **movement epochs** with axial biases, and exploratory monocular and binocular saccades during stationary epochs (Fig 2)."
