# Supplementary material for: An open-source eye-tracking system adaptable to diverse terrestrial vertebrates reveals state-dependent visual acquisition in lizards
Source: PLoS Biol. 2026 Nov 3;24(11):e3004066. doi: 10.1371/journal.pbio.3004066 (PMC13754232; doi:10.1371/journal.pbio.3004066)
Supplement: Peer review history

# Editor report: Decision Letter 0

**DOI:** 10.1371/journal.pbio.3004066.r001

**Authors:** Richard Hodge

17 Apr 2026

Dear Dr Shein-Idelson,

Thank you for submitting your manuscript entitled "A personalized eye-tracking system reveals complex state-dependent visual acquisition in lizards" for consideration as a Methods and Resources Article by PLOS Biology.

Your manuscript has now been evaluated by the PLOS Biology editorial staff, as well as by an academic editor with relevant expertise, and I am writing to let you know that we would like to send your submission out for external peer review.

However, before we can send your manuscript to reviewers, we need you to complete your submission by providing the metadata that is required for full assessment. To this end, please login to Editorial Manager where you will find the paper in the 'Submissions Needing Revisions' folder on your homepage. Please click 'Revise Submission' from the Action Links and complete all additional questions in the submission questionnaire.

Once your full submission is complete, your paper will undergo a series of checks in preparation for peer review. After your manuscript has passed the checks it will be sent out for review. To provide the metadata for your submission, please Login to Editorial Manager (https://www.editorialmanager.com/pbiology) within two working days, i.e. by Apr 19 2026 11:59PM.

If your manuscript has been previously peer-reviewed at another journal, PLOS Biology is willing to work with those reviews in order to avoid re-starting the process. Submission of the previous reviews is entirely optional and our ability to use them effectively will depend on the willingness of the previous journal to confirm the content of the reports and share the reviewer identities. Please note that we reserve the right to invite additional reviewers if we consider that additional/independent reviewers are needed, although we aim to avoid this as far as possible. In our experience, working with previous reviews does save time.

If you would like us to consider previous reviewer reports, please edit your cover letter to let us know and include the name of the journal where the work was previously considered and the manuscript ID it was given. In addition, please upload a response to the reviews as a 'Prior Peer Review' file type, which should include the reports in full and a point-by-point reply detailing how you have or plan to address the reviewers' concerns.

During the process of completing your manuscript submission, you will be invited to opt-in to posting your pre-review manuscript as a bioRxiv preprint. Visit http://journals.plos.org/plosbiology/s/preprints for full details. If you consent to posting your current manuscript as a preprint, please upload a single Preprint PDF.

Feel free to email us at plosbiology@plos.org if you have any queries relating to your submission.

Kind regards,

Richard

Richard Hodge, PhD

Senior Editor, PLOS Biology

rhodge@plos.org

PLOS

Empowering researchers to transform science

Carlyle House, Carlyle Road, Cambridge, CB4 3DN, United Kingdom

California (U.S.) corporation #C2354500, based in San Francisco

# Peer review: Decision Letter 1

**DOI:** 10.1371/journal.pbio.3004066.r002

**Authors:** Richard Hodge

29 May 2026

Dear Dr Shein-Idelson,

Thank you for your patience while your manuscript "A personalized eye-tracking system reveals complex state-dependent visual acquisition in lizards" was peer-reviewed at PLOS Biology. It has now been evaluated by the PLOS Biology editors, an Academic Editor with relevant expertise, and by several independent reviewers.

In light of the reviews, which you will find at the end of this email, we would like to invite you to revise the work to thoroughly address the reviewers' reports.

As you will see below, the reviewers found your manuscript very interesting and say that it would represent an important methodological development if the adaptability to other species can be confirmed (Reviewer 2). They also request to provide more methodological details within the manuscript to make it easier for other researchers to use your approach.

Given the extent of revision needed, we cannot make a decision about publication until we have seen the revised manuscript and your response to the reviewers' comments. Your revised manuscript is likely to be sent for further evaluation by all or a subset of the reviewers.

In addition to these revisions, you will need to complete some formatting changes, which you will receive in a follow up email. A member of our team will be in touch with a set of requests shortly.

We expect to receive your revised manuscript within 3 months. Please email us (plosbiology@plos.org) if you have any questions or concerns, or would like to request an extension.

At this stage, your manuscript remains formally under active consideration at our journal; please notify us by email if you do not intend to submit a revision so that we may withdraw it.

**IMPORTANT - SUBMITTING YOUR REVISION**

Your revisions should address the specific points made by each reviewer. Please submit the following files along with your revised manuscript:

1. A 'Response to Reviewers' file - this should detail your responses to the editorial requests, present a point-by-point response to all of the reviewers' comments, and indicate the changes made to the manuscript.

*NOTE: In your point-by-point response to the reviewers, please provide the full context of each review. Do not selectively quote paragraphs or sentences to reply to. The entire set of reviewer comments should be present in full and each specific point should be responded to individually, point by point.

You should also cite any additional relevant literature that has been published since the original submission and mention any additional citations in your response.

2. In addition to a clean copy of the manuscript, please also upload a 'track-changes' version of your manuscript that specifies the edits made. This should be uploaded as a "Revised Article with Changes Highlighted" file type.

*Re-submission Checklist*

When you are ready to resubmit your revised manuscript, please refer to this re-submission checklist: https://plos.io/Biology_Checklist

To submit a revised version of your manuscript, please go to https://www.editorialmanager.com/pbiology/ and log in as an Author. Click the link labelled 'Submissions Needing Revision' where you will find your submission record.

Please make sure to read the following important policies and guidelines while preparing your revision:

*Published Peer Review*

Please note while forming your response, if your article is accepted, you may have the opportunity to make the peer review history publicly available. The record will include editor decision letters (with reviews) and your responses to reviewer comments. If eligible, we will contact you to opt in or out. Please see here for more details:

https://blogs.plos.org/plos/2019/05/plos-journals-now-open-for-published-peer-review/

*PLOS Data Policy*

Please note that as a condition of publication PLOS' data policy (http://journals.plos.org/plosbiology/s/data-availability) requires that you make available all data used to draw the conclusions arrived at in your manuscript. If you have not already done so, you must include any data used in your manuscript either in appropriate repositories, within the body of the manuscript, or as supporting information (N.B. this includes any numerical values that were used to generate graphs, histograms etc.). For an example see here: http://www.plosbiology.org/article/info%3Adoi%2F10.1371%2Fjournal.pbio.1001908#s5

*Blot and Gel Data Policy*

We require the original, uncropped and minimally adjusted images supporting all blot and gel results reported in an article's figures or Supporting Information files. We will require these files before a manuscript can be accepted so please prepare them now, if you have not already uploaded them. Please carefully read our guidelines for how to prepare and upload this data: https://journals.plos.org/plosbiology/s/figures#loc-blot-and-gel-reporting-requirements

*Protocols deposition*

To enhance the reproducibility of your results, we recommend that if applicable you deposit your laboratory protocols in protocols.io, where a protocol can be assigned its own identifier (DOI) such that it can be cited independently in the future. Additionally, PLOS ONE offers an option for publishing peer-reviewed Lab Protocol articles, which describe protocols hosted on protocols.io. Read more information on sharing protocols at https://plos.org/protocols?utm_medium=editorial-email&utm_source=authorletters&utm_campaign=protocols

Thank you again for your submission to our journal. We hope that our editorial process has been constructive thus far, and we welcome your feedback at any time. Please don't hesitate to contact us if you have any questions or comments.

Sincerely,

Christian

Christian Schnell, Ph.D.

Senior Editor

PLOS Biology

cschnell@plos.org

on behalf of

Richard Hodge, PhD

Senior Editor

PLOS Biology

rhodge@plos.org

------------------------------------

REVIEWS:

Reviewer #1: Leberstein et al present a detailed and innovative study outlining a lightweight, customizable eye tracking system amenable across the terrestrial animal species. Their system is clearly useful in mouse, turtles, and lizards--with a deeper exploration into oculomotor and binocular behaviors of freely moving lizards. The paper is well written and clear, difficult given the difficulties in implementing these types of hardware and software/analyses needed to estimate egocentric gaze. In addition, I think it's an excellent resource just in terms of the exhaustive list of historical and recent papers referenced in the field of freely-moving animal eye tracking (I will be asking students in my lab to use this paper as a reference). I consider my comments minor, but I would like to see them address in a revised version of this manuscript.

Comments:

- There is a lack of details on the surgical implantation and I would like to learn/see more. Perhaps some images as a supplemental are all that are required, but this seems important given that different methods of implanting the hexagonal screw anchor across animals.

- The photogrammetry is clever, but I wonder how well this solution works for animals with large amounts of skin or muscle on top of their head. How important is it to estimate the skull-eye relationship for this technique to be useful?

- Camera specs are important and these details should be directly added to the main text. In addition, I was wondering if a framerate of 60 Hz and the pixel resolution used, given the eye size on the sensor, had any limitations in detecting certain speed of saccades?

- Please specify the source of many parts. For example, the source of the IR mirrors as there are many companies producing these size mirrors and not all are the same quality.

- is there anything to be gained by analyzing the corneal reflections of the two IR LEDs? This is a technique that can be used to increase accuracy of eye position and potentially estimate gaze-in-world. The fixed position of these LEDs and the stability of the head mount seems great for this kind of tracking of corneal reflections.

- fig 2b: Can you please add some anatomical directional indicators (e.g. nasal/temporal or something similar)? Also, could indicate the interest binocular conditions described in the text (line 32-33) to help readers?

- fig 3a-b: x axes in seconds?

- Can you provide more details of how quiet/active periods were assessed? What happens if only a single 1 second window is labeled active in a long period of quiet? How long are active periods (they appear shorter than quiet ones)?

Reviewer #2: The manuscript by Leberstein and colleagues introduces a personalized eye tracking system as a versatile method to study eye movements in freely moving animals. Using a combination of photogrammetry, 3D-modelling and -printing, the authors produce head mounts adaptable for diverse head-eye geometries and small vertebrate species (here: mouse, turtle, and lizard). The authors then use this system to provide a systematic characterization of eye movements in the lizard Pogona vitticeps. Stable, high-resolution recordings of eye movements in freely moving animals are technically challenging, yet can provide critical information for (neuro)biological studies. A system that is easily adaptable to different species, reliable, and accessible to a broad scientific community, would represent an important contribution, and I am excited by the prospect of seeing this method being adopted and used in many labs and for studies in different species.

First of all, I want to applaud the authors for the quantitative analysis of eye movements in Pogona vitticeps, which I found both interesting and well executed, also representing a major part of the manuscript.

That said, I have been tasked to review this manuscript as a "Methods and Resources" article, and my evaluation thus focuses on the methodological approach, its novelty and potential to ask previously inaccessible biological questions, and to qualitatively compare it to existing systems. With this in mind, I am not yet convinced, and even though the method may well meet the necessary criteria, it needs to be demonstrated more clearly. However, I do suspect the necessary data may be there already, at least in part, or can be collected within a reasonable timeframe for a revised manuscript.

Specifically, one main strength of the approach would be its promised versatility and flexibility. The authors show images of three species carrying the head mount system, and they suggest they have successfully recorded eye movements in all three (that is, M. musculus, T. scripta elegans, and P. vitticeps). But that hasn't been shown, it is unclear what "successful" means, and how the data looks like and compares to that obtained with previously published methods. - The images in Fig.1f don't tell us much, and all other figures and data are specific to Pogona. To allow for a comparison with existing methods, and to actually demonstrate the system's versatility and usefulness to the broader scientific community, it is essential to provide additional analyses at least for the mouse, and hence a widely used model for which various other systems have been introduced in the past. Moreover, though perhaps less critical in this context, this would also permit a direct comparison of visual acquisition strategies in a reptile and a mammal, using the same (novel) approach, strengthening the author's scientific conclusions and interpretation of their data.

Another important aspect is the optional use of a "modular solution kit", which Leberstein and colleagues introduce as an alternative by which the fitting procedure can be performed directly on the animal. This would represent a major advantage, because photogrammetry may indeed often not be an option, and a modular solution would make the whole method much more accessible, certainly for people working with common rodent models, but also for others. To demonstrate this, it would be essential for the authors to provide some direct comparison with their rigid system in terms of stability and data quality/noise etc. (Note: By analyzing the existing mouse data, the authors could probably do much of it in one go, as they have collected these with the modular system; though ideally on could also show a direct comparison within the same species, such as the lizard).

Minor comments:

-Comparing a mouse and lizard species does of course not allow for conclusions about possibly general differences in visual acquisition strategies between ecto- and endotherms. This would require a much broader sampling, and the authors should hence tone down their conclusions/motivation to make this clear.

But, the Pogona data provides a first hint such differences might exist, which is interesting. Along those lines, adding data from the turtle could help in this respect, provided it matches the overall patterns observed in the distantly related lizard species (i.e. it could suggest a more general reptilian phenotype).

-It wasn't clear to me what the difference is between Fig. 2f and S3 (left panel). In both cases, are we looking at inter-ocular peak-speed coupling? If so, is S3 simply a zoomed-in version? I am probably missing something, but why does the density of the points seem to differ, and why do the probabilities differ by a factor of 10 (colour bar)?

-Fig. 3: Figure captions d to f were mixed up and don't match with the legend below.

Reviewer #3: In this study, the authors developed technological solutions for tracking eye movements in two dimensions for freely moving terrestrial vertebrates of varying head shapes. The authors then use this technique to characterize eye movements of the lizard P. vitticeps, especially saccades. Lizard saccades show similarity to human saccades in terms of speed profiles and speed-amplitude dependence ("main sequence"), which isn't entirely surprising, but a useful characterization. The authors then move on to show that lizard saccades (somewhat similar to the situation in the chameleon) show many monocular saccades (especially small-amplitude ones) as well as binocularly coordinated saccades. The monocularly coordinated saccades do occur in any direction ("isotropic"), while the binocularly coordinated saccades show movement mainly along one orientation (that likely corresponds to the horizontal plane). These findings are intriguing as they show that the lizard (unlike e.g. humans) readily performs monocular as well as binocular saccades, which is discussed to potentially be related to different proximal functions (sampling vs. reflexive gaze shifting). Finally, the authors add head acceleration measurement to identify quiet and active periods (locomotion/posture). Saccade rate is much higher during active periods and pupil size scales inversely with locomotor activity. These results show that the lizard eye movements during awake times follow different behavior states, which provides interesting links to related processes and sleep in other species. Given that lizards are ectothermic and eye movements and locomotor activity are related to energy consumption, the provided characterization is a valuable addition to the literature, as the results will inform further comparative studies on the functional role and evolutionary optimization of eye movements. The authors also discuss the observed eye movements and the situation in the lizard as an opportunity to study "attention" in an evolutionary ancient vertebrate. However, I do not find this claim very convincing in the absence of evidence for "attentive" behavior in this study.

The provided analysis is very sound and the statistical methods appear appropriate.

This is a well-rounded study on eye movements in a non-canonical animal model that fills an important gap in the literature and furthermore provides technical solution examples for eye tracking that can likely be adapted to other animal species easily.

I also have a few more detail comments and criticisms, which I provide below.

Major comments:

1)Analysis/Illustration caveat: In Figure 2f, why do conjugate saccades have such small amplitude (speed) compared to monocular ones (In the text you state the opposite)? In conjunction with Figure 2g: why are monocular saccades so frequent (is it due to detection noise? - the label "concurrent" is less likely to occur for random data than the label "monocular", this could explain the high peak for small velocity monocular saccades)? p.5, l. 7: it is stated that concurrent saccades are of larger amplitude, but Figure 2f in combination with 2d imply the opposite…much fewer large saccades along the line of unity are present. Do monocular and binocular saccades have the same main sequence? In Fig 2e, it would be helpful to plot the individual saccade data points in the background.

2)Results in Figure 1 are pitched as a general technical solution that works for many species. I believe that this is true, however, I'd appreciate if the authors put in more effort to describe the difficulties and important points of consideration in the main text. This would help future scientists to estimate whether the planned approach (for a new species) would likely work or not. For example, a) What are the limitations with regard to head shape for headmounts, b) What type of skull (thicknesses) allow screw anchoring/implantation? c) Please describe, for how long the implants were stable and what the latest time points (hours/days after implantation) were for the successful recordings. d) for what type of eye morphologies/pupil contrasts will this method work?

3)Angular eye position detection errors were likely up to 5° in size (p. 16, l26). This is important information and should be stated in the main text.

4)What fraction/types of detected saccades are likely artifacts or behavior not related to an intended eye position change? E.g. in the video S1, the saccade is sometimes followed (within milliseconds) by a saccade back to the original starting position. Are these "jerks", in which eye movements result from the animal trying to do something else than moving its eyes? If not, what could be the function of such eye movements? Is eye tracking stable during locomotion or do eye position artifacts occur (e.g. due to wobble of the hardware assembly)?

5)p.7 l.35: "attention" research possible in P. vitticeps: This is an interesting possibility. However, the authors did not show that the saccades in P. vitticeps are goal-directed or "voluntary" and it seems possible that they are "spontaneous" scanning saccades. Therefore, the relation of the observed eye movements to "attention" is not straightforward. This should be discussed more carefully or toned down. Also, the title sounds a bit overstated to the ears of the reviewer ("complex state-dependent visual acquisition"). To uphold this claim, the authors should show that the different types of eye movements are task-associated, e.g. by showing an intricate relation of eye movements and visual stimuli and/or locomotion.

6)In the video S1 the reviewer observed a nystagmus-like eye movement (eye moves back-and-forth a couple of times with small amplitude) right after the saccade in a couple of cases. How frequently did this occur and what is a putative purpose of such eye movement? For primates, the eye position would rather remain stable directly after the saccade.

Minor comments:

p.1, l. 3: "decision": There are also gaze-shifts that occur relatively automatically (without "conscious" decision), such as reflexes during locomotor orienting behavior, so claiming that a decision is present for every gaze shift is maybe misleading for many readers.

p.1, l.29: introduce Fusion360 properly. Is it called Autodesk Fusion nowadays?

Figure 1e: Micrometer on camera chip? Or Micrometer on imaged sample (eyes)? Please specify. In the methods it is stated that it is the latter, but it is unclear in the main ms text.

p.5 l. 5 : "biased towards smaller amplitudes" --> relative to what other amplitude?

Figure 2c: is camera speed quick enough to measure true peak eye velocity? Provide info on camera frame rate and mention in the methods section.

Figure 2h: label axes ( pre and post)

Show noise structure of eye position recordings? Right now, only zoomed-out time traces are shown and it is difficult to judge the noise level of the eye position recordings.

Are there corrective mono-bino saccades (first mono then the other eye as well)?

p.5, l. 12: What is the timing of head motion relative to saccades? Do saccades occur before or after the head movement?

p.5, l.28: what is the main axis here and how was it determined?

Figure legend 3d-f: ISI and pupil size panels are mixed up. Also: Do the ISI show the active or the quiescent time points, or both? I recommend showing the ISI distributions for active and quiescent periods separately, since this is a major finding of the paper.

p.6, l.40f.: another possible reason: locomotor and postural movement range! The vestibular system keeps the head roll- and pitch-stable, while yaw rotations occur during orienting turns of the animals. As a result, horizontal saccades might be overrepresented evolutionarily. (see e.g. https://pubmed.ncbi.nlm.nih.gov/28607486/ for this evolutionary argument in the visual sensory modality of mice and https://pubmed.ncbi.nlm.nih.gov/41582624/ for eye movements of teleosts )

Absolute eye positions relative to ground are unclear (since head posture wasn't tracked). This means that we are only looking at relative eye positions. This should be stated more clearly in the main text.

What type of eye movements are associated with moisturizing the eye (e.g. shortly before the nictitating membrane covers the eye)?

p.6 l.29: movements --> head movements?

# Author comment: Author response to Decision Letter 2

**DOI:** 10.1371/journal.pbio.3004066.r003

15 Sep 2026

Attachment Submitted filename: Response_letter_for_reviewers.pdf

# Editor report: Decision Letter 2

**DOI:** 10.1371/journal.pbio.3004066.r004

**Authors:** Richard Hodge

18 Sep 2026

Dear Mark,

Thank you for your patience while we considered your revised manuscript "A personalized eye-tracking system reveals complex state-dependent visual acquisition in lizards" for publication as a Methods and Resources Article at PLOS Biology. This revised version of your manuscript has been evaluated by the PLOS Biology editors and the Academic Editor.

Based on our Academic Editor's assessment of your revision, I am pleased to say that we are likely to accept this manuscript for publication, provided you address the following data and other policy-related requests that I have provided below (A-F):

(A) We routinely suggest changes to titles to ensure maximum accessibility for a broad, non-specialist readership. In this case, we would like to suggest a minor edit to the title, as follows, to further bring out the methodological aspects of the study. Please ensure you change both the manuscript file and the online submission system, as they need to match for final acceptance:

“An open-source, adaptable eye-tracking system enables studies of visual acquisition across diverse terrestrial vertebrates”

(B) You may be aware of the PLOS Data Policy, which requires that all data be made available without restriction: http://journals.plos.org/plosbiology/s/data-availability. For more information, please also see this editorial: http://dx.doi.org/10.1371/journal.pbio.1001797

Note that we do not require all raw data. Rather, we ask that all individual quantitative observations that underlie the data summarized in the figures and results of your paper be made available in one of the following forms:

-Supplementary files (e.g., excel). Please ensure that all data files are uploaded as 'Supporting Information' and are invariably referred to (in the manuscript, figure legends, and the Description field when uploading your files) using the following format verbatim: S1 Data, S2 Data, etc. Multiple panels of a single or even several figures can be included as multiple sheets in one excel file that is saved using exactly the following convention: S1_Data.xlsx (using an underscore).

-Deposition in a publicly available repository. Please also provide the accession code or a reviewer link so that we may view your data before publication.

Regardless of the method selected, please ensure that you provide the individual numerical values that underlie the summary data displayed in the following figure panels as they are essential for readers to assess your analysis and to reproduce it:

Figure 1E, 2B-G, 2I-J, 3A-F, S1A-B, S2, S3, S4, S5, S8-E, S9, S10A-C, S11A-B, S12A-B, S13A-E

NOTE: the numerical data provided should include all replicates AND the way in which the plotted mean and errors were derived (it should not present only the mean/average values).

(C) Please also ensure that each of the relevant figure legends in your manuscript include information on *WHERE THE UNDERLYING DATA CAN BE FOUND*, and ensure your supplemental data file/s has a legend.

(D) Please note that we cannot accept sole deposition of code in GitHub, as this could be changed after publication. However, you can archive this version of your publicly available GitHub code to Zenodo. Once you do this, it will generate a DOI number, which you will need to provide in the Data Accessibility Statement (you are welcome to also provide the GitHub access information). See the process for doing this here: https://docs.github.com/en/repositories/archiving-a-github-repository/referencing-and-citing-content

(E) Please ensure that your Data Statement in the submission system accurately describes where your data can be found and is in final format, as it will be published as written there.

(F) Please ensure that you are using best practice for statistical reporting and data presentation. These are our guidelines https://journals.plos.org/plosbiology/s/best-practices-in-research-reporting#loc-statistical-reporting and a useful resource on data presentation https://journals.plos.org/plosbiology/article?id=10.1371/journal.pbio.1002128

- If you are reporting experiments where n ≤ 5, please plot each individual data point.

------------------------------------------------------------------------

As you address these items, please take this last chance to review your reference list to ensure that it is complete and correct. If you have cited papers that have been retracted, please include the rationale for doing so in the manuscript text, or remove these references and replace them with relevant current references. Any changes to the reference list should be mentioned in the cover letter that accompanies your revised manuscript.

In addition to these revisions, you may need to complete some formatting changes, which you will receive in a follow up email. A member of our team will be in touch with a set of requests shortly. If you do not receive a separate email within a few days, please assume that checks have been completed, and no additional changes are required.

We expect to receive your revised manuscript within two weeks.

To submit your revision, please go to https://www.editorialmanager.com/pbiology/ and log in as an Author. Click the link labelled 'Submissions Needing Revision' to find your submission record. Your revised submission must include the following:

- a cover letter that should detail your responses to any editorial requests, if applicable, and whether changes have been made to the reference list

- a Response to Reviewers file that provides a detailed response to the reviewers' comments (if applicable, if not applicable please do not delete your existing 'Response to Reviewers' file.)

- a track-changes file indicating any changes that you have made to the manuscript.

NOTE: If Supporting Information files are included with your article, note that these are not copyedited and will be published as they are submitted. Please ensure that these files are legible and of high quality (at least 300 dpi) in an easily accessible file format. For this reason, please be aware that any references listed in an SI file will not be indexed. For more information, see our Supporting Information guidelines:

https://journals.plos.org/plosbiology/s/supporting-information

*Published Peer Review History*

Please note that you may have the opportunity to make the peer review history publicly available. The record will include editor decision letters (with reviews) and your responses to reviewer comments. If eligible, we will contact you to opt in or out. Please see here for more details:

https://plos.org/published-peer-review-history/

*Press*

Should you, your institution's press office or the journal office choose to press release your paper, please ensure you have opted out of Early Article Posting on the submission form. We ask that you notify us as soon as possible if you or your institution is planning to press release the article.

*Protocols deposition*

To enhance the reproducibility of your results, we recommend that if applicable you deposit your laboratory protocols in protocols.io, where a protocol can be assigned its own identifier (DOI) such that it can be cited independently in the future. Additionally, PLOS ONE offers an option for publishing peer-reviewed Lab Protocol articles, which describe protocols hosted on protocols.io. Read more information on sharing protocols at https://plos.org/protocols?utm_medium=editorial-email&utm_source=authorletters&utm_campaign=protocols

Please do not hesitate to contact me should you have any questions.

Best wishes,

Richard

Richard Hodge, PhD

Senior Editor, PLOS Biology

rhodge@plos.org

PLOS

Empowering researchers to transform science

Carlyle House, Carlyle Road, Cambridge, CB4 3DN, United Kingdom

California (U.S.) corporation #C2354500, based in San Francisco

# Author comment: Author response to Decision Letter 3

**DOI:** 10.1371/journal.pbio.3004066.r005

1 Oct 2026

Attachment Submitted filename: Response_letter_for_reviewers_auresp_3.pdf

# Editor report: Decision Letter 3

**DOI:** 10.1371/journal.pbio.3004066.r006

**Authors:** Richard Hodge

8 Oct 2026

Dear Mark,

On behalf of my colleagues and the Academic Editor, Tom Baden, I am pleased to say that we can accept your manuscript for publication, provided you address any remaining formatting and reporting issues. These will be detailed in an email you should receive within 2-3 business days from our colleagues in the journal operations team; no action is required from you until then. Please note that we will not be able to formally accept your manuscript and schedule it for publication until you have completed any requested changes.

Please take a minute to log into Editorial Manager at http://www.editorialmanager.com/pbiology/, click the "Update My Information" link at the top of the page, and update your user information to ensure an efficient production process.

PRESS

We frequently collaborate with press offices. If your institution or institutions have a press office, please notify them about your upcoming paper at this point, to enable them to help maximise its impact. If the press office is planning to promote your findings, we would be grateful if they could coordinate with biologypress@plos.org. If you have previously opted in to the early version process, we ask that you notify us immediately of any press plans so that we may opt out on your behalf.

We also ask that you take this opportunity to read our Embargo Policy regarding the discussion, promotion and media coverage of work that is yet to be published by PLOS. As your manuscript is not yet published, it is bound by the conditions of our Embargo Policy. Please be aware that this policy is in place both to ensure that any press coverage of your article is fully substantiated and to provide a direct link between such coverage and the published work. For full details of our Embargo Policy, please visit http://www.plos.org/about/media-inquiries/embargo-policy/.

Thank you again for choosing PLOS Biology for publication and supporting Open Access publishing. We look forward to publishing your study.

Best wishes,

Richard

Richard Hodge, PhD

Senior Editor, PLOS Biology

rhodge@plos.org

PLOS

Empowering researchers to transform science

Carlyle House, Carlyle Road, Cambridge, CB4 3DN, United Kingdom

California (U.S.) corporation #C2354500, based in San Francisco
